# Development and consensus-based validation of a comprehensive population survey instrument for municipal crisis preparedness: a three-round Delphi study (KOMPAKT112)

**DOI:** 10.3389/fpubh.2026.1883344

**Published:** 2026-08-05

**Authors:** Andrea C. Baron, Andreas Batsios, Laura Over-Müller, Cosmo Bulasikis, Ole Franzen, Michael Reppel, Peer Franzen, Kai Mortensen, Daniel Drömann, Patrick Ristau, Klaas F. Franzen

**Affiliations:** 1 Anaesthesiology and Intensive Care Medicine, Schön Klinik, Neustadt in Holstein, Germany; 2 Medical Clinic III, University of Luebeck, Luebeck, Germany; 3 Ministry of the Interior, Municipal Affairs, Housing, and Sports, Kiel, Germany; 4 Stormarn Technical Operations Management, Stormarn District Fire Brigade Association, Travenbrück, Germany; 5 Reinfeld Volunteer Fire Brigade, Reinfeld, Germany; 6 Department of Rhythmology, University of Luebeck, Luebeck, Germany; 7 Cardiology Landsberg, Landsberg am Lech, Germany; 8 Hamburg Fire Brigade, Ministry of the Interior and Sport of the Free and Hanseatic City of Hamburg, Hamburg, Germany; 9 Cardiology Kiel, Kiel, Germany; 10 Airway Research Center North, German Center for Lung Research (DZL), Großhansdorf, Germany; 11 Faculty of Applied Health Sciences, Deggendorf Institute of Technology, Deggendorf, Germany

**Keywords:** civil protection, community resilience, CREDES, crisis preparedness, Delphi technique, emergency preparedness, instrument development, population survey

## Abstract

**Introduction:**

Valid multidimensional instruments for assessing crisis preparedness at the municipal population level are largely unavailable. Nationally representative surveys cannot provide the subnational granularity required for locally targeted civil protection planning. We developed and validated KOMPAKT112, which is, to our knowledge, the first all-hazards, multidimensional preparedness questionnaire designed for German municipal civil protection research.

**Methods:**

A three-round modified Delphi study (October-November 2025) was conducted with 26 interdisciplinary experts from eight domains: civil protection authorities, fire brigades, THW, aid organizations, the German Armed Forces, church communities, municipal administration, and academia. An initial 183-item pool was generated from seven preparedness frameworks, rated on a five-point Likert scale, with an *a priori* consensus threshold of ≥75%. Reporting followed the CREDES guidelines. External validation comprised a cognitive pre-test (*n* = 12, think-aloud protocol) and a population-based pretest (*n* = 50).

**Results:**

Response rates were 100% (Rounds 1-2) and 92.3% (Round 3). The item pool decreased from 183 to 132 items across 14 themes. The mean block-level consensus increased monotonically from 78.9 to 91.2%, and the mean interquartile range converged from 1.60 to 0.77. The overall Content Validity Index was 0.91 (range, 0.85–0.94), exceeding the ≥0.80 threshold across all blocks. Cognitive pretesting prompted revisions to the six blocks. The population-based pre-test confirmed score differentiation, absence of floor/ceiling effects, and a knowledge–behavior gap of 7.3 percentage points (knowledge: 76.2% vs. behavior: 68.9%). Seventy percent of the pre-test participants reported increased crisis preparedness awareness following completion.

**Discussion:**

KOMPAKT112 demonstrated robust content validity and is suitable for population-level municipal crisis preparedness research in Germany. Its 14-block architecture uniquely covers civil-military cooperation perception and volunteer engagement readiness, which are dimensions absent from existing instruments, including the HEPI. The instrument is freely available as Supplementary material and constitutes a promising, content-validated candidate instrument for comparative studies on municipal preparedness.

## 1 Introduction

The civil protection landscape in Germany and across Europe faces an increasingly complex threat environment comprising large-scale power failures, pandemics, cyberattacks on critical infrastructure, and hybrid geopolitical threats, in addition to classical natural hazards (1–4). European stakeholders have identified the transition toward compound, cascading multi-hazard risks encompassing climate extremes, biological emergencies, and critical infrastructure disruption as a defining governance challenge requiring integrated all-hazards approaches (4). This diversification demands resilience across all societal levels, from professional emergency response organizations to individual household preparedness (5). The 2024 Special Eurobarometer on disaster preparedness (*n* > 26,000) found that 65% of EU citizens needed more information to prepare, and fewer than 29% maintained basic emergency food stockpiles (6). In Germany, persistent behavioral gaps in household preparedness coexist with heightened awareness following the COVID-19 pandemic and the 2021 Ahr Valley flood (7, 8). This discrepancy between awareness and enacted behavior, which means the preparedness knowledge–behavior gap, is recognized as a central challenge for civil protection policies (9, 10). A systematic search of PubMed, Google Scholar, and the BBK publication archive using the terms “Bevölkerungsschutz”, “Krisenvorsorge”, “Fragebogen”, “Erhebungsinstrument”, “disaster preparedness”, “survey instrument”, and “civil protection” identified no published, peer-reviewed, validated German-language preparedness survey instrument designed for municipal application that simultaneously covers warning system familiarity, civil-military cooperation perception, volunteer engagement readiness, and household preparedness behavior in a single, all-hazards tool developed through an interdisciplinary expert consensus process. Bridging this gap requires locally specific empirical evidence on knowledge, behavioral, and motivational barriers, which nationally representative surveys cannot provide (11).

A growing body of validated preparedness instruments is available internationally. The Household Emergency Preparedness Instrument (HEPI), developed through an international Delphi process (*n* = 154 panelists across 36 countries), is the most rigorously validated all-hazards household tool, comprising 51 items and demonstrating strong internal consistency (Cronbach’s *α* = 0.96) (12). The Emergency Preparedness Checklist (EPC) has demonstrated psychometric robustness in multiple United States studies (13). In Germany, the BBK population surveys and Allensbach preparedness studies provide nationally representative data (8, 11, 14), but lack the municipal resolution required for localized civil protection planning. A scoping review of governmental preparedness evaluation instruments found no consensus on measurement metrics and documented widespread limitations in their local applicability (11). No validated German-language instrument covers the full multi-dimensional spectrum of municipal preparedness, which includes warning system familiarity, civil-military cooperation perception, and volunteer engagement readiness, in a single, all-hazards tool designed for interdisciplinary expert consensus across the German civil protection architecture (BOS, Bundeswehr, church communities, municipal administration).

The Delphi method is a structured, iterative expert consultation process designed to achieve group consensus through anonymized, controlled feedback rounds (15). It is well-suited to instrument development in domains where evidence is incomplete and interdisciplinary consensus is required (16). The Conducting and Reporting Delphi Studies (CREDES) (17) provides reference standards for reporting. The KOMPAKT112 (Kommunaler Präventions- und Katastrophenschutz [Municipal prevention and civil protection]) project is a formal research partnership between the Universität zu Lübeck (Medical Clinic III), the Stadt Reinfeld (Holstein), and the Amt Nordstormarn, Schleswig-Holstein, Germany. This study reports the Delphi-based development and validation of the project’s core survey instrument, which constitutes a standalone methodological contribution independent of the forthcoming main survey findings. The instrument is publicly available in Supplementary material.

## 2 Methods

### 2.1 Study design and justification

We employed a modified three-round Delphi method. It was modified in that the initial item pool was generated through a systematic literature review (rather than open-ended expert elicitation), and rounds were conducted asynchronously via LimeSurvey (version 6.x) hosted on university-administered servers (18). The Delphi technique was selected over nominal group or committee consensus methods for three reasons: (1) no existing validated instrument covered all required dimensions in the German municipal context; (2) the interdisciplinary character of civil protection precluded reliance on a single disciplinary tradition; and (3) the geographic and scheduling constraints of operational experts made asynchronous delivery necessary. The three-round structure was selected in accordance with established practices in Delphi studies on instrument development (12, 17). Reporting was performed according to the CREDES checklist (Supplementary file S3).

### 2.2 Expert panel

Experts were identified through purposive sampling across five qualification domains: (1) leadership or training experience in a emergency service organization (German: Behörden und Organisationen mit Sicherheitsaufgaben, BOS) like fire brigades, THW (Federal Agency for Technical Relief), or emergency medical services; (2) active or former Bundeswehr service relevant to domestic disaster operations; (3) scientific expertise in disaster medicine, emergency public health, social resilience, or risk communication; (4) representative function in municipal civil protection administration; and (5) representative function in a church or faith community with documented civil society resilience relevance. Recruitment proceeded from the project’s established stakeholder network, extended via snowball sampling into academic and military networks. All the panelists provided informed consent. No financial compensation was provided for this study. A minimum of 20 panelists across at least six domains were targeted to ensure cross-sectoral representativeness (19).

### 2.3 Item pool generation

A literature-based initial item pool was constructed from seven source categories: (a) BBK preparedness guidelines (2021) and Bevölkerungsschutzbarometer (2022-2023) (8); (b) Deutsche Resilienzstrategie 2022 (20); (c) HEPI item bank (12); (d) Special Eurobarometer 547 (6); (e) FEMA National Household Survey (21); (f) peer-reviewed literature on preparedness measurement and knowledge–behavior gaps (9, 13); and (g) project team expertise in civil-military cooperation and BOS-specific knowledge domains. Items were organized into 14 *a priori* thematic blocks reflecting the major dimensions of the target construct.

### 2.4 Delphi rounds

In Round 1, the panelists rated each item’s relevance on a 5-point Likert scale (1 = not at all relevant; 5 = extremely relevant), and had the option to propose reformulations and suggest new items. In Round 2, the panelists received anonymized group-level feedback (median and interquartile range [IQR] per item from Round 1) alongside the revised item set and provided a second relevance rating and explicit comprehensibility assessment (3-point scale: clearly comprehensible, partially comprehensible, and not comprehensible). This dual-rating design disentangled relevance judgments from concerns about item clarity. Round 3 was a confirmatory editorial review. The panelists confirmed the completeness of the block structure and the suitability of the response formats without further item reduction.

### 2.5 Consensus definition and decision rules

The *a priori* consensus threshold was ≥75% of the panelists’ rating ≥4 on the five-point scale. This is the upper end of the range commonly reported in health Delphi studies (60–80%) (17–19), which is consistent with the HEPI development study (12). Items that reached this threshold were retained without modification, and sub-threshold items were flagged for revision or removal. Items with stable inter-group disagreement (IQR > 1.6 in ≥2 rounds) were subject to a pre-specified decision rule: retained if (a) median ≥3.5 and (b) at least one domain-specific subgroup achieved ≥75% internal consensus. An IQR ≤ 1.0 indicated opinion stability. All decision rules were defined before Round 1. To distinguish genuine opinion stabilization from social compliance, Kendall’s coefficient of concordance (*W*) was computed for each thematic block across rounds, using the ratings of all panelists per item as input. *W* was calculated as the classic coefficient of concordance without correction for tied ranks. For each block, statistical significance was assessed via the Friedman chi-squared approximation (*χ*^2^ = *m*·(*k* − 1)·*W*; d*f* = *k* − 1). Stabilization was operationalized as statistically significant within-block concordance (*p* < 0.05) that was maintained or increased from Round 1 to Round 3; the full per-block *W* with *χ*^2^, d*f* and exact *p*-values for all rounds is reported in Supplementary file S2.

### 2.6 External validation and ethics

Following instrument finalization, a cognitive pre-test was conducted (December 2025 to January 2026) with a convenience sample of 12 adult residents of Reinfeld and Nordstormarn who were not involved in the Delphi panel, using a structured think-aloud protocol and a post-completion feedback questionnaire administered via a survey platform. A population-based pre-test (*n* = 50) was subsequently conducted from January to March 2026 via multichannel recruitment (QR codes, municipal, and BOS networks). The study was approved by the Ethics Committee of the Universität zu Lübeck (reference: AZ 2026-127) and was conducted in accordance with the Declaration of Helsinki and GDPR (Art. 6 Abs. 1 lit. e; Art. 9 Abs. 2 lit. j DSGVO). All the participants provided informed consent.

The preparation of the manuscript did not involve AI support. All analytical content, data interpretation, and conclusions were produced and verified by the authors.

## 3 Results

### 3.1 Expert panel

The panel comprised 26 individuals from eight professional domains (Table 1). Emergency aid organizations represented the largest domain (*n* = 5, 19.2%), followed by science/academia, fire brigade/BOS, and German Armed Forces (Bundeswehr) (*n* = 4 each, 15.4%). The panel also included municipal administration and civil protection, and church/civil society (*n* = 3 each, 11.5%), technical relief (*n* = 2, 7.7%), and communications/PR (*n* = 1, 3.8%). Eighteen panelists were male (69.2%); 61.5% reported >15 years of relevant professional experience; 46.2% held a university degree; and 53.8% were currently operationally active in a BOS organization. Within the science/academic domain, three panelists were physicians and one was a paramedic (Notfallsanitäter) holding a degree in emergency-medical pedagogy. In addition, one of the four Fire Brigade/BOS panelists held an active EMS qualification (Rettungssanitäter/Notfallsanitäter). Emergency-medicine and emergency medical services (EMS) expertise was therefore represented in both the academic and the operational BOS subgroups. Response rates were 100% in Rounds 1 and 2 and 92.3% in Round 3 (24/26; two panelists from the Aid Organizations domain withdrew due to scheduling conflicts, having previously confirmed acceptance of their Round 2 positions in writing). Fully anonymized panel characteristics, including professional domain, years of experience, and gender distribution, are provided in Supplementary file S4.

**Table 1:** Expert panel characteristics by professional domain (*n* = 26).

Domain	*n*	% Panel	R1 (*n*/*n*)	R2 (*n*/*n*)	R3 (*n*/*n*)
Science/Academic	4	15.4	4/4	4/4	4/4
Fire brigade/BOS	4	15.4	4/4	4/4	4/4
Technical relief/THW	2	7.7	2/2	2/2	2/2
Emergency aid organizations	5	19.2	5/5	5/5	3/5
German armed forces (Bundeswehr)	4	15.4	4/4	4/4	4/4
Church/Civil society	3	11.5	3/3	3/3	3/3
Municipal admin./Civil prot.	3	11.5	3/3	3/3	3/3
Communications/PR	1	3.8	1/1	1/1	1/1
Total	26	100	26/26	26/26	24/26

BOS, Behörden und Organisationen mit Sicherheitsaufgaben. Response rates: R1-R2: 26/26 (100%); R3: 24/26 (92.3%). Two panelists (emergency aid organizations) withdrew before Round 3; written confirmation of Round 2 positions was accepted as final.

### 3.2 Item flow across rounds

#### 3.2.1 Round 1 (October 2025)

All 26 panelists participated in the study (100% response rate). The initial pool of 183 items across 14 blocks yielded immediate consensus (≥75% rating ≥4) for 86 items (47.0% of the total). Thirty-eight items (20.8%) were retained in modified form; 59 (32.2%) were deleted; and 13 new items (7.1%) were added to address gaps identified by the panelists (e.g., Cell Broadcast awareness, balcony solar installations, and Bundeswehr reserve familiarity). Thus, Round 2 comprised 137 items. The mean block-level consensus in Round 1 was 78.9% (range: 70.3–83.2%), with seven items displaying IQR > 1.6.

#### 3.2.2 Round 2 (late October 2025)

All 26 panelists participated. The dual-rating design (relevance + comprehensibility) enabled the targeted reformulation of 28 items. Consensus was not reached on four items after the second rating: two (item 10.9, Block 10; item 11.5, Block 11) were retained under the pre-specified stable-disagreement rule, one (Block 3) was reformulated and reached consensus within Round 2, and the remaining item was deleted. Together with 33 further sub-threshold items, 34 items were removed in total, reducing the pool to 103 closed-response content items (with conditional free-text and consent questions, 132 administered questions) entering the confirmatory Round 3. The mean consensus increased to 85.3% (+6.4 pp), and the mean IQR decreased to 1.12.

#### 3.2.3 Round 3 (November 2025)

Twenty-four of the 26 panelists participated (92.3%). Round 3 was a confirmatory review: no items were deleted, and minor editorial refinements were applied in three cases. No panelist identified any substantive domain omissions. Table 2 presents the item flow by block, and Table 3 presents the block-level consensus rates and CVI values across all three rounds. Item-level consensus data for all 183 initial items, including median ratings, IQR, and round-by-round decision outcomes, are provided in Supplementary file S2.

**Table 2:** Item flow across the three Delphi rounds by thematic block.

Blk	Thematic domain	R1 In	R1 Ret.	R1 Mod.	R1 Del.	R1 +New	R2 In	R2 Mod.	R2 Del.	Final (*n*)
1	Sociodemographic	15	11	3	1	1	15	2	1	14
2	Personal risk appraisal	13	6	2	5	1	9	2	2	7
3	Threat perception and warning	16	6	4	6	2	12	3	3	9
4	Emergency access and shelter	12	6	2	4	1	9	1	2	7
5	First aid competencies	14	6	3	5	1	10	2	2	8
6	Household preparedness	19	10	4	5	2	16	3	3	13
7	Social resilience	13	6	3	4	0	9	2	2	7
8	Crisis communication	12	6	2	4	1	9	2	4	5
9	Governmental expectations	14	7	3	4	1	11	2	3	8
10	Societal stability	13	6	3	4	0	9	2	1	8
11	Civil-military cooperation	10	3	2	5	1	6	2	3	3
12	Volunteer engagement	13	5	3	5	0	8	2	2	6
13	Preparedness motivation	11	5	2	4	1	8	2	5	3
14	Post-survey reflection	8	3	2	3	1	6	1	1	5
Σ	All blocks	183	86	38	59	13	137	28	34	103

*Blocks 10 and 11 each retained one item with stable expert disagreement through the confirmatory Round 3 (items 10.9 and 11.5); no items were deleted in Round 3 (see Section 3.3 and Supplementary file S5). R1 In, initial literature-generated pool of items; Ret., retained without modification; Mod., items modified; Del., items deleted; +New, new items added following the panelist proposals; Final (*n*), closed-response content items in the validated instrument (the full administered instrument additionally comprises conditional free-text follow-up and consent questions; 132 questions in total).

**Table 3:** Block-level consensus rates (%) across the three Delphi rounds and Content Validity Index (CVI) in round 3.

Blk	Thematic domain	R1 Cons. (%)	R1 IQR	R2 Cons. (%)	R2 IQR	R3 Cons. (%)	R3 IQR	CVI (R3)
1	Sociodemographic	83.2	1.2	89.4	0.8	94.1	0.6	0.94
2	Personal risk appraisal	78.5	1.5	85.7	1.1	91.3	0.7	0.91
3	Threat perception and warning	74.8	1.8	83.2	1.3	89.6	0.9	0.89
4	Emergency access and shelter	80.1	1.4	87.5	0.9	93.2	0.6	0.93
5	First aid competencies	77.3	1.6	84.9	1.2	90.8	0.8	0.91
6	Household preparedness	81.4	1.3	88.2	0.9	93.7	0.6	0.93
7	Social resilience	76.9	1.7	84.3	1.2	90.5	0.8	0.9
8	Crisis communication	79.8	1.4	86.6	1	92.1	0.7	0.92
9	Governmental expectations	75.4	1.9	83.7	1.3	89.2	0.9	0.89
10	Societal stability	72.1	2.1	80.5	1.5	86.8	1.1	0.87
11	Civil-military cooperation†	70.3	2.3	79.8	1.6	85.4	1.2	0.85
12	Volunteer engagement	78.2	1.5	85.4	1.1	91	0.8	0.91
13	Preparedness motivation	80.7	1.3	87.9	0.9	93.4	0.6	0.93
14	Post-survey reflection	82.4	1.2	89.1	0.8	94.3	0.5	0.94
∅	Mean (all blocks)	78.9	—	85.3	—	91.2	—	0.91

^†^Blocks 10 and 11 each retained one item with stable expert disagreement in Round 3 (items 10.9 and 11.5; IQR 1.30 each; retained per the pre-specified decision rule; see Section 3.3 and Supplementary file S5). Consensus: ≥75% of panelists rated ≥4/5 (a priori threshold). IQR, mean interquartile range across block items (scale 1–5; ≤1.0 = stable); CVI, proportion rating ≥4/5, computed at the block level. All 14 blocks had a CVI ≥ 0.80. *n* = 26 (R1-R2); *n* = 24 (R3).

### 3.3 Bipolarity analysis and stable disagreement

The consensus rates monotonically increased across all 14 blocks from Round 1 to Round 3 (Table 3). Kendall’s *W* was statistically significant across all 14 blocks in both Round 1 (*W* range 0.16–0.32; all *p* < 0.001) and Round 3 (*W* range 0.20–0.41; all *p* ≤ 0.001; Block 13 *p* = 0.001), confirming that the panelists agreed on the relative ranking of items within each block beyond chance in all rounds. *W* increased from Round 1 to Round 3 in 11 of 14 blocks, with the largest gains observed in Blocks 4 (Emergency access and shelter; Δ*W* = +0.13), 10 (Societal stability; Δ*W* = +0.12), and 2 (Personal risk appraisal; Δ*W* = +0.12). Three blocks (8, 12, 13) showed marginal decreases in *W* alongside rising consensus rates, reflecting increasing within-block item differentiation as more panelists converged on high-relevance ratings in advanced consensus stages, a pattern consistent with the published Delphi methodology literature. Taken together, the monotonic IQR convergence from 1.60 (Round 1) to 0.77 (Round 3) and the statistically significant rank-order agreement across all rounds support the interpretation that progressive consensus reflected genuine expert opinion formation rather than panel attrition or social desirability effects. The complete per-block Kendall’s *W* (computed without correction for tied ranks) with the corresponding Friedman *χ*^2^, degrees of freedom and exact *p*-values for all three rounds is provided in Supplementary file S2.

Seven items exhibited IQR > 1.6 in Round 1: five stabilized to IQR ≤ 1.4 by Round 3 following targeted reformulation. Two items, item 10.9, on the acceptance of temporary state-ordered movement restrictions (Block 10), and item 11.5, on Bundeswehr deployment without an explicit civilian request (Block 11), each retained an IQR of 1.30 in Round 3 (Supplementary file S5). Bipolarity analysis confirmed two distinct opinion clusters: military and governmental panelists rated these items as highly relevant (median 4-5), whereas church-community and lay panelists rated them lower (median 2-3). Notably, Block 11 showed the highest absolute *W* in Round 3 (*W* = 0.41). This figure is the block-level coefficient of concordance reported in Supplementary file S2.2 and should not be confused with the item-level *W* values documented for the individual retained items in Supplementary file S5, which reflect a strong within-cluster rank agreement among both opposing groups despite their divergent median positions. This finding quantitatively distinguishes substantive expert disagreement from random response scattering. According to the pre-specified decision rule, both items were retained, each meeting the criteria of a median ≥3.5 and ≥75% internal consensus within the military/governmental subgroup, and were annotated in the instrument (Supplementary file S5).

### 3.4 Final instrument

The validated KOMPAKT112 instrument comprises 132 items in 14 sequentially ordered thematic blocks (closed-response content items together with conditional free-text follow-up and consent questions): Blocks 1-2 (sociodemographic profile and risk appraisal), Blocks 3-4 (hazard awareness and warning system familiarity), Blocks 5-6 (first aid competencies and household preparedness behavior, constituting the two primary endpoints of the main survey), Blocks 7-8 (social resilience and crisis communication), Blocks 9–11 (institutional trust, governmental expectations, and civil-military cooperation), Blocks 12-13 (volunteer engagement and preparedness motivation), and Block 14 (post-survey awareness reflection, a secondary endpoint). The item formats comprised five-point Likert scales (*n* = 58), single-response multiple-choice (*n* = 31), multiple-response multiple-choice (*n* = 28), ranking tasks (*n* = 9), and grid/matrix formats (*n* = 6). The instrument is provided in Supplementary file S1.

### 3.5 Cognitive pre-test

Twelve participants (December 2025 to January 2026) completed the instrument on a live platform using a structured think-aloud protocol and a post-completion feedback questionnaire administered on the live platform (both instruments are provided in Supplementary file S6). The mean comprehensibility was 1.60/4.00 (SD 0.29; scale: 1 = very good, 4 = poor); 91% of block-participant combinations were rated 1 or 2. Six blocks required revision: Block 3 (“Heulton” unfamiliar to most participants; “Cell Broadcast” required disambiguation to “(direkt aufs Handy ohne App)”); Block 5 (HLW spelled out in full); Block 6 (10-item priority ranking simplified); Block 7 (block introduction rewritten in plain language); Block 9 (definitional distinction between Amt and Gemeinde added); Block 11 (Bundeswehr response option list condensed). The mean completion time was 28.3 min, confirming alignment with the 25–30 min target range.

### 3.6 Population-based pre-test (*n* = 50)

Fifty adult residents of Reinfeld and Nordstormarn completed the instrument from January to March 2026 (100% completion rate; no technical errors). The sample reflected the target population in terms of gender (48% female, 48% male, 4% diverse), age, and educational background. Score distributions confirmed discrimination across preparedness levels and the absence of floor or ceiling effects (Table 4).

**Table 4:** Population-based pre-test (*n* = 50): subscale score distributions.

Score dimension	*n*	Mean (%)	SD (%)	Median (%)	Min (%)	Max (%)	Interpretation
Vorsorgewissen-Score (Crisis Preparedness Knowledge Score)	50	76.2	11.3	77.0	49.8	96.4	Near-normal; mean >75% threshold; SD reflects population heterogeneity
Vorsorgeverhalten-Score (Crisis Preparedness Behavior Score)	50	68.9	13.7	69.5	38.5	91.2	Greater variance than knowledge; gap Δ = 7.3 pp.; no floor effect
Gesamt-Vorsorgescore (Composite Preparedness Score)	50	72.6	10.8	73.0	44.2	93.8	Composite knowledge + behavior; satisfactory baseline; no participant <40%

Vorsorgewissen-Score, proportion of knowledge items answered correctly (0–100%); Vorsorgeverhalten-Score, proportion of preparedness behaviors enacted (0–100%); Gesamt-Vorsorgescore, arithmetic mean of both subscales; pp, percentage points.

The two subscales are block-crossing indices: knowledge- and behavior-type items drawn from several thematic blocks were each coded to a uniform 0-1 metric (item-specific, following the gradation of each item’s response options), averaged within subscale and multiplied by 100; the composite Gesamt-Vorsorgescore is the unweighted mean of both subscales. The full scoring key is provided in Supplementary file S8. The Vorsorgewissen-Score (knowledge subscale) yielded a mean of 76.2% (SD 11.3%), and the Vorsorgeverhalten-Score (behavior subscale) yielded 68.9% (SD 13.7%), confirming a knowledge–behavior gap of 7.3 percentage points. The composite Gesamt-Vorsorgescore averaged 72.6% (SD 10.8%); no participant scored below 40%. Selected item frequencies aligned with national benchmarks: 34% reported ≥7-day food stockpiles (BBK 2022 benchmark: ~29%); 44% had installed the NINA warning app (Eurobarometer 2024 Germany: ~38%); 18% knew all siren signals (~20% nationally); and 40% had completed a first aid course within 5 years (BBK benchmark: ~48%). 70% of participants reported increased awareness of crisis preparedness after completing the survey. Given the non-representative convenience sample (*n* = 50), these results, including the knowledge–behavior gap and any sociodemographic patterns, are interpreted as exploratory observations rather than population-level estimates. Fully anonymized score distributions and key item frequency data for all 50 pre-test participants are provided in Supplementary file S7.

### 3.7 Preliminary internal consistency (reflective subscales)

Although comprehensive psychometric characterization was reserved for the main survey, we estimated preliminary block-level internal consistency from the pre-test (*n* = 50) for those blocks that constitute reflective Likert batteries. Standardized Cronbach’s *α* (with 2,000-sample bootstrap 95% confidence intervals) was 0.92 (95% CI 0.87–0.95) for first-aid competence self-assessment (7 items), 0.86 (0.78–0.90) for the household-supply inventory (10 items), 0.83 (0.68–0.89) for institutional trust and expectations (6 items), and 0.76 and 0.77 for engagement motivators and barriers (10 items each). The remaining blocks comprise heterogeneous single-indicator knowledge and behavior items or multi-select inventories that are formative rather than reflective, for which internal-consistency coefficients are not appropriate; test–retest reliability could not be estimated because the pre-test used a single administration. Full results are reported in Supplementary file S8.

### 3.8 CREDES compliance

All 29 CREDES criteria were addressed: *a priori* consensus threshold defined before Round 1; item-level results reported per round; stable disagreement documented with explicit decision rules; panel characteristics and response rates reported transparently; external validation via cognitive pre-testing and population-based pre-test conducted; full instrument published as Supplementary material. The completed CREDES checklist is provided in Supplementary file S3.

## 4 Discussion

### 4.1 Principal findings

This study reports the development and consensus-based validation of the KOMPAKT112. To our knowledge, this is the first all-hazards, multi-dimensional population survey designed for municipal-level civil protection research in the German-speaking context. Across three rounds with 26 interdisciplinary experts, we established a 132-item, 14-block instrument, achieving a mean Round 3 consensus rate of 91.2% and an overall CVI of 0.91. Both metrics compared favorably with published benchmarks for Delphi-derived preparedness instruments. Population pre-test data confirmed score differentiation, the absence of floor/ceiling effects, and a knowledge–behavior gap of 7.3 percentage points, consistent with the international evidence base. The instrument also demonstrated a 70% sensitization effect and therefore has a well-documented secondary benefit of structured preparedness survey participation (22, 23). The questionnaire is positioned as both a measurement tool and a population-level preparedness intervention, aligned with the Deutsche Resilienzstrategie 2022 and EU 2030 preparedness targets (6).

### 4.2 Methodological contributions

The 75% consensus threshold chosen for KOMPAKT112 falls at the upper end of the range commonly reported in health-related Delphi studies (60–80%) (19, 24) and is consistent with that of the HEPI development study (12). A stricter threshold is defensible here because civil protection preparedness constitutes a comparatively well-delineated operational domain and because cross-domain endorsement is essential for a tool intended to serve practitioners from diverse professional backgrounds. The modified, literature-based first round, rather than an open-ended initial questionnaire, was selected to maintain structural equivalence with established preparedness frameworks while accommodating the panel’s interdisciplinary nature. This design has been shown to produce higher engagement and lower attrition (25, 26). Progressive IQR convergence from 1.60 (Round 1) to 0.77 (Round 3) provides quantitative evidence of genuine opinion stabilization rather than social compliance, a distinction emphasized in CREDES guidance (17) and is consistent with comparable three-round Delphi studies in disaster preparedness (26–28). The observed *W* values (0.16–0.41 across blocks and rounds) fall within the range typical for health-related Delphi instrument development (0.20–0.45), in which *W* functions as supporting evidence of inter-expert concordance rather than a primary validity criterion (26, 29).

The interdisciplinary composition of the panel was a defining methodological strength. The systematic inclusion of frontline BOS professionals, Bundeswehr personnel, church community representatives, and municipal administrators, in addition to academic experts, is unprecedented in published studies on the development of German preparedness instruments. This breadth yielded operational insights absent from the academic literature: BOS panelists proposed the Cell Broadcast item and balcony solar installations; Bundeswehr panelists shaped the civil-military cooperation block; and church representatives enriched the social resilience and neighborhood solidarity dimensions. The 100% panel response rate across the first two rounds exceeded the median reported in Delphi studies. Full instrument transparency, achieved by making all 132 items publicly available (Supplementary file S1), addressed the critical reporting gap identified in the systematic review. Most published Delphi studies omit instrument availability, incomplete consensus criteria, and attrition reporting—deficiencies that fundamentally undermine the reproducibility and validity of the derived instruments (25, 26). The overall CVI of 0.91 (range 0.85–0.94) exceeded the recommended ≥0.80 threshold (30) and was consistent with high-quality comparator instruments, including the healthcare rescuer resilience tool (S-CVI/Ave 0.97; (29)), the MEID preparedness index (I-CVI ≥ 0.80; (31)), and the PHEP indicator study (equivalent CVI 0.70–0.85; (27)).

### 4.3 Knowledge–behavior gap and sensitization effect

The 7.3 pp. knowledge–behavior gap observed in the pre-test replicates one of the most robust empirical regularities in preparedness research. The first systematic review and meta-analysis of household emergency preparedness interventions (32) underscores that knowledge provision alone is insufficient, and behavioral and structural interventions are required to bridge the gap. Psychological barriers, particularly low perceived self-efficacy and optimism bias, consistently moderate knowledge-to-behavior translation across cultural settings (33, 34). High institutional trust, when it substitutes rather than complements individual preparedness, and fragmented public risk communication further widen the gap, as documented in European and East Asian survey populations (10, 33). These constructs were directly operationalized in KOMPAKT112 Blocks 2, 6, and 13, enabling the main survey to test mediation pathways in the German municipal context for the first time. The 7.3 pp. gap is somewhat smaller than in large national surveys, which is plausible because pre-test participants recruited via project networks may have had above-average prior civil protection engagement. This underscores the importance of the urban–rural comparative hypothesis (H3) embedded in the main study design. Importantly, the operationalization of knowledge (self-reported familiarity and recognition) and behavior (self-reported enactment) in KOMPAKT112 differs from that of benchmark instruments such as the HEPI, the EPC and the FEMA National Household Survey; the absolute magnitude of the gap is therefore not directly comparable across instruments, and only the qualitative pattern of knowledge exceeding enacted behavior is transferable. The sensitization effect observed in 70% of pre-test participants is consistent with evidence from community disaster awareness programs demonstrating that structured engagement raises perceived susceptibility, severity, and self-efficacy (22, 23, 35)—mechanisms that the completion of a comprehensive preparedness questionnaire is positioned to activate.

### 4.4 The civil-military cooperation domain and wider implications

Block 11 (civil–military cooperation) was the most contested domain in the Delphi process, producing the lowest Round 1 consensus (70.3%). Together with item 10.9 (Block 10), its item 11.5 retained an IQR of 1.30 in Round 3. This stable disagreement is substantively meaningful as it reflects the historically ambiguous role of the Bundeswehr in domestic emergency operations under Germany’s post-World War II constitutional architecture (36). This ambiguity has concrete operational consequences, analogous to the civil-military coordination frictions documented in infectious-disease deployments in European settings (37). Civilian perceptions of military involvement constitute a critical yet understudied determinant of the effectiveness of civil-military cooperation in disaster settings (37–40). Survey-based evidence shows that public support for military roles in domestic crises is strongly conditional on perceived mandate clarity and non-coercive framing, patterns documented in pandemic deployments in the United States, Australia, and European hospital settings (37, 39, 40). Retaining these items with transparent annotation of expert disagreement rather than excluding them on consensus grounds positions KOMPAKT112 to generate the first population-level data on civil-military trust and role expectations in the German municipal context. This is directly relevant given the recent elevation of civil-military preparedness coordination in European security policy (41). Beyond this specific domain, the KOMPAKT112 instrument addresses a structural gap identified in the preparedness literature: the absence of standardized municipal-level measurement tools that link survey data directly to civil protection planning outputs (11, 42, 43). Its 14-block modular architecture, full public availability, and CREDES-compliant development make it directly adaptable for comparative deployment across other German Kreise, potentially supporting the first nationally comparable municipal preparedness dataset aligned with the Deutsche Resilienzstrategie 2022. To gain a deeper understanding, further considerations should be discussed. First, the politically and constitutionally sensitive items (acceptance of emergency decrees, Block 10; scope of Bundeswehr domestic operations, Block 11) are attitudinal rather than knowledge- or behavior-based; they are retained within the instrument and the composite framework rather than excluded, but are reported as a distinct attitudinal dimension so that their interpretation remains separable from preparedness knowledge and behavior. Second, to address potential effects on overall response behavior and any ideological skew, the main study will report the preparedness composite both with and without these items as a sensitivity analysis and will examine context and order effects. Third, the high within-cluster concordance (Block 11, Round 3 *W* = 0.41) indicates that the disagreement reflects two coherent value positions rather than random responding, supporting transparent retention with annotation over exclusion.

### 4.5 Strengths, limitations, and future research

#### 4.5.1 Strengths

Full CREDES compliance (29/29 criteria); *a priori* consensus definition; interdisciplinary 8-domain expert panel; transparent bipolarity analysis with pre-specified decision rules; two-stage external validation (cognitive pre-test + population pre-test); full instrument availability (Supplementary file S1); GDPR-compliant university-hosted platform.

#### 4.5.2 Limitations

The geographic specificity of panel recruitment (Schleswig-Holstein network) limits the direct generalizability of consensus findings to other German federal states or international settings; adaptation and local validation would be required for markedly different civil protection contexts. The instrument was developed and validated in German only; cross-cultural use requires translation and psychometric revalidation. Formal psychometric reliability assessment, including Cronbach’s *α* for block-level subscales, confirmatory factor analysis, and test–retest reliability, was not completed at this content-validation stage; preliminary block-level internal consistency for the reflective subscales is now reported (Section 3.7; Supplementary file S8), whereas confirmatory factor analysis, measurement invariance and test–retest reliability will follow with the main survey, as is typical in Delphi-based instrument development. The pre-test sample (*n* = 50) was powered only for feasibility and comprehensibility assessments and was statistically insufficient for exploratory, let alone confirmatory, factor analyses. Full psychometric characterization will be reported as a second-phase study using the main survey dataset (*n* ≥ 800), which is adequately powered. The pre-test sample (*n* = 50, convenience) may have introduced a selection bias toward individuals with above-average civil protection engagement; therefore, scores should be interpreted as indicative rather than representative. Finally, the panel’s gender imbalance (69.2% male) reflects occupational sector distributions within emergency management but warrants acknowledgment regarding the representation of female professional perspectives in the consensus process. Several stakeholder groups were comparatively under-represented (communications/PR *n* = 1, technical relief *n* = 2, municipal administration *n* = 3), and recruitment proceeded from the project’s stakeholder network with snowball extension; both factors may have shaped item generation and consensus formation, for instance by favoring operational over health-sector or risk-communication perspectives. Relatedly, the seven source frameworks did not include the WHO Emergency and Disaster Risk Management for Health (EDRM) Framework or the International Health Regulations (44–46), which address municipal-level preparedness, civil–military coordination and multi-sectoral response; their omission may have limited explicit health-sector coverage and should be addressed in future adaptations. Finally, recruitment of the planned main sample (*n* ≥ 800) for a 25–30 min instrument is demanding; feasibility is supported by multichannel municipal and BOS recruitment combining paper-based and online modes, which in the present project proved essential for reaching less digitally engaged residents, although a conservative response rate should be anticipated.

#### 4.5.3 Future research

The main cross-sectional survey (target *n* ≥ 800) conducted in 2026 will test six pre-specified hypotheses, including urban–rural comparisons and predictors of engagement readiness. A subsequent psychometric validation study (internal consistency, construct and criterion validity, and test–retest reliability) will further establish the instrument’s construct validity and reliability. Longitudinal re-administration will enable monitoring of preparedness trends and evaluation of the effects of civil protection campaigns. Multi-site deployment across other German districts and regions would produce the first nationally comparative municipal preparedness datasets.

## 5 Conclusion

The KOMPAKT112 Delphi study produced a methodologically rigorous, content-valid instrument for the empirical assessment of crisis preparedness at the municipal population level. Its development through an interdisciplinary, CREDES-compliant, three-round Delphi process with full consensus transparency establishes a rigorous and transparent methodological reference point for preparedness instrument development in the German context. A knowledge–behavior gap and a 70% sensitization effect confirmed in the pre-test highlight both the measurement relevance and the practical public health value of population-level preparedness surveys. We invite municipalities, research groups, and civil protection authorities to use, adapt, and build upon this instrument to generate the comparative, longitudinal, sub-national evidence that German and European civil protection policy urgently needs.

## Data Availability

The raw data supporting the conclusions of this article will be made available by the authors, without undue reservation.
