# Supplementary material for: Development and consensus-based validation of a comprehensive population survey instrument for municipal crisis preparedness: a three-round Delphi study (KOMPAKT112)
Source: Front Public Health. 2026 Aug 5;14:1883344. doi: 10.3389/fpubh.2026.1883344 (PMC13485894; doi:10.3389/fpubh.2026.1883344)
Supplement: Supplementary file 1 [file Supplementary_file_1.pdf]

KOMPaKT112 -

„Vorsorge und Resilienz im Bevölkerungsschutz – eine empirische Erhebung auf kommunaler Ebene“

In dieser Umfrage sind 132 Fragen enthalten.

Vorsorge und Resilienz im Bevölkerungsschutz - eine empirische Erhebung auf kommunaler Ebene

Herzlich willkommen und vielen Dank, dass Sie sich die Zeit für diesen wichtigen Fragebogen nehmen. Ziel dieser Erhebung ist es, das Maß der Vorsorge und die Resilienz in der Bevölkerung zu erfassen.

Ihre ehrlichen Antworten dienen als wichtige Arbeitsgrundlage für Kommunen, um den Bevölkerungsschutz besser an die Bedürfnisse der Menschen anzupassen.

Die Befragung dauert etwa 25-30 Minuten. Alle Angaben werden

anonym behandelt und unterliegen den Datenschutzbestimmungen (DSGVO). Es gibt keine richtigen oder falschen Antworten – uns interessiert Ihre persönliche Einschätzung.

Bei Rückfragen können Sie sich jederzeit an PD Dr. med. Klaas Franzen unter der Telefonnummer 0451/ 50045003 oder klaas.franzen@uni-luebeck.de wenden.

Bitte geben Sie an, ob Sie die Informationen verstanden haben und an der Umfrage teilnehmen möchten. *

Bitte wählen Sie eine der folgenden Antworten:
Bitte wählen Sie nur eine der folgenden Antworten aus:

◯ Ich habe die Informationen verstanden und möchte an der Umfrage teilnehmen.

1. DEMOGRAPHISCHE ANGABEN

In welcher Gemeinde/Stadt wohnen Sie? *

Bitte wählen Sie eine der folgenden Antworten:
Bitte wählen Sie nur eine der folgenden Antworten aus:

- ◯ Reinfeld (Holstein)
- ◯ Gemeinde in Nordstormarn
- ◯ Außerhalb von Nordstormarn
- ◯ Sonstiges

In welcher Gemeinde/Stadt wohnen Sie?

Beantworten Sie diese Frage nur, wenn folgende Bedingungen erfüllt sind:
Antwort war 'Sonstiges' bei Frage ' [Q00]' (In welcher Gemeinde/Stadt wohnen Sie?)

Bitte geben Sie Ihre Antwort hier ein:

Wie würden Sie Ihren Wohnort charakterisieren? *

Bitte wählen Sie eine der folgenden Antworten:
Bitte wählen Sie nur eine der folgenden Antworten aus:

- ◯ Dorfrand
- ◯ Dorfzentrum
- ◯ Stadtrand
- ◯ Stadtzentrum

Wie alt sind Sie? *

Bitte wählen Sie eine der folgenden Antworten:
Bitte wählen Sie nur eine der folgenden Antworten aus:

- ◯ unter 18 Jahre
- ◯ 19-29 Jahre
- ◯ 30-39 Jahre
- ◯ 40-49 Jahre
- ◯ 50-59 Jahre
- ◯ 60-69 Jahre
- ◯ 70 Jahre oder älter

Welchem Geschlecht fühlen Sie sich zugehörig? *

Bitte wählen Sie eine der folgenden Antworten:
Bitte wählen Sie nur eine der folgenden Antworten aus:

- ◯ Männlich
- ◯ Weiblich
- ◯ Divers

Wie viele Personen leben in Ihrem Haushalt (Sie selbst eingeschlossen)? *

Bitte wählen Sie eine der folgenden Antworten:
Bitte wählen Sie nur eine der folgenden Antworten aus:

- ◯ 1 Person (ich lebe allein)
- ◯ 2 Personen
- ◯ 3 Personen
- ◯ 4 Personen
- ◯ 5 oder mehr Personen

Leben in Ihrem Haushalt Kinder unter 18 Jahren? *

Bitte wählen Sie eine der folgenden Antworten:
Bitte wählen Sie nur eine der folgenden Antworten aus:

- ◯ Ja
- ◯ Nein

Leben in Ihrem Haushalt Personen mit besonderen Bedürfnissen? *

Bitte wählen Sie eine der folgenden Antworten:
Bitte wählen Sie nur eine der folgenden Antworten aus:

- ◯ Ja
- ◯ Nein
- ◯ Möchte ich nicht angeben

(z.B. ältere/pflegebedürftige Menschen, Menschen mit Behinderungen, chronisch Kranke)

Welchen höchsten Bildungsabschluss haben Sie? *

Bitte wählen Sie eine der folgenden Antworten:
Bitte wählen Sie nur eine der folgenden Antworten aus:

- ◯ Noch in schulischer Ausbildung
- ◯ Kein Abschluss
- ◯ Hauptschulabschluss
- ◯ Mittlere Reife/ Realschulabschluss
- ◯ Fachhochschulreife/ Allgemeine Hochschulreife
- ◯ Abgeschlossene Berufsausbildung
- ◯ Fachhochschul-/ Hochschulabschluss
- ◯ Promotion

Sind Sie derzeit erwerbstätig? *

Bitte wählen Sie eine der folgenden Antworten:
Bitte wählen Sie nur eine der folgenden Antworten aus:

- ◯ Ja, in Vollzeit
- ◯ Ja, in Teilzeit
- ◯ Nein, in Ausbildung/ Studium
- ◯ Nein, arbeitslos/ arbeitssuchend
- ◯ Nein, im Ruhestand
- ◯ Nein, aus anderen Gründen

Sind Sie ehrenamtlich engagiert? *

Wählen Sie alle zutreffenden Optionen
Bitte wählen Sie alle zutreffenden Antworten aus:

- ☐ Ja, bei der Freiwilligen Feuerwehr
- ☐ Ja, beim Technischen Hilfswerk (THW)
- ☐ Ja, bei Rettungsdiensten (z.B. DRK, Johanniter, Malteser, ASB)
- ☐ Ja, in anderen Hilfsorganisationen
- ☐ Ja, in Vereinen (z.B. Sport, Kultur)
- ☐ Ja, in der Kirchengemeinde
- ☐ Ja, in anderen Bereichen
- ☐ Nein, ich bin nicht ehrenamtlich engagiert

Haben Sie eine berufliche oder ehrenamtliche Verbindung zu Behörden und Organisationen mit Sicherheitsaufgaben (BOS)? *

Bitte wählen Sie eine der folgenden Antworten:

Bitte wählen Sie nur eine der folgenden Antworten aus:

- ◯ Ja, beruflich
- ◯ Ja, ehrenamtlich
- ◯ Ja, beides
- ◯ Früher, aber nicht mehr
- ◯ Nein

Haben Sie eine Verbindung zur Bundeswehr? *

Wählen Sie alle zutreffenden Optionen

Bitte wählen Sie alle zutreffenden Antworten aus:

- ☐ Ja, als aktive/r Soldat/in
- ☐ Ja, als Reservist/in
- ☐ Ja, als Zivilangestellte/r
- ☐ Ja, durch Familienangehörige
- ☐ Früher, jetzt aber nicht mehr
- ☐ Nein

Ich wohne in einem... *

Bitte wählen Sie eine der folgenden Antworten:
Bitte wählen Sie nur eine der folgenden Antworten aus:

- ◯ Eigenen Haus
- ◯ Eigener Wohnung (Eigentum)
- ◯ Mietwohnung
- ◯ Zur Miete in einem Haus
- ◯ Sonstige Wohnform

Haben Sie Zugang zu einem Garten oder Außenfläche? *

Bitte wählen Sie eine der folgenden Antworten:
Bitte wählen Sie nur eine der folgenden Antworten aus:

- ◯ Ja, eigener Garten
- ◯ Ja, Gemeinschaftsgarten/ Schrebergarten
- ◯ Ja, Balkon/ Terrasse
- ◯ Nein, keine Außenfläche

Begriffsdefinition

Im folgenden Fragebogen werden Begriffe verwendet, welche an dieser Stelle kurz definiert werden. Bitte lesen Sie sich diese Definitionen aufmerksam durch.

Resilienz: Die Fähigkeit schwierige Krisen ohne dauerhafte Beeinträchtigung zu überstehen.

Krisen/ Katastrophen bezieht sich auf:
Naturkatastrophen (z.B. Erdbeben, Überschwemmungen, Stürme oder Waldbrände)
Technisches Versagen (z.B. Großbrände, Chemieunfälle oder langanhaltende Stromausfälle (Blackouts)
und Menschliches Handeln (z.B. Kriege, Terroranschläge oder schwere Unglücke)

2. PERSÖNLICHE EINSCHÄTZUNG ZUR KRISENVORSORGE

Wie stark haben Sie sich bisher persönlich mit dem Thema Katastrophenvorsorge beschäftigt? *

Bitte wählen Sie eine der folgenden Antworten:
Bitte wählen Sie nur eine der folgenden Antworten aus:

- ◯ Sehr stark - ich habe mich intensiv damit auseinandergesetzt
- ◯ Stark - ich habe mich mehrfach damit beschäftigt
- ◯ Mittelmäßig - ich habe mich gelegentlich damit beschäftigt
- ◯ Wenig - ich habe nur oberflächlich davon gehört
- ◯ Gar nicht - das Thema war für mich bisher nicht relevant

Wie wichtig finden Sie das Thema Krisenvorsorge für sich persönlich? *

Bitte wählen Sie eine der folgenden Antworten:
Bitte wählen Sie nur eine der folgenden Antworten aus:

- ○ Sehr wichtig
- ○ Wichtig
- ○ Teilweise wichtig
- ○ Weniger wichtig
- ○ Unwichtig

Welche der folgenden Krisensituationen halten Sie in Ihrer Region für realistisch möglich? *

Wählen Sie alle zutreffenden Optionen
Bitte wählen Sie alle zutreffenden Antworten aus:

- ☐ Längerer Stromausfall (Blackout)
- ☐ Extremwetterereignisse (Sturm, Starkregen, Hochwasser)
- ☐ Wintereinbruch mit Verkehrschaos
- ☐ Trinkwasserausfall
- ☐ Pandemie/Seuchenausbruch
- ☐ Großbrand/Explosion
- ☐ Chemieunfall/Schadstoffaustritt
- ☐ Terroranschlag
- ☐ Versorgungsengpässe (Lebensmittel, Medikamente)
- ☐ Sonstige
- ☐ Keine dieser Situationen halte ich für realistisch

(Mehrfachnennungen möglich)

Welche sonstigen Krisensituationen halten Sie in Ihrer Region für realistisch möglich?

Beantworten Sie diese Frage nur, wenn folgende Bedingungen erfüllt sind:
Antwort war bei Frage ' [Q016]' (Welche der folgenden Krisensituationen halten Sie in Ihrer Region für realistisch möglich?)

Bitte geben Sie Ihre Antwort hier ein:

(Mehrfachnennungen möglich)

Haben Sie bereits selbst eine Krisensituation erlebt, die Sie persönlich betroffen hat? *

Bitte wählen Sie eine der folgenden Antworten:
Bitte wählen Sie nur eine der folgenden Antworten aus:

- ◯ Ja
- ◯ Nein
- ◯ Möchte ich nicht angeben

Welche Krisensituation haben Sie schon erlebt?

Beantworten Sie diese Frage nur, wenn folgende Bedingungen erfüllt sind:
Antwort war 'Ja' bei Frage ' [Q017]' (Haben Sie bereits selbst eine Krisensituation erlebt, die Sie persönlich betroffen hat?)

Bitte geben Sie Ihre Antwort hier ein:

Woher beziehen Sie hauptsächlich Informationen zum Thema Krisenvorsorge? *

Wählen Sie alle zutreffenden Optionen

Bitte wählen Sie alle zutreffenden Antworten aus:

- [ ] Fernsehen/Rundfunk
- [ ] Zeitungen/Printmedien
- [ ] Soziale Medien (Facebook, Instagram, X/Twitter, etc.)
- [ ] Websites von Behörden (z.B. BBK, Innenministerium)
- [ ] Warn-Apps (z.B. NINA, KATWARN, WarnWetter)
- [ ] Familie, Freunde, Bekannte
- [ ] Feuerwehr/Rettungsdienste
- [ ] Gemeinde/Stadt
- [ ] Vereine/Organisationen
- [ ] Arbeitgeber
- [ ] Habe mich noch nicht informiert
- [ ] Sonstige

Aus welchen weiteren Quellen beziehen Sie hauptsächlich Informationen zum Thema Krisenvorsorge?

Beantworten Sie diese Frage nur, wenn folgende Bedingungen erfüllt sind:
Antwort war bei Frage ' [Q018]' (Woher beziehen Sie hauptsächlich Informationen zum Thema Krisenvorsorge?)

Bitte geben Sie Ihre Antwort hier ein:

Wie gut fühlen Sie sich über Ihre eigenen Möglichkeiten informiert, sich auf Krisen vorzubereiten? *

Bitte wählen Sie eine der folgenden Antworten:
Bitte wählen Sie nur eine der folgenden Antworten aus:

- ◯ Sehr gut informiert
- ◯ Gut informiert
- ◯ Mittelmäßig informiert
- ◯ Schlecht informiert
- ◯ Gar nicht informiert

Welche Informationen würden Sie sich zum Thema Krisenvorsorge wünschen? *

Wählen Sie alle zutreffenden Optionen

Bitte wählen Sie alle zutreffenden Antworten aus:

- ☐ Konkrete Checklisten
- ☐ Praktische Anleitungen
- ☐ Informationen über lokale Anlaufpunkte
- ☐ Beispiele aus anderen Regionen
- ☐ Informationsveranstaltungen
- ☐ Online-Kurse/Videos
- ☐ Keine weiteren Informationen nötig
- ☐ Sonstige

(Mehrfachnennungen möglich)

Welche sonstigen Informationen würden Sie sich zum Thema Krisenvorsorge wünschen?

Beantworten Sie diese Frage nur, wenn folgende Bedingungen erfüllt sind:
Antwort war bei Frage ' [Q020]' (Welche Informationen würden Sie sich zum Thema Krisenvorsorge wünschen?)

Bitte geben Sie Ihre Antwort hier ein:

3. WAHRNEHMUNG VON BEDROHUNGSLAGEN

Über welche Kanäle beziehen Sie Ihre Nachrichten? *

Wählen Sie alle zutreffenden Optionen
Bitte wählen Sie alle zutreffenden Antworten aus:

- ☐ Radio
- ☐ Fernsehen
- ☐ Zeitung
- ☐ Soziale Medien
- ☐ Internet (außerhalb sozialer Medien)
- ☐ Ich beziehe keine Nachrichten
- ☐ Sonstige

Über welche sonstigen Kanäle beziehen Sie Ihre Nachrichten?

Beantworten Sie diese Frage nur, wenn folgende Bedingungen erfüllt sind:
Antwort war bei Frage ' [Q021]' (Über welche Kanäle beziehen Sie Ihre Nachrichten?)

Bitte geben Sie Ihre Antwort hier ein:

In welcher Frequenz beziehen Sie die Nachrichten? *

Beantworten Sie diese Frage nur, wenn folgende Bedingungen erfüllt sind:
Antwort war NICHT bei Frage ' [Q021]' (Über welche Kanäle beziehen Sie Ihre Nachrichten?)

Bitte wählen Sie eine der folgenden Antworten:
Bitte wählen Sie nur eine der folgenden Antworten aus:

- ◯ Mehrmals täglich
- ◯ Täglich
- ◯ Mehrmals pro Woche
- ◯ Wöchentlich
- ◯ Weniger häufig als wöchentlich

Wie gut fühlen Sie sich über mögliche Bedrohungslagen in Ihrer Region informiert? *

Bitte wählen Sie eine der folgenden Antworten:
Bitte wählen Sie nur eine der folgenden Antworten aus:

- ◯ Sehr gut informiert
- ◯ Gut informiert
- ◯ Mittelmäßig informiert
- ◯ Schlecht informiert
- ◯ Gar nicht informiert

Woher würden Sie im Ernstfall erwarten, Informationen über eine Bedrohungslage zu erhalten? *

Wählen Sie alle zutreffenden Optionen

Bitte wählen Sie alle zutreffenden Antworten aus:

- [ ] Radio/Fernsehen
- [ ] Warn-Apps auf dem Smartphone
- [ ] Sirenen
- [ ] Lautsprecherdurchsagen
- [ ] Soziale Medien
- [ ] Lokale Nachrichtenseiten/Zeitungen
- [ ] Gemeinde/Stadt (Website, Aushänge)
- [ ] Feuerwehr/Polizei vor Ort
- [ ] Nachbarn/Mundpropaganda
- [ ] SMS/Cell Broadcast
- [ ] Weiß ich nicht
- [ ] Sonstige

Woher würden Sie zusätzlich im Ernstfall erwarten, Informationen über eine Bedrohungslage zu erhalten?

Beantworten Sie diese Frage nur, wenn folgende Bedingungen erfüllt sind:
Antwort war bei Frage ' [Q022]' (Woher würden Sie im Ernstfall erwarten, Informationen über eine Bedrohungslage zu erhalten?)

Bitte geben Sie Ihre Antwort hier ein:

Kennen Sie die Bedeutung verschiedener Sirenensignale? *

Bitte wählen Sie eine der folgenden Antworten:
Bitte wählen Sie nur eine der folgenden Antworten aus:

- ◯ Ja, ich kenne alle Signale und ihre Bedeutung
- ◯ Ja, ich kenne einige Signale
- ◯ Nein, aber ich weiß, dass es unterschiedliche Signale gibt
- ◯ Nein, ich kenne keine Sirenensignale
- ◯ Wir haben keine Sirenen in meinem Ort

Wissen Sie, was der Begriff "Heulton" bei Sirenen bedeutet? *

Bitte wählen Sie eine der folgenden Antworten:
Bitte wählen Sie nur eine der folgenden Antworten aus:

- ◯ Ja, ich weiß genau, was das bedeutet
- ◯ Ich habe eine ungefähre Vorstellung
- ◯ Nein, das weiß ich nicht

Wie würden Sie reagieren, wenn Sie eine Sirene hören würden? *

Wählen Sie alle zutreffenden Optionen
Bitte wählen Sie alle zutreffenden Antworten aus:

- ☐ Ich würde Radio/Fernsehen einschalten
- ☐ Ich würde eine Warn-App öffnen
- ☐ Ich würde im Internet nach Informationen suchen
- ☐ Ich würde bei der Feuerwehr/Polizei anrufen
- ☐ Ich würde meine Nachbarn fragen
- ☐ Ich würde nichts Besonderes tun
- ☐ Weiß ich nicht
- ☐ Sonstiges

Wie würden Sie außerdem reagieren, wenn Sie eine Sirene hören würden?

Beantworten Sie diese Frage nur, wenn folgende Bedingungen erfüllt sind:
Antwort war bei Frage ' [Q025]' (Wie würden Sie reagieren, wenn Sie eine Sirene hören würden?)

Bitte geben Sie Ihre Antwort hier ein:

Vertrauen Sie darauf, dass Sie im Ernstfall rechtzeitig gewarnt werden? *

Bitte wählen Sie eine der folgenden Antworten:
Bitte wählen Sie nur eine der folgenden Antworten aus:

- ◯ Ja, voll und ganz
- ◯ Eher ja
- ◯ Teils/ teils
- ◯ Eher nein
- ◯ Nein, überhaupt nicht

Haben Sie sich bereits über Warnsysteme in Ihrer Gemeinde informiert? *

Bitte wählen Sie eine der folgenden Antworten:
Bitte wählen Sie nur eine der folgenden Antworten aus:

- ◯ Ja, ausführlich
- ◯ Ja, teilweise
- ◯ Nein, aber ich hätte Interesse
- ◯ Nein, und ich sehe keinen Bedarf

Kennen Sie das Warnsystem "Cell Broadcast" (Warnmeldungen direkt aufs Handy ohne App)? *

Bitte wählen Sie eine der folgenden Antworten:
Bitte wählen Sie nur eine der folgenden Antworten aus:

- ◯ Ja, und ich weiß, wie es funktioniert
- ◯ Ja, ich habe davon gehört
- ◯ Nein, kenne ich nicht

Wie wichtig wären Ihnen regelmäßige Probealarms/Sirenentest, um die Warnsysteme kennenzulernen? *

Bitte wählen Sie eine der folgenden Antworten:

Bitte wählen Sie nur eine der folgenden Antworten aus:

- ◯ Sehr wichtig
- ◯ Wichtig
- ◯ Mittelmäßig wichtig
- ◯ Weniger wichtig
- ◯ Unwichtig

4. NOTRUF UND HILFSZUGANG

Welche der folgenden Notrufnummern kennen Sie? *

Wählen Sie alle zutreffenden Optionen

Bitte wählen Sie alle zutreffenden Antworten aus:

- ☐ 110 (Polizei)
- ☐ 112 (Feuerwehr/Rettungsdienst)
- ☐ 116117 (Ärztlicher Bereitschaftsdienst)
- ☐ Keine davon

Wann würden Sie die 112 anrufen? *

Wählen Sie alle zutreffenden Optionen
Bitte wählen Sie alle zutreffenden Antworten aus:

- ☐ Bin mir nicht sicher
- ☐ Bei lebensbedrohlichen medizinischen Notfällen
- ☐ Bei Feuer/Brand
- ☐ Bei Verkehrsunfällen mit Verletzten
- ☐ Bei starken Schmerzen (aber nicht lebensbedrohlich)
- ☐ Bei Erkältung/Fieber außerhalb der Sprechzeiten
- ☐ Bei Gas- oder Chemieunfällen

Kennen Sie alternative Kontaktmöglichkeiten für nicht lebensbedrohliche medizinische Anliegen außerhalb der Sprechzeiten? *

Bitte wählen Sie eine der folgenden Antworten:
Bitte wählen Sie nur eine der folgenden Antworten aus:

- ◯ Ja, ich kenne mehrere Möglichkeiten
- ◯ Ja, ich kenne eine Möglichkeit
- ◯ Nein, aber ich würde im Internet suchen
- ◯ Nein, ich würde immer die 112 anrufen

Im Krisenfall (z.B. Stromausfall) könnten Telefone nicht funktionieren. Wissen Sie, wo in Ihrer Nähe ein Anlaufpunkt für Notfälle sein könnte? *

Bitte wählen Sie eine der folgenden Antworten:

Bitte wählen Sie nur eine der folgenden Antworten aus:

- ◯ Nein, ich weiß nicht, wo ich hingehen sollte
- ◯ Ich hätte eine Vermutung
- ◯ Ja, ich weiß genau, wo ich hingehen würde

Welche Orte würden Sie im Krisenfall als Anlaufpunkte erwarten oder aufsuchen? *

Wählen Sie alle zutreffenden Optionen

Bitte wählen Sie alle zutreffenden Antworten aus:

- ☐ Feuerwehrgerätehaus
- ☐ Rathaus/Amtsverwaltung
- ☐ Schule
- ☐ Gemeindezentrum/Bürgerhaus
- ☐ Kirche
- ☐ Sportplatz/-halle
- ☐ Polizeistation
- ☐ Arztpraxis
- ☐ Krankenhaus
- ☐ Weiß ich nicht
- ☐ Sonstiges

Welche weiteren Orte würden Sie im Krisenfall als Anlaufpunkte erwarten oder aufsuchen?

Beantworten Sie diese Frage nur, wenn folgende Bedingungen erfüllt sind:
Antwort war bei Frage ' [Q045]' (Welche Orte würden Sie im Krisenfall als Anlaufpunkte erwarten oder aufsuchen?)

Bitte geben Sie Ihre Antwort hier ein:

Sollten solche Anlaufpunkte in Ihrer Gemeinde besser gekennzeichnet werden? *

Bitte wählen Sie eine der folgenden Antworten:
Bitte wählen Sie nur eine der folgenden Antworten aus:

- ◯ Nein, ist nicht notwendig
- ◯ Ja, wäre hilfreich
- ◯ Ja, unbedingt
- ◯ Weiß ich nicht

Welche Funktionen sollten solche Anlaufpunkte im Krisenfall erfüllen? *

Wählen Sie alle zutreffenden Optionen
Bitte wählen Sie alle zutreffenden Antworten aus:

- ☐ Informationen über die aktuelle Lage
- ☐ Ausgabe von Trinkwasser
- ☐ Möglichkeit zum Aufwärmen
- ☐ Aufladen von Handys/Kommunikationsgeräten
- ☐ Medizinische Erstversorgung
- ☐ Ausgabe von Lebensmitteln
- ☐ Notunterkunft
- ☐ Kontakt zu Behörden/Rettungskräften
- ☐ Weiß ich nicht
- ☐ Sonstiges

Welche sonstigen Funktionen sollten solche Anlaufpunkte im Krisenfall erfüllen?

Beantworten Sie diese Frage nur, wenn folgende Bedingungen erfüllt sind:
Antwort war bei Frage ' [Q047]' (Welche Funktionen sollten solche Anlaufpunkte im Krisenfall erfüllen?)

Bitte geben Sie Ihre Antwort hier ein:

5. ERSTE HILFE UND ALLTAGSKOMPETENZEN

Haben Sie einen Erste-Hilfe-Kurs absolviert? *

Bitte wählen Sie eine der folgenden Antworten:
Bitte wählen Sie nur eine der folgenden Antworten aus:

- ◯ Nein, noch nie
- ◯ Ja, in den letzten 2 Jahren
- ◯ Ja, vor 3-5 Jahren
- ◯ Ja, vor 6-10 Jahren
- ◯ Ja, aber länger als 10 Jahre her

Wie schätzen Sie Ihre Kenntnisse in folgenden Erste-Hilfe-Maßnahmen ein? *

Bitte wählen Sie die zutreffende Antwort für jeden Punkt aus:

	keine Kenntnisse	Schlecht	Mittelmäßig	Gut	Sehr gut
**Stabile Seitenlage**	○	○	○	○	○
**Herz-Lungen-Wiederbelebung(HLW)**	○	○	○	○	○
**Stoppen von Blutungen**	○	○	○	○	○
**Versorgung von Wunden**	○	○	○	○	○
**Umgang mit Bewusstlosigkeit**	○	○	○	○	○
**Verhalten bei Schock**	○	○	○	○	○
**Umgang mit Verbrennungen**	○	○	○	○	○

Wären Sie bereit, Ihre Erste-Hilfe-Kenntnisse durch einen Auffrischungskurs zu verbessern? *

Bitte wählen Sie eine der folgenden Antworten:
Bitte wählen Sie nur eine der folgenden Antworten aus:

- ◯ Ja, auf jeden Fall
- ◯ Ja, wahrscheinlich
- ◯ Vielleicht
- ◯ Eher nein
- ◯ Nein, sicher nicht

Was würde Sie motivieren, an einem Erste-Hilfe-Kurs teilzunehmen? *

Wählen Sie alle zutreffenden Optionen
Bitte wählen Sie alle zutreffenden Antworten aus:

- ☐ Kostenloses Angebot
- ☐ Kurs in der Nähe meines Wohnorts
- ☐ Flexible Kurszeiten (Abend/Wochenende)
- ☐ Kurze Kursdauer
- ☐ Praxisnahe Übungen
- ☐ Online-Angebote/E-Learning-Elemente
- ☐ Empfehlung durch Arbeitgeber/Gemeinde
- ☐ Gutscheine/Anreize
- ☐ Familien-/Gruppenkurse
- ☐ Nichts würde mich motivieren
- ☐ Sonstiges:

Was würde Sie sonst noch motivieren, an einem Erste-Hilfe-Kurs teilzunehmen?

Beantworten Sie diese Frage nur, wenn folgende Bedingungen erfüllt sind:
Antwort war bei Frage ' [Q054]' (Was würde Sie motivieren, an einem Erste-Hilfe-Kurs teilzunehmen?)

Bitte geben Sie Ihre Antwort hier ein:

Haben Sie einen Erste-Hilfe-Kasten zu Hause? *

Bitte wählen Sie eine der folgenden Antworten:
Bitte wählen Sie nur eine der folgenden Antworten aus:

- ◯ Ja, vollständig und aktuell
- ◯ Ja, aber ich bin mir nicht sicher, ob alles vollständig/aktuell ist
- ◯ Nein, habe keinen
- ◯ Weiß ich nicht

Haben Sie einen Erste-Hilfe-Kasten in Ihrem Fahrzeug (falls vorhanden)? *

Bitte wählen Sie eine der folgenden Antworten:

Bitte wählen Sie nur eine der folgenden Antworten aus:

- ◯ Ja, und ich habe ihn regelmäßig überprüft
- ◯ Ja, aber ich habe ihn lange nicht überprüft
- ◯ Nein
- ◯ Ich besitze kein Fahrzeug
- ◯ Weiß ich nicht

Wären Sie bereit, Ihre besonderen Fähigkeiten im Krisenfall anderen zur Verfügung zu stellen? *

Bitte wählen Sie eine der folgenden Antworten:

Bitte wählen Sie nur eine der folgenden Antworten aus:

- ◯ Ja, auf jeden Fall
- ◯ Ja, wahrscheinlich
- ◯ Vielleicht
- ◯ Wahrscheinlich nicht
- ◯ Nein, sicher nicht
- ◯ Habe keine besonderen Fähigkeiten

Haben Sie spezielle medizinische Kenntnisse oder Ausbildungen? *

Wählen Sie alle zutreffenden Optionen
Bitte wählen Sie alle zutreffenden Antworten aus:

- [ ] Sanitätsausbildung
- [ ] Rettungssanitäter/Rettungsassistent/Notfallsanitäter
- [ ] Pflegeausbildung
- [ ] Medizinische Ausbildung/Studium
- [ ] Betriebliche Ersthelfer-Ausbildung
- [ ] Keine medizinischen Ausbildungen
- [ ] Sonstiges

Welche sonstigen speziellen medizinischen Kenntnisse oder Ausbildungen haben Sie?

Beantworten Sie diese Frage nur, wenn folgende Bedingungen erfüllt sind:
Antwort war bei Frage ' [Q058]' (Haben Sie spezielle medizinische Kenntnisse oder Ausbildungen?)

Bitte geben Sie Ihre Antwort hier ein:

6. KATASTROPHENVORSORGE UND EIGENVERANTWORTUNG

Haben Sie sich bereits mit den Empfehlungen des Bundesamts für Bevölkerungsschutz und Katastrophenhilfe (BBK) zur Notfallvorsorge beschäftigt? *

Bitte wählen Sie eine der folgenden Antworten:
Bitte wählen Sie nur eine der folgenden Antworten aus:

- ◯ Ja, intensiv
- ◯ Ja, oberflächlich
- ◯ Habe davon gehört, aber nicht näher angeschaut
- ◯ Nein, kenne ich nicht

Für wie viele Tage hätten Sie derzeit Lebensmittelvorräte zu Hause? *

Bitte wählen Sie eine der folgenden Antworten:
Bitte wählen Sie nur eine der folgenden Antworten aus:

- ◯ Weniger als 1 Tag
- ◯ 1-3 Tage
- ◯ 4-6 Tage
- ◯ 7-10 Tage
- ◯ Mehr als 10 Tage
- ◯ Weiß ich nicht genau

Haben Sie folgende Vorräte zu Hause? *

Bitte wählen Sie die zutreffende Antwort für jeden Punkt aus:

	Nein	Ja, etwas	Ja, ausreichend	Weiß nicht
**Trinkwasser (Flaschen/Kanister)**	○	○	○	○
**Haltbare Lebensmittel (Konserven, etc.)**	○	○	○	○
**Hygieneartikel**	○	○	○	○
**Medikamente (Hausapotheke)**	○	○	○	○
**Batterien/Akkus**	○	○	○	○
**Taschenlampe**	○	○	○	○
**Kerzen/Streichhölzer/Feuerzeug**	○	○	○	○
**Batteriebetriebenes Radio**	○	○	○	○
**Campingkocher/alternative Kochmöglichkeiten**	○	○	○	○
**Powerbank für Smartphone**	○	○	○	○

Haben Sie wichtige Dokumente griffbereit für den Notfall zusammengestellt? *

Bitte wählen Sie eine der folgenden Antworten:

Bitte wählen Sie nur eine der folgenden Antworten aus:

- ◯ Ja, in einer Notfallmappe/Dokumentenmappe
- ◯ Teilweise vorbereitet
- ◯ Nein, aber ich weiß, wo die Dokumente sind
- ◯ Nein, habe ich nicht bedacht

Welche der folgenden Dokumente könnten Sie im Notfall schnell finden? *

Wählen Sie alle zutreffenden Optionen

Bitte wählen Sie alle zutreffenden Antworten aus:

- ☐ Personalausweis/Reisepass
- ☐ Geburtsurkunde
- ☐ Impfpass
- ☐ Versicherungspolicen
- ☐ Bankunterlagen
- ☐ Grundbuchauszug/Mietvertrag
- ☐ Testament/Vorsorgevollmacht
- ☐ Wichtige Passwörter/PINs (schriftlich)
- ☐ Keines dieser Dokumente

Haben Sie Bargeldreserven zu Hause für den Notfall? *

Bitte wählen Sie eine der folgenden Antworten:
Bitte wählen Sie nur eine der folgenden Antworten aus:

- ◯ Ja, ausreichend für mehrere Tage
- ◯ Ja, eine kleine Reserve
- ◯ Nein
- ◯ Möchte ich nicht angeben

Wie hoch schätzen Sie die finanzielle Belastung ein, einen Notvorrat anzulegen? *

Bitte wählen Sie eine der folgenden Antworten:
Bitte wählen Sie nur eine der folgenden Antworten aus:

- ◯ Sehr hoch – kann ich mir nicht leisten
- ◯ Hoch – wäre eine erhebliche Belastung
- ◯ Mittel – wäre machbar mit Planung
- ◯ Niedrig – gut leistbar
- ◯ Sehr niedrig – kein Problem

Welche Hindernisse halten Sie davon ab, Notfallvorräte anzulegen? *

Wählen Sie alle zutreffenden Optionen
Bitte wählen Sie alle zutreffenden Antworten aus:

- ☐ Zu teuer
- ☐ Zu wenig Platz in der Wohnung
- ☐ Weiß nicht, was genau ich brauche
- ☐ Keine Zeit dafür
- ☐ Halte es für übertrieben/unnötig
- ☐ Vertraue darauf, dass der Staat/die Gemeinde hilft
- ☐ Haltbarkeit der Lebensmittel ist ein Problem
- ☐ Keine Hindernisse – ich habe bereits Vorräte
- ☐ Sonstiges

Welche weiteren Hindernisse halten Sie davon ab, Notfallvorräte anzulegen?

Beantworten Sie diese Frage nur, wenn folgende Bedingungen erfüllt sind:
Antwort war bei Frage ' [Q068]' (Welche Hindernisse halten Sie davon ab, Notfallvorräte anzulegen?)

Bitte geben Sie Ihre Antwort hier ein:

Besitzen Sie alternative Energiequellen für den Fall eines längeren Stromausfalls? *

Wählen Sie alle zutreffenden Optionen

Bitte wählen Sie alle zutreffenden Antworten aus:

- [ ] Notstromaggregat
- [ ] Solaranlage mit Batteriespeicher/Inselbetrieb
- [ ] Balkonkraftwerk
- [ ] Kamin/Ofen (mit Brennstoffvorrat)
- [ ] Gasheizung (unabhängig vom Stromnetz)
- [ ] Keine alternativen Energiequellen
- [ ] Weiß ich nicht
- [ ] Sonstiges

Welche weiteren alternativen Energiequellen besitzen Sie?

Beantworten Sie diese Frage nur, wenn folgende Bedingungen erfüllt sind:
Antwort war bei Frage ' [Q069]' (Besitzen Sie alternative Energiequellen für den Fall eines längeren Stromausfalls?)

Bitte geben Sie Ihre Antwort hier ein:

Wie würden Sie sich in Ihrer Wohnung im Winter bei einem mehrtägigen Stromausfall warmhalten? *

Wählen Sie alle zutreffenden Optionen

Bitte wählen Sie alle zutreffenden Antworten aus:

- ☐ Mit Kamin/Ofen
- ☐ Mit Heizlüfter/Heizstrahler (über Notstrom)
- ☐ Mit Gasheizung (funktioniert ohne Strom)
- ☐ Mit warmer Kleidung/Decken
- ☐ Würde zu Verwandten/Freunden/Notunterkunft gehen
- ☐ Weiß ich nicht
- ☐ Sonstiges

Was wären sonstige Wege, wie Sie sich bei einem mehrtägigen Stromausfall warmhalten würden?

Beantworten Sie diese Frage nur, wenn folgende Bedingungen erfüllt sind:
Antwort war bei Frage ' [Q0610]' (Wie würden Sie sich in Ihrer Wohnung im Winter bei einem mehrtägigen Stromausfall warmhalten?)

Bitte geben Sie Ihre Antwort hier ein:

Könnten Sie im Notfall ohne Strom kochen oder Wasser erhitzen? *

Wählen Sie alle zutreffenden Optionen
Bitte wählen Sie alle zutreffenden Antworten aus:

- ☐ Ja, mit Campingkocher/ Gaskocher
- ☐ Ja, mit Grill (Gas/ Holzkohle)
- ☐ Ja, mit Kamin/ Ofen
- ☐ Ja, mit anderer Methode
- ☐ Nein, das wäre nicht möglich

Mit welcher anderen Methode könnten Sie im Notfall ohne Strom kochen oder Wasser erhitzen?

Beantworten Sie diese Frage nur, wenn folgende Bedingungen erfüllt sind:
Antwort war bei Frage ' [Q071]' (Könnten Sie im Notfall ohne Strom kochen oder Wasser erhitzen?)

Bitte geben Sie Ihre Antwort hier ein:

Kennen Sie die Empfehlungen zur Trinkwasserbevorratung (2 Liter pro Person und Tag)? *

Bitte wählen Sie eine der folgenden Antworten:

Bitte wählen Sie nur eine der folgenden Antworten aus:

- ◯ Ja, und ich halte mich daran
- ◯ Ja, aber ich halte mich nicht daran
- ◯ Nein, kannte ich nicht

Wissen Sie, wie Sie im Notfall an Trinkwasser gelangen könnten, wenn die Wasserversorgung ausfällt? *

Bitte wählen Sie eine der folgenden Antworten:

Bitte wählen Sie nur eine der folgenden Antworten aus:

- ◯ Ja, ich kenne mehrere Möglichkeiten
- ◯ Ja, ich habe eine Idee
- ◯ Nein, weiß ich nicht
- ◯ Verlasse mich auf meine Vorräte

7. SOZIALE RESILIENZ UND NACHBARSCHAFTSHILFE

Wie gut kennen Sie Ihre direkten Nachbarn? *

Bitte wählen Sie eine der folgenden Antworten:
Bitte wählen Sie nur eine der folgenden Antworten aus:

- ◯ Gar nicht
- ◯ Kaum – ich weiß nicht, wer meine Nachbarn sind
- ◯ Es geht – wir kennen uns vom Sehen
- ◯ Gut – wir grüßen uns und tauschen uns aus
- ◯ Sehr gut – wir haben engen Kontakt

Würden Sie im Notfall Hilfe von Ihren Nachbarn erwarten? *

Bitte wählen Sie eine der folgenden Antworten:
Bitte wählen Sie nur eine der folgenden Antworten aus:

- ◯ Ja, auf jeden Fall
- ◯ Wahrscheinlich ja
- ◯ Vielleicht
- ◯ Wahrscheinlich nicht
- ◯ Nein, sicher nicht
- ◯ Habe keine Nachbarn in der Nähe

Würden Sie im Notfall Ihre Nachbarn unterstützen? *

Bitte wählen Sie eine der folgenden Antworten:
Bitte wählen Sie nur eine der folgenden Antworten aus:

- ◯ Ja, auf jeden Fall
- ◯ Wahrscheinlich ja
- ◯ Vielleicht
- ◯ Wahrscheinlich nicht
- ◯ Nein, sicher nicht

Welche sozialen Strukturen sind Ihrer Meinung nach im Krisenfall besonders wichtig? *

Wählen Sie alle zutreffenden Optionen
Bitte wählen Sie alle zutreffenden Antworten aus:

- ☐ Nachbarschaftshilfe
- ☐ Familie/Verwandte
- ☐ Freundeskreis
- ☐ Vereine (Sport, Kultur, etc.)
- ☐ Kirchengemeinde/Religionsgemeinschaft
- ☐ Selbsthilfegruppen
- ☐ Professionelle Hilfsorganisationen (Feuerwehr, THW, etc.)
- ☐ Gemeinde/Stadtverwaltung
- ☐ Online-Communities/Soziale Netzwerke
- ☐ Sonstiges

Welche weiteren sozialen Strukturen sind Ihrer Meinung nach im Krisenfall besonders wichtig?

Beantworten Sie diese Frage nur, wenn folgende Bedingungen erfüllt sind:
Antwort war bei Frage ' [Q074]' (Welche sozialen Strukturen sind Ihrer Meinung nach im Krisenfall besonders wichtig?)

Bitte geben Sie Ihre Antwort hier ein:

Im Krisenfall könnten gewohnte Strukturen (Arbeit, Schule, Vereine) wegfallen. An wen würden Sie sich zuerst wenden?

Alle Ihre Antworten müssen unterschiedlich sein, und müssen zugeordnet sein.
Bitte wählen Sie maximal 8 Antworten.
Bitte nummerieren Sie jede Box in der Reihenfolge Ihrer Präferenz, beginnen mit 1 bis 8

[ ] Familie/nahe Verwandte

[ ] Freunde

[ ] Nachbarn

[ ] Gemeinde/Behörden

[ ] Hilfsorganisationen (Feuerwehr, etc.)

[ ] Kirchengemeinde

[ ] Weiß ich nicht

[ ] Sonstiges

Gibt es in Ihrer Nachbarschaft organisierte Strukturen für gegenseitige Hilfe? (z.B. Nachbarschaftsgruppen, WhatsApp-Gruppen) *

Bitte wählen Sie eine der folgenden Antworten:
Bitte wählen Sie nur eine der folgenden Antworten aus:

- Ja, und ich bin aktiv dabei
- Ja, aber ich bin nicht dabei
- Nein, aber das wäre wünschenswert
- Nein, und ich sehe keinen Bedarf
- Weiß ich nicht

Welche Art von Nachbarschaftshilfe können Sie sich im Krisenfall vorstellen? *

Wählen Sie alle zutreffenden Optionen
Bitte wählen Sie alle zutreffenden Antworten aus:

- [ ] Gemeinsame Nutzung von Ressourcen (Wasser, Lebensmittel)
- [ ] Abwechselnde Wache/Sicherheit
- [ ] Kinderbetreuung
- [ ] Pflege von älteren/kranken Nachbarn
- [ ] Teilen von Informationen
- [ ] Gemeinsames Kochen
- [ ] Handwerkliche Hilfe/Reparaturen
- [ ] Psychologische Unterstützung/Gespräche
- [ ] Keine
- [ ] Sonstiges

Welche weitere Nachbarschaftshilfe können Sie sich im Krisenfall vorstellen?

Beantworten Sie diese Frage nur, wenn folgende Bedingungen erfüllt sind:
Antwort war bei Frage ' [Q077]' (Welche Art von Nachbarschaftshilfe können Sie sich im Krisenfall vorstellen?)

Bitte geben Sie Ihre Antwort hier ein:

8. KOMMUNIKATION UND INFORMATION IM KRISENFALL

Welche Kommunikationsmittel würden Ihnen im Krisenfall zur Verfügung stehen? *

Wählen Sie alle zutreffenden Optionen
Bitte wählen Sie alle zutreffenden Antworten aus:

- ☐ Smartphone (solange Akku reicht)
- ☐ Festnetztelefon
- ☐ Batteriebetriebenes Radio
- ☐ Kurbelradio/Solar-Radio
- ☐ Internetzugang (solange Strom da ist)
- ☐ Mobilfunkdaten
- ☐ CB-Funk/Amateurfunk
- ☐ Keine dieser Möglichkeiten
- ☐ Weiß ich nicht

Haben Sie Warn-Apps auf Ihrem Smartphone installiert? *

Wählen Sie alle zutreffenden Optionen
Bitte wählen Sie alle zutreffenden Antworten aus:

- ☐ Ja, NINA
- ☐ Ja, KATWARN
- ☐ Ja, WarnWetter
- ☐ Ja, andere Warn-App
- ☐ Nein, keine Warn-App
- ☐ Ich habe kein Smartphone

Welche weiteren Warn-Apps haben Sie auf Ihrem Smartphone installiert?

Beantworten Sie diese Frage nur, wenn folgende Bedingungen erfüllt sind:
Antwort war bei Frage ' [Q082]' (Haben Sie Warn-Apps auf Ihrem Smartphone installiert?)

Bitte geben Sie Ihre Antwort hier ein:

Falls Sie Warn-Apps installiert haben: Wie häufig schauen Sie nach Warnmeldungen?

Beantworten Sie diese Frage nur, wenn folgende Bedingungen erfüllt sind:
((Q082_SQ002.NAOK == "Y") or (Q082_SQ003.NAOK == "Y")) or (Q082_SQ004.NAOK == "Y")) or (Q082_SQ005.NAOK == "Y")) or

Bitte wählen Sie eine der folgenden Antworten:
Bitte wählen Sie nur eine der folgenden Antworten aus:

- ◯ Täglich
- ◯ Mehrmals pro Woche
- ◯ Einmal pro Woche
- ◯ Seltener
- ◯ Nur wenn ich eine Benachrichtigung bekomme
- ◯ Nie

Würden Sie im Ernstfall aktiv nach Informationen suchen oder darauf warten, dass Sie informiert werden? *

Bitte wählen Sie eine der folgenden Antworten:
Bitte wählen Sie nur eine der folgenden Antworten aus:

- ◯ Ich würde aktiv nach Informationen suchen
- ◯ Ich würde beides tun
- ◯ Ich würde darauf warten, informiert zu werden
- ◯ Weiß ich nicht

Welche Art von Informationen wären im Krisenfall für Sie am wichtigsten? *

Wählen Sie alle zutreffenden Optionen

Bitte wählen Sie alle zutreffenden Antworten aus:

- ☐ Art und Ausmaß der Gefahr
- ☐ Verhaltensanweisungen (was ist zu tun?)
- ☐ Dauer der Krise/Zeitplan
- ☐ Anlaufstellen für Hilfe
- ☐ Versorgungsmöglichkeiten (Wasser, Lebensmittel)
- ☐ Medizinische Versorgung
- ☐ Information über Angehörige
- ☐ Kontaktmöglichkeiten zu Behörden
- ☐ Aktuelle Lage/Entwicklungen
- ☐ Sonstiges

Welche weiteren Arten von Informationen wären im Krisenfall für Sie am wichtigsten?

Beantworten Sie diese Frage nur, wenn folgende Bedingungen erfüllt sind:
Antwort war bei Frage ' [Q085]' (Welche Art von Informationen wären im Krisenfall für Sie am wichtigsten?)

Bitte geben Sie Ihre Antwort hier ein:

9. ERWARTUNGEN AN STAATLICHE EBENEN UND

VERANTWORTLICHKEITEN

Wer ist Ihrer Meinung nach in erster Linie für Ihre Sicherheit im Krisenfall verantwortlich? *

Bitte wählen Sie eine der folgenden Antworten:
Bitte wählen Sie nur eine der folgenden Antworten aus:

- ◯ Ich selbst/meine Familie
- ◯ Meine Gemeinde/Stadt
- ◯ Der Kreis
- ◯ Das Bundesland
- ◯ Der Bund
- ◯ Alle gemeinsam
- ◯ Weiß ich nicht

Was erwarten Sie von Ihrer Gemeinde/Stadt im Krisenfall? *

Wählen Sie alle zutreffenden Optionen
Bitte wählen Sie alle zutreffenden Antworten aus:

- ☐ Rechtzeitige Warnung
- ☐ Klare Handlungsanweisungen
- ☐ Bereitstellung von Notunterkünften
- ☐ Ausgabe von Wasser/Lebensmitteln
- ☐ Einrichtung von Anlaufpunkten
- ☐ Information über die Lage
- ☐ Koordination von Hilfskräften
- ☐ Unterstützung hilfsbedürftiger Personen
- ☐ Aufrechterhaltung von Ordnung und Sicherheit
- ☐ Nichts davon
- ☐ Sonstiges

Was erwarten Sie außerdem von Ihrer Gemeinde/Stadt im Krisenfall?

Beantworten Sie diese Frage nur, wenn folgende Bedingungen erfüllt sind:
Antwort war bei Frage ' [Q092]' (Was erwarten Sie von Ihrer Gemeinde/Stadt im Krisenfall?)

Bitte geben Sie Ihre Antwort hier ein:

Fühlen Sie sich ausreichend über die Vorbereitungen Ihrer Gemeinde/Ihres Kreises auf Krisensituationen informiert? *

Bitte wählen Sie eine der folgenden Antworten:
Bitte wählen Sie nur eine der folgenden Antworten aus:

- ◯ Ja, vollkommen ausreichend
- ◯ Eher ja
- ◯ Teils/teils
- ◯ Eher nein
- ◯ Nein, überhaupt nicht

Würden Sie gerne mehr darüber erfahren, wie Ihre Gemeinde/Ihr Kreis sich auf Krisen vorbereitet? *

Bitte wählen Sie eine der folgenden Antworten:
Bitte wählen Sie nur eine der folgenden Antworten aus:

- ◯ Ja, auf jeden Fall
- ◯ Ja, wäre interessant
- ◯ Ist mir egal
- ◯ Nein, das interessiert mich nicht

Vertrauen Sie darauf, dass staatliche Stellen im Krisenfall handlungsfähig sind? *

Bitte wählen Sie die zutreffende Antwort für jeden Punkt aus:

	Gar nicht	Eher nein	Teils/ teils	Eher Ja	Voll und ganz
**Ihre Gemeinde/ Stadt**	○	○	○	○	○
**Der Kreis**	○	○	○	○	○
**Das Bundesland**	○	○	○	○	○
**Der Bund**	○	○	○	○	○

Vertrauen Sie darauf, dass im Krisenfall Ressourcen gerecht verteilt werden? *

Bitte wählen Sie eine der folgenden Antworten:
Bitte wählen Sie nur eine der folgenden Antworten aus:

- ○ Ja, voll und ganz
- ○ Eher ja
- ○ Teils/teils
- ○ Eher nein
- ○ Nein, überhaupt nicht

Sollte die Gemeinde/der Kreis mehr in kommunale Vorsorgestrukturen investieren? (z.B. Notvorräte, Anlaufpunkte, Warnsysteme) *

Bitte wählen Sie eine der folgenden Antworten:

Bitte wählen Sie nur eine der folgenden Antworten aus:

- ◯ Ja, unbedingt
- ◯ Ja, wäre sinnvoll
- ◯ Teils/teils
- ◯ Nein, nicht notwendig
- ◯ Weiß ich nicht

Welche Vorsorgemaßnahmen der Gemeinde/des Kreises wären Ihnen am wichtigsten?

Alle Ihre Antworten müssen unterschiedlich sein, und müssen zugeordnet sein.
Bitte wählen Sie maximal 10 Antworten.
Bitte nummerieren Sie jede Box in der Reihenfolge Ihrer Präferenz, beginnen mit 1 bis 10

- [ ] Notvorräte (Wasser, Lebensmittel)
- [ ] Notstrom-Aggregate für wichtige Einrichtungen
- [ ] Medizinische Notfallausrüstung
- [ ] Besser ausgestattete Feuerwehren/THW
- [ ] Mehr Informationskampagnen für Bürger
- [ ] Deutlich gekennzeichnete Anlaufpunkte
- [ ] Bessere Warnsysteme
- [ ] Notunterkünfte
- [ ] Weiß ich nicht
- [ ] Sonstiges:

10. SICHERHEIT, ORDNUNG UND GESELLSCHAFTLICHE STABILITÄT

Machen Sie sich Sorgen über mögliche Kriminalität oder Plünderungen im Krisenfall? *

Bitte wählen Sie eine der folgenden Antworten:

Bitte wählen Sie nur eine der folgenden Antworten aus:

- ◯ Ja, große Sorgen
- ◯ Ja, einige Sorgen
- ◯ Etwas Sorgen
- ◯ Kaum Sorgen
- ◯ Nein, keine Sorgen

Haben Sie Maßnahmen zum Eigenschutz in Krisensituationen überlegt oder getroffen? *

Bitte wählen Sie eine der folgenden Antworten:

Bitte wählen Sie nur eine der folgenden Antworten aus:

- ◯ Ja, konkrete Maßnahmen getroffen
- ◯ Ja, darüber nachgedacht, aber nichts umgesetzt
- ◯ Nein, habe ich nicht

Welche Maßnahmen haben Sie zum Eigenschutz in Krisensituationen überlegt oder getroffen?

Beantworten Sie diese Frage nur, wenn folgende Bedingungen erfüllt sind:
Antwort war NICHT 'Nein, habe ich nicht' bei Frage ' [Q1002]' (Haben Sie Maßnahmen zum Eigenschutz in Krisensituationen überlegt oder getroffen?)

Bitte geben Sie Ihre Antwort hier ein:

Vertrauen Sie darauf, dass Polizei und Sicherheitskräfte im Krisenfall Ordnung aufrechterhalten können? *

Bitte wählen Sie eine der folgenden Antworten:
Bitte wählen Sie nur eine der folgenden Antworten aus:

- ◯ Ja, voll und ganz
- ◯ Eher ja
- ◯ Teils/teils
- ◯ Eher nein
- ◯ Nein, überhaupt nicht

Wie stabil schätzen Sie den gesellschaftlichen Zusammenhalt in Deutschland im Krisenfall ein? *

Bitte wählen Sie eine der folgenden Antworten:

Bitte wählen Sie nur eine der folgenden Antworten aus:

- ◯ Sehr stabil – die Gesellschaft würde zusammenhalten
- ◯ Eher stabil – es würde im Großen und Ganzen funktionieren
- ◯ Teils/teils – in manchen Bereichen ja, in anderen nein
- ◯ Eher instabil – es könnte zu Konflikten kommen
- ◯ Sehr instabil – die Ordnung würde schnell zusammenbrechen
- ◯ Kann ich nicht einschätzen

Welche Faktoren würden Ihrer Meinung nach den sozialen Zusammenhalt im Krisenfall am meisten gefährden?

Alle Ihre Antworten müssen unterschiedlich sein, und müssen zugeordnet sein.
Bitte wählen Sie maximal 11 Antworten.
Bitte nummerieren Sie jede Box in der Reihenfolge Ihrer Präferenz, beginnen mit 1 bis 11

- [ ] Ungerechte Verteilung von Lebensmitteln
- [ ] Mangelnde Information
- [ ] Gerüchte und Falschinformationen
- [ ] Egoismus einzelner Personen/Gruppen
- [ ] Kriminalität/Plünderungen
- [ ] Ethnische/soziale Spannungen
- [ ] Überforderung der Behörden
- [ ] Dauer der Krise
- [ ] Mangelnde Vorbereitung der Bevölkerung
- [ ] Nichts würde den Zusammenhalt gefährden
- [ ] Sonstiges

Vertrauen Sie darauf, dass nach einer Krise die rechtsstaatliche Ordnung wiederhergestellt wird? *

Bitte wählen Sie eine der folgenden Antworten:
Bitte wählen Sie nur eine der folgenden Antworten aus:

- ◯ Ja, voll und ganz
- ◯ Eher ja
- ◯ Teils/teils
- ◯ Eher nein
- ◯ Nein, überhaupt nicht

Wie würden Sie sich fühlen, wenn staatliche Notstandsmaßnahmen (z.B. Ausgangssperren, Rationierungen) verhängt würden? *

Bitte wählen Sie eine der folgenden Antworten:
Bitte wählen Sie nur eine der folgenden Antworten aus:

- ◯ Sicher – notwendige Maßnahmen für den Schutz aller
- ◯ Eher sicher – kann ich nachvollziehen
- ◯ Ambivalent – kommt auf die konkrete Situation an
- ◯ Eher unsicher – Sorge vor Machtmissbrauch
- ◯ Sehr unsicher – lehne ich ab

Welche Rolle sollte die Polizei im Krisenfall einnehmen?

Alle Ihre Antworten müssen unterschiedlich sein, und müssen zugeordnet sein.
Bitte wählen Sie maximal 9 Antworten.
Bitte nummerieren Sie jede Box in der Reihenfolge Ihrer Präferenz, beginnen mit 1 bis 9

- [ ] Aufrechterhaltung von Ordnung und Sicherheit
- [ ] Schutz kritischer Infrastruktur
- [ ] Kontrolle von Ausgangssperren/Regelungen
- [ ] Verhinderung von Plünderungen
- [ ] Koordination mit anderen Hilfskräften
- [ ] Unterstützung bei Evakuierungen
- [ ] Nur bei schweren Straftaten eingreifen
- [ ] Weiß ich nicht
- [ ] Sonstiges

11. ROLLE DER BUNDESWEHR UND ZIVIL-MILITÄRISCHE ZUSAMMENARBEIT

In welchen Bereichen sollte die Bundeswehr im Krisenfall tätig werden dürfen? *

Wählen Sie alle zutreffenden Optionen
Bitte wählen Sie alle zutreffenden Antworten aus:

- ☐ Logistik/Transport
- ☐ Medizinische Versorgung
- ☐ Aufbau von Notunterkünften
- ☐ Verteilung von Hilfsgütern
- ☐ Technische Hilfe (z.B. Brückenbau)
- ☐ Unterstützung bei Evakuierungen
- ☐ Aufrechterhaltung der Ordnung
- ☐ Schutz kritischer Infrastruktur
- ☐ In allen Bereichen, wo Hilfe nötig ist
- ☐ In keinem Bereich
- ☐ Weiß ich nicht

Wie schätzen Sie die Zusammenarbeit zwischen zivilen Hilfskräften (Feuerwehr, THW, etc.) und der Bundeswehr im Krisenfall ein? *

Bitte wählen Sie eine der folgenden Antworten:
Bitte wählen Sie nur eine der folgenden Antworten aus:

- ◯ Sehr wichtig und funktioniert gut
- ◯ Wichtig, aber könnte besser funktionieren
- ◯ Mittelmäßig wichtig
- ◯ Weniger wichtig
- ◯ Unwichtig oder problematisch
- ◯ Kann ich nicht einschätzen

Haben Sie Vertrauen in die Fähigkeiten der Bundeswehr bei Katastropheneinsätzen im Inland? *

Bitte wählen Sie eine der folgenden Antworten:

Bitte wählen Sie nur eine der folgenden Antworten aus:

- ◯ Ja, großes Vertrauen
- ◯ Ja, eher Vertrauen
- ◯ Teils/teils
- ◯ Eher kein Vertrauen
- ◯ Nein, kein Vertrauen
- ◯ Kann ich nicht einschätzen

12. MITWIRKUNGSBEREITSCHAFT UND ENGAGEMENT

Was würde Sie motivieren, sich im Bevölkerungsschutz zu engagieren? *

Bitte wählen Sie die zutreffende Antwort für jeden Punkt aus:

	Gar nicht	Eher nein	Teils/teils	Eher ja	Voll und ganz
**Sinnvolle Tätigkeit**	○	○	○	○	○
**Helfen wollen**	○	○	○	○	○
**Gemeinschaft/Kameradschaft**	○	○	○	○	○
**Neue Fähigkeiten lernen**	○	○	○	○	○
**Abwechslung zum Alltag**	○	○	○	○	○
**Anerkennung/Wertschätzung**	○	○	○	○	○
**Vergünstigungen (z.B. Steuervorteile, Freistellungen)**	○	○	○	○	○
**Flexible Zeiteinteilung**	○	○	○	○	○
**Kurze Verpflichtungsdauer**	○	○	○	○	○
**Gute Ausbildung**	○	○	○	○	○

Was würde Sie davon abhalten, sich zu engagieren? *

Bitte wählen Sie die zutreffende Antwort für jeden Punkt aus:

	Trifft nicht zu	Trifft eher nicht zu	Teils/teils	Trifft eher zu	Trifft voll und ganz zu
**Zu wenig Zeit im Alltag (Beruf/Familie/private Verpflichtungen)**	○	○	○	○	○
**Gesundheitliche Gründe**	○	○	○	○	○
**Zu hohe körperliche Anforderungen**	○	○	○	○	○
**Zu hoher zeitlicher Umfang pro Einsatz/Dienst**	○	○	○	○	○
**Fehlende Informationen über Möglichkeiten**	○	○	○	○	○
**Angst vor Überforderung**	○	○	○	○	○
**Mangelnde Unterstützung durch Arbeitgeber**	○	○	○	○	○
**Mangelnde Anerkennung**	○	○	○	○	○
**Bürokratischer Aufwand**	○	○	○	○	○
**Alter**	○	○	○	○	○

Wie wichtig ist Ihnen die Anerkennung ehrenamtlichen Engagements im Bevölkerungsschutz? *

Bitte wählen Sie eine der folgenden Antworten:

Bitte wählen Sie nur eine der folgenden Antworten aus:

- ◯ Sehr wichtig
- ◯ Wichtig
- ◯ Mittelmäßig wichtig
- ◯ Weniger wichtig
- ◯ Unwichtig

Welche Form der Anerkennung wäre für Sie am bedeutsamsten?

Alle Ihre Antworten müssen unterschiedlich sein, und müssen zugeordnet sein.
Bitte wählen Sie maximal 9 Antworten.
Bitte nummerieren Sie jede Box in der Reihenfolge Ihrer Präferenz, beginnen mit 1 bis 9

- [ ] Öffentliche Wertschätzung/Dankbarkeit
- [ ] Steuerliche Vorteile
- [ ] Freistellung vom Arbeitgeber
- [ ] Versicherungsschutz
- [ ] Kostenlose Aus- und Weiterbildung
- [ ] Bescheinigungen/Zertifikate
- [ ] Finanzielle Aufwandsentschädigung
- [ ] Soziale Gemeinschaft/Zugehörigkeit
- [ ] Keine Anerkennung nötig

Würden Sie im akuten Krisenfall spontan helfen, auch wenn Sie nicht organisiert engagiert sind? *

Bitte wählen Sie eine der folgenden Antworten:
Bitte wählen Sie nur eine der folgenden Antworten aus:

- ◯ Ja, auf jeden Fall
- ◯ Wahrscheinlich ja
- ◯ Vielleicht
- ◯ Wahrscheinlich nicht
- ◯ Nein, sicher nicht

Würden Sie Ihre eigenen Ressourcen (z.B. Generator, Werkzeug, Vorräte) im Krisenfall mit anderen teilen? *

Bitte wählen Sie eine der folgenden Antworten:
Bitte wählen Sie nur eine der folgenden Antworten aus:

- ◯ Ja, großzügig mit allen
- ◯ Ja, mit Nachbarn/Bekannten
- ◯ Ja, mit Freunden/Familie
- ◯ Nur im äußersten Notfall
- ◯ Nein, habe nur für mich vorgesorgt
- ◯ Habe keine besonderen Ressourcen

13. MOTIVATION UND AKTIVIERUNG

Was würde Sie persönlich dazu bewegen, sich intensiver mit Krisenvorsorge zu beschäftigen? *

Wählen Sie alle zutreffenden Optionen

Bitte wählen Sie alle zutreffenden Antworten aus:

- [ ] Konkrete Anleitung/Checklisten
- [ ] Kostenlose Informationsmaterialien
- [ ] Informationsveranstaltungen in der Gemeinde
- [ ] Praktische Übungen/Kurse
- [ ] Persönliche Betroffenheit/Erfahrung
- [ ] Medienberichterstattung über Krisen
- [ ] Empfehlung von Freunden/Familie
- [ ] Empfehlung durch Behörden
- [ ] Finanzielle Unterstützung (z.B. Gutscheine für Notvorräte)
- [ ] Apps/Online-Tools zur Vorbereitung
- [ ] Nichts würde mich dazu bewegen
- [ ] Sonstiges

Was würde Sie außerdem motivieren, sich mit dem Thema zu beschäftigen?

Beantworten Sie diese Frage nur, wenn folgende Bedingungen erfüllt sind:
Antwort war bei Frage ' [Q214088]' (Was würde Sie persönlich dazu bewegen, sich intensiver mit Krisenvorsorge zu beschäftigen?)

Bitte geben Sie Ihre Antwort hier ein:

Sollte Krisenvorsorge stärker in Schulen thematisiert werden? *

Bitte wählen Sie eine der folgenden Antworten:
Bitte wählen Sie nur eine der folgenden Antworten aus:

- ◯ Ja, unbedingt
- ◯ Ja, wäre sinnvoll
- ◯ Teils/teils
- ◯ Eher nein
- ◯ Nein, gehört nicht in die Schule
- ◯ Keine Meinung

Würden Sie sich als "Multiplikator" engagieren und Informationen zur Krisenvorsorge in Ihrem Umfeld weitergeben? *

Bitte wählen Sie eine der folgenden Antworten:
Bitte wählen Sie nur eine der folgenden Antworten aus:

- ◯ Ja, aktiv
- ◯ Ja, wenn ich gefragt werde
- ◯ Vielleicht
- ◯ Wahrscheinlich nicht
- ◯ Nein

14. ABSCHLUSSFRAGEN UND REFLEXION

Hat die Teilnahme an dieser Befragung Ihr Bewusstsein für Krisenvorsorge verändert? *

Bitte wählen Sie eine der folgenden Antworten:

Bitte wählen Sie nur eine der folgenden Antworten aus:

- ◯ Ja, stark
- ◯ Ja, etwas
- ◯ Kaum
- ◯ Nein, gar nicht

Wie stark beschäftigen Sie sich nun – nach dieser Befragung – mit dem Thema Krisenvorsorge? *

Bitte wählen Sie eine der folgenden Antworten:

Bitte wählen Sie nur eine der folgenden Antworten aus:

- ◯ Sehr stark – ich werde aktiv werden
- ◯ Stark – ich werde mich weiter informieren
- ◯ Mittelmäßig – ich habe es im Hinterkopf
- ◯ Wenig – kaum Veränderung
- ◯ Gar nicht – keine Veränderung

Was ist Ihrer Meinung nach die wichtigste Maßnahme, um die Resilienz der Bevölkerung zu stärken? *

Bitte wählen Sie eine der folgenden Antworten:
Bitte wählen Sie nur eine der folgenden Antworten aus:

- ◯ Bessere Information der Bevölkerung
- ◯ Mehr praktische Übungen und Trainings
- ◯ Ausbau der professionellen Katastrophenschutzstrukturen
- ◯ Stärkung der Nachbarschaftshilfe
- ◯ Mehr Eigenverantwortung der Bürger
- ◯ Bessere staatliche Vorsorge
- ◯ Sonstiges

Was ist Ihrer Meinung nach eine wichtige Maßnahmen, um die Resilienz der Bevölkerung zu stärken?

Beantworten Sie diese Frage nur, wenn folgende Bedingungen erfüllt sind:
Antwort war 'Sonstiges' bei Frage ' [Q1403]' (Was ist Ihrer Meinung nach die wichtigste Maßnahme, um die Resilienz der Bevölkerung zu stärken?)

Bitte geben Sie Ihre Antwort hier ein:

Haben Sie Anmerkungen, Anregungen oder Kritik zu dieser Befragung?

Bitte geben Sie Ihre Antwort hier ein:

Möchten Sie über die Ergebnisse dieser Studie informiert werden? *

Bitte wählen Sie eine der folgenden Antworten:
Bitte wählen Sie nur eine der folgenden Antworten aus:

- ◯ Nein
- ◯ Ja, über die Website der Gemeinde

Senden Sie Ihre Umfrage ein.
Vielen Dank für die Beantwortung des Fragebogens.
