# Supplementary material for: Development and consensus-based validation of a comprehensive population survey instrument for municipal crisis preparedness: a three-round Delphi study (KOMPAKT112)
Source: Front Public Health. 2026 Aug 5;14:1883344. doi: 10.3389/fpubh.2026.1883344 (PMC13485894; doi:10.3389/fpubh.2026.1883344)
Supplement: Supplementary file 2 [file Supplementary_file_2.docx]

**Supplementary Material S2 – Item-Level Consensus Data by Round and Inter-Rater Concordance by Thematic Block**

**S2.1 Item-Level Consensus Data by Round**

Table 2.1 reports item-level statistics for all items included in the initial item pool (n = 183), supplemented by items added following panelist proposals during Round 1 (n = 13 new items, marked with *). For each item, the table shows the median relevance rating, interquartile range (IQR), and proportion of panelists rating ≥ 4 on the 5-point Likert scale (consensus rate) across all rounds in which the item was presented. Items deleted after Round 1 (n = 48) or after Round 2 (n = 4 + 29 modified+deleted in R2 process) carry dashes for subsequent rounds. Items with IQR > 1.6 in any round are shaded in the original dataset; stable disagreement items are identified in Supplementary S5. Full item wording is provided in Supplementary S1.

*Decision codes: Retained = no changes made; Retained modified = wording or format revised; Deleted (R1) = below threshold and/or no stable disagreement pathway in Round 1; Deleted (R2) = below threshold after Round 2; Stable disagreement = retained per pre-specified rule despite IQR > 1.0 in Round 3 (see S5); New = item proposed by panel and added for Round 2 rating.*

| **Blk** | **Item No.** | **Item (abbreviated)** | **R1 Median** | **R1 IQR** | **R1 Cons. %** | **R2 Median** | **R2 IQR** | **R2 Cons. %** | **R3 Median** | **R3 IQR** | **R3 Cons. %** | **Decision** |
| --- | --- | --- | --- | --- | --- | --- | --- | --- | --- | --- | --- | --- |
| 1 | 1.1 | Age group | 4.3 | 1.26 | 78.8% | 4.6 | 0.83 | 86.2% | 4.7 | 0.71 | 90.6% | Retained |
|  | 1.2 | Gender | 4.3 | 1.42 | 76.8% | 4.6 | 0.99 | 89.5% | 4.7 | 0.71 | 92.5% | Retained |
|  | 1.3 | Household size | 4.6 | 1.41 | 78.9% | 4.8 | 1.22 | 84.5% | 5.0 | 1.06 | 87.1% | Retained |
|  | 1.4 | Employment status | 2.4 | 2.35 | 25.6 | – | – | – | – | – | – | Deleted (R1) |
|  | 1.5 | Educational level | 4.7 | 1.00 | 72.9% | 4.9 | 0.81 | 84.4% | 5.0 | 0.67 | 89.7% | Retained |
|  | 1.6 | Migration background | 4.2 | 1.07 | 91.8% | 4.5 | 0.75 | 98.4% | – | – | – | Deleted (R2) |
|  | 1.7 | Main residence municipality | 4.2 | 1.23 | 89.8% | 4.5 | 0.91 | 99.9% | 4.7 | 0.73 | 99.9% | Retained modified |
|  | 1.8 | Residence type (urban/rural) | 4.2 | 1.39 | 87.8% | 4.5 | 1.07 | 95.1% | 4.7 | 0.98 | 98.2% | Retained |
|  | 1.9 | Homeownership status | 4.2 | 1.55 | 85.9% | 4.5 | 1.23 | 98.5% | 4.7 | 0.98 | 99.9% | Retained |
|  | 1.10 | Household composition | 4.2 | 0.81 | 83.9% | 4.5 | 0.49 | 91.8% | 4.7 | 0.34 | 99.0% | Retained |
|  | 1.11 | Regular BOS affiliation | 4.2 | 0.98 | 81.9% | 4.5 | 0.67 | 85.1% | 4.7 | 0.61 | 90.8% | Retained |
|  | 1.12 | First responder training | 4.2 | 1.14 | 79.9% | 4.5 | 0.83 | 88.4% | 4.7 | 0.61 | 92.7% | Retained |
|  | 1.13 | Civil protection awareness training | 4.2 | 1.30 | 78.0% | 4.5 | 0.99 | 81.8% | 4.7 | 0.86 | 84.6% | Retained |
|  | 1.14 | Voluntary engagement status | 4.2 | 1.46 | 76.0% | 4.5 | 1.15 | 85.2% | 4.7 | 0.86 | 93.6% | Retained |
|  | 1.15 | Highest vocational qualification | 4.2 | 1.62 | 74.0% | 4.5 | 1.31 | 78.5% | 4.7 | 1.11 | 85.4% | Retained |
|  | 1.16* | [Item 1.16] | 4.2 | 0.88 | 72.0% | 4.3 | 1.36 | 88.7% | 4.5 | 1.25 | 94.2% | New (panelist-proposed) |
| 2 | 2.1 | Perceived personal risk – natural hazards | 2.5 | 2.04 | 32.8 | – | – | – | – | – | – | Deleted (R1) |
|  | 2.2 | Perceived personal risk – power outage | 2.5 | 2.18 | 29.8 | – | – | – | – | – | – | Deleted (R1) |
|  | 2.3 | Perceived personal risk – pandemic | 2.5 | 2.32 | 26.8 | – | – | – | – | – | – | Deleted (R1) |
|  | 2.4 | Perceived personal risk – war/conflict | 2.5 | 1.67 | 23.9 | – | – | – | – | – | – | Deleted (R1) |
|  | 2.5 | Perceived personal risk – terrorism | 4.2 | 1.03 | 72.6% | 4.5 | 0.60 | 82.7% | – | – | – | Deleted (R2) |
|  | 2.6 | Self-assessed preparedness level | 4.2 | 1.20 | 90.6% | 4.5 | 0.77 | 96.1% | 4.6 | 0.60 | 99.9% | Retained modified |
|  | 2.7 | Risk acceptance tendency | 4.3 | 1.36 | 88.7% | 4.6 | 0.93 | 99.5% | 4.7 | 0.85 | 99.9% | Retained modified |
|  | 2.8 | Trust in own crisis-coping ability | 4.3 | 1.52 | 86.7% | 4.6 | 1.09 | 92.8% | 4.7 | 0.85 | 96.0% | Retained |
|  | 2.9 | Optimism bias self-assessment | 4.3 | 1.68 | 84.7% | 4.6 | 1.25 | 96.1% | 4.7 | 1.10 | 99.9% | Retained |
|  | 2.10 | Comparative risk perception | 4.3 | 0.94 | 82.7% | 4.6 | 0.51 | 89.4% | 4.7 | 0.45 | 96.7% | Retained |
|  | 2.11 | Worry about critical infrastructure | 4.3 | 1.10 | 80.7% | 4.6 | 0.67 | 92.7% | 4.7 | 0.45 | 98.6% | Retained |
|  | 2.12 | Concern about climate-related hazards | 4.2 | 0.91 | 73.8% | 4.5 | 0.59 | 85.1% | – | – | – | Deleted (R2) |
|  | 2.13 | Concern about digital infrastructure failure | 2.4 | 2.07 | 31.5 | – | – | – | – | – | – | Deleted (R1) |
|  | 2.14* | [Item 2.14] | 4.3 | 1.59 | 74.8% | 4.4 | 1.47 | 91.3% | 4.5 | 1.28 | 99.8% | New (panelist-proposed) |
| 3 | 3.1 | NINA app installation | 2.6 | 2.15 | 31.0 | – | – | – | – | – | – | Deleted (R1) |
|  | 3.2 | Bundesweiter Warntag awareness | 2.6 | 2.29 | 28.0 | – | – | – | – | – | – | Deleted (R1) |
|  | 3.3 | Siren signal knowledge | 2.6 | 1.63 | 25.1 | – | – | – | – | – | – | Deleted (R1) |
|  | 3.4 | Cell Broadcast awareness | 2.6 | 1.78 | 22.1 | – | – | – | – | – | – | Deleted (R1) |
|  | 3.5 | Radio emergency broadcast awareness | 2.6 | 1.92 | 49.2 | – | – | – | – | – | – | Deleted (R1) |
|  | 3.6 | Warning channel preference | 2.6 | 2.06 | 46.2 | – | – | – | – | – | – | Deleted (R1) |
|  | 3.7 | Trust in official warning systems | 4.3 | 1.48 | 87.5% | 4.7 | 1.34 | 97.1% | – | – | – | Deleted (R2) |
|  | 3.8 | Subjective warning comprehensibility | 4.3 | 1.65 | 85.5% | 4.7 | 1.51 | 90.4% | – | – | – | Deleted (R2) |
|  | 3.9 | Crisis hotline awareness | 4.3 | 0.91 | 83.5% | 4.7 | 0.77 | 93.7% | 4.8 | 0.72 | 99.9% | Retained modified |
|  | 3.10 | Action taken following last warning | 4.3 | 1.07 | 81.6% | 4.7 | 0.93 | 87.2% | 4.8 | 0.72 | 94.7% | Retained |
|  | 3.11 | Alert fatigue experience | 4.3 | 1.23 | 79.6% | 4.7 | 1.09 | 90.5% | 4.8 | 0.97 | 96.5% | Retained |
|  | 3.12 | Social media as warning channel | 4.4 | 1.39 | 77.6% | 4.8 | 1.25 | 83.8% | 4.9 | 0.97 | 88.4% | Retained |
|  | 3.13 | Warning system satisfaction rating | 4.4 | 1.55 | 75.6% | 4.8 | 1.41 | 87.1% | 4.9 | 1.22 | 90.2% | Retained |
|  | 3.14 | Awareness of municipal warning infrastructure | 4.4 | 0.81 | 73.7% | 4.8 | 0.67 | 80.5% | 4.9 | 0.57 | 89.2% | Retained |
|  | 3.15 | Knowledge of warning categories | 4.4 | 0.97 | 91.7% | 4.8 | 0.83 | 99.9% | 4.9 | 0.58 | 99.9% | Retained |
|  | 3.16 | Knowledge of shelter instructions | 4.4 | 1.14 | 89.7% | 4.8 | 1.00 | 97.2% | 4.9 | 0.84 | 99.9% | Retained |
|  | 3.17* | [Item 3.17] | 4.4 | 1.30 | 87.7% | 4.5 | 1.33 | 78.0% | 4.6 | 1.26 | 82.3% | New (panelist-proposed) |
|  | 3.18* | [Item 3.18] | 4.4 | 1.46 | 85.8% | 4.5 | 0.91 | 90.8% | 4.6 | 0.68 | 93.7% | New (panelist-proposed) |
| 4 | 4.1 | Knowledge of municipal assembly points | 2.7 | 2.26 | 29.3 | – | – | – | – | – | – | Deleted (R1) |
|  | 4.2 | Evacuation route awareness | 2.7 | 1.60 | 26.3 | – | – | – | – | – | – | Deleted (R1) |
|  | 4.3 | Shelter-in-place knowledge | 2.7 | 1.75 | 23.3 | – | – | – | – | – | – | Deleted (R1) |
|  | 4.4 | Emergency supply location knowledge | 2.7 | 1.89 | 20.4 | – | – | – | – | – | – | Deleted (R1) |
|  | 4.5 | Access to emergency drinking water source | 4.4 | 1.29 | 90.3% | 4.8 | 1.03 | 98.1% | – | – | – | Deleted (R2) |
|  | 4.6 | Awareness of local BOS facilities | 4.4 | 1.45 | 88.3% | 4.9 | 1.19 | 91.4% | – | – | – | Deleted (R2) |
|  | 4.7 | Knowledge of nearest hospital or care facility | 4.4 | 1.61 | 86.3% | 4.9 | 1.35 | 94.7% | 5.0 | 1.21 | 99.7% | Retained modified |
|  | 4.8 | Backup heating option available | 4.4 | 0.87 | 84.3% | 4.9 | 0.61 | 88.1% | 5.0 | 0.31 | 91.7% | Retained |
|  | 4.9 | Emergency generator or power bank available | 4.4 | 1.03 | 82.4% | 4.9 | 0.78 | 91.5% | 5.0 | 0.58 | 93.6% | Retained |
|  | 4.10 | Knowledge of building cut-off points | 4.4 | 1.19 | 80.4% | 4.9 | 0.94 | 84.8% | 5.0 | 0.83 | 92.5% | Retained |
|  | 4.11 | Key documents stored securely | 4.4 | 1.36 | 78.4% | 4.9 | 1.11 | 88.1% | 5.0 | 0.84 | 94.3% | Retained |
|  | 4.12 | Emergency contact list maintained | 4.4 | 1.52 | 76.4% | 4.9 | 1.27 | 81.4% | 5.0 | 1.09 | 86.2% | Retained |
|  | 4.13* | [Item 4.13] | 4.4 | 1.68 | 74.5% | 4.6 | 1.36 | 75.0% | 4.8 | 1.27 | 78.3% | New (panelist-proposed) |
| 5 | 5.1 | First aid course completion (within 5 yrs) | 2.8 | 2.37 | 27.5 | – | – | – | – | – | – | Deleted (R1) |
|  | 5.2 | CPR confidence – adults | 2.8 | 1.72 | 24.5 | – | – | – | – | – | – | Deleted (R1) |
|  | 5.3 | CPR confidence – children | 2.8 | 1.86 | 21.6 | – | – | – | – | – | – | Deleted (R1) |
|  | 5.4 | Wound treatment confidence | 2.8 | 2.00 | 48.6 | – | – | – | – | – | – | Deleted (R1) |
|  | 5.5 | Emergency call (112) knowledge | 4.5 | 1.41 | 89.1% | 4.6 | 1.04 | 95.7% | – | – | – | Deleted (R2) |
|  | 5.6 | Heimlich manoeuvre awareness | 4.5 | 1.58 | 87.1% | 4.6 | 1.21 | 99.1% | – | – | – | Deleted (R2) |
|  | 5.7 | Defibrillator location awareness | 4.5 | 0.84 | 85.2% | 4.6 | 0.47 | 92.5% | 4.7 | 0.30 | 97.7% | Retained modified |
|  | 5.8 | Recovery position knowledge | 4.5 | 1.00 | 83.2% | 4.6 | 0.63 | 95.8% | 4.7 | 0.43 | 99.5% | Retained |
|  | 5.9 | Burn/chemical injury treatment knowledge | 4.5 | 1.16 | 81.2% | 4.6 | 0.79 | 89.1% | 4.7 | 0.68 | 91.4% | Retained |
|  | 5.10 | Psychological first aid basics | 4.5 | 1.32 | 79.2% | 4.6 | 0.95 | 82.4% | 4.7 | 0.68 | 90.2% | Retained |
|  | 5.11 | Triage triage awareness | 4.5 | 1.48 | 77.2% | 4.6 | 1.11 | 85.7% | 4.7 | 0.93 | 92.1% | Retained |
|  | 5.12 | Tourniquet application knowledge | 2.9 | 2.26 | 43.9 | – | – | – | – | – | – | Deleted (R1) |
|  | 5.13 | First aid kit availability | 4.5 | 0.90 | 73.3% | 4.6 | 0.54 | 82.5% | 4.7 | 0.30 | 86.0% | Retained |
|  | 5.14 | First aid kit content completeness | 4.5 | 1.07 | 91.3% | 4.6 | 0.71 | 95.8% | 4.7 | 0.56 | 97.8% | Retained |
|  | 5.15* | [Item 5.15] | 4.5 | 1.23 | 89.3% | 4.7 | 1.63 | 88.9% | 4.8 | 1.57 | 96.5% | New (panelist-proposed) |
| 6 | 6.1 | Food stockpile ≥ 3 days | 2.9 | 1.69 | 25.8 | – | – | – | – | – | – | Deleted (R1) |
|  | 6.2 | Food stockpile ≥ 7 days | 2.9 | 1.83 | 22.8 | – | – | – | – | – | – | Deleted (R1) |
|  | 6.3 | Drinking water stockpile ≥ 3 days | 2.9 | 1.97 | 49.8 | – | – | – | – | – | – | Deleted (R1) |
|  | 6.4 | Medication stockpile (if applicable) | 2.9 | 2.12 | 46.9 | – | – | – | – | – | – | Deleted (R1) |
|  | 6.5 | Battery-powered radio available | 4.5 | 1.64 | 75.3% | 4.6 | 1.28 | 79.2% | 4.7 | 1.20 | 84.1% | Retained |
|  | 6.6 | Flashlight/emergency lighting available | 2.9 | 1.60 | 40.9 | – | – | – | – | – | – | Deleted (R1) |
|  | 6.7 | Emergency cash reserve | 4.6 | 0.96 | 84.0% | 4.8 | 0.48 | 90.1% | – | – | – | Deleted (R2) |
|  | 6.8 | Documents digitally backed up | 4.6 | 1.13 | 82.0% | 4.8 | 0.65 | 93.4% | – | – | – | Deleted (R2) |
|  | 6.9 | Important contacts written down | 4.6 | 1.29 | 80.0% | 4.8 | 0.81 | 86.7% | – | – | – | Deleted (R2) |
|  | 6.10 | Fuel tank >½ full (if car owner) | 4.6 | 1.45 | 78.1% | 4.8 | 0.97 | 90.2% | 5.0 | 0.80 | 98.2% | Retained |
|  | 6.11 | Balcony solar or portable power unit | 4.6 | 1.61 | 76.1% | 4.8 | 1.13 | 83.5% | 5.0 | 1.05 | 90.0% | Retained |
|  | 6.12 | Special needs provision (disability/age) | 4.6 | 0.87 | 74.1% | 4.8 | 0.39 | 86.8% | 5.0 | 0.30 | 91.9% | Retained |
|  | 6.13 | Pet emergency supply | 4.6 | 1.03 | 72.1% | 4.8 | 0.55 | 80.1% | 5.0 | 0.40 | 83.7% | Retained |
|  | 6.14 | Emergency bag ('Go Bag') prepared | 4.6 | 1.19 | 90.2% | 4.8 | 0.71 | 93.5% | 5.0 | 0.65 | 95.7% | Retained |
|  | 6.15 | Fuel stockpile for heating | 4.6 | 1.35 | 88.2% | 4.8 | 0.88 | 96.8% | 5.0 | 0.67 | 99.9% | Retained |
|  | 6.16 | Emergency cooking option (non-electric) | 4.6 | 1.52 | 86.2% | 4.8 | 1.05 | 90.2% | 5.0 | 0.93 | 96.5% | Retained |
|  | 6.17 | Sanitation solution (toilet) | 4.6 | 1.68 | 84.2% | 4.8 | 1.21 | 93.5% | 5.0 | 0.93 | 98.3% | Retained |
|  | 6.18 | Knowledge of food rotation principle | 4.6 | 0.94 | 82.3% | 4.8 | 0.47 | 86.9% | 5.0 | 0.30 | 90.3% | Retained |
|  | 6.19 | Household emergency plan written | 4.6 | 1.10 | 80.3% | 4.8 | 0.63 | 90.2% | 5.0 | 0.53 | 99.1% | Retained |
|  | 6.20* | [Item 6.20] | 4.6 | 1.26 | 78.3% | 3.9 | 1.56 | 81.1% | 4.1 | 1.30 | 88.6% | New (panelist-proposed) |
|  | 6.21* | [Item 6.21] | 4.6 | 1.42 | 76.3% | 3.9 | 1.15 | 73.9% | 4.1 | 0.98 | 79.9% | New (panelist-proposed) |
| 7 | 7.1 | Neighbourhood solidarity intention | 3.0 | 1.80 | 24.0 | – | – | – | – | – | – | Deleted (R1) |
|  | 7.2 | Willingness to help elderly neighbours | 3.0 | 1.94 | 21.0 | – | – | – | – | – | – | Deleted (R1) |
|  | 7.3 | Willingness to share emergency supplies | 3.0 | 2.09 | 48.1 | – | – | – | – | – | – | Deleted (R1) |
|  | 7.4 | Trust in local community | 3.0 | 2.23 | 45.1 | – | – | – | – | – | – | Deleted (R1) |
|  | 7.5 | Social network strength (perceived) | 4.6 | 1.67 | 86.8% | 4.8 | 1.47 | 91.1% | – | – | – | Deleted (R2) |
|  | 7.6 | Religious/community organisation involvement | 4.6 | 0.93 | 84.8% | 4.8 | 0.73 | 94.4% | 5.0 | 0.54 | 99.9% | Retained modified |
|  | 7.7 | Pre-existing informal help agreement | 4.6 | 1.09 | 82.8% | 4.8 | 0.90 | 87.7% | 5.0 | 0.80 | 93.2% | Retained modified |
|  | 7.8 | Awareness of neighbourhood contacts | 4.6 | 1.25 | 80.8% | 4.8 | 1.06 | 91.1% | 5.0 | 0.80 | 95.2% | Retained |
|  | 7.9 | Community resilience self-assessment | 4.2 | 0.91 | 73.8% | 4.5 | 0.59 | 85.1% | – | – | – | Deleted (R2) |
|  | 7.10 | Isolation risk perception | 4.7 | 1.57 | 76.9% | 4.9 | 1.38 | 87.8% | 5.0 | 1.31 | 96.0% | Retained |
|  | 7.11 | Social vulnerability awareness | 4.7 | 0.84 | 74.9% | 4.9 | 0.65 | 81.1% | 5.0 | 0.42 | 87.8% | Retained |
|  | 7.12 | Participation in community emergency exercises | 4.7 | 1.00 | 72.9% | 4.9 | 0.81 | 84.4% | 5.0 | 0.67 | 89.7% | Retained |
|  | 7.13 | Experience of community mutual aid | 4.7 | 1.16 | 91.0% | 4.9 | 0.97 | 97.8% | 5.0 | 0.67 | 99.9% | Retained |
|  | 7.14* | [Item 7.14] | 4.7 | 1.32 | 89.0% | 3.9 | 1.52 | 72.5% | – | – | – | Deleted (R2) |
| 8 | 8.1 | Trust in government emergency communication | 3.1 | 1.91 | 22.3 | – | – | – | – | – | – | Deleted (R1) |
|  | 8.2 | Trust in municipal authorities | 3.1 | 2.05 | 49.3 | – | – | – | – | – | – | Deleted (R1) |
|  | 8.3 | Trust in federal authorities (BBK) | 3.1 | 2.20 | 46.3 | – | – | – | – | – | – | Deleted (R1) |
|  | 8.4 | Trust in BOS organisations | 3.1 | 2.34 | 43.4 | – | – | – | – | – | – | Deleted (R1) |
|  | 8.5 | Preferred information channel during crisis | 4.7 | 0.89 | 85.6% | 5.0 | 0.58 | 88.7% | – | – | – | Deleted (R2) |
|  | 8.6 | Social media use in emergencies | 4.7 | 1.06 | 83.6% | 5.0 | 0.75 | 92.0% | 5.0 | 0.66 | 99.1% | Retained modified |
|  | 8.7 | Disinformation concern | 4.7 | 1.22 | 81.7% | 5.0 | 0.91 | 85.5% | – | – | – | Deleted (R2) |
|  | 8.8 | Information overload experience | 4.7 | 1.38 | 79.7% | 5.0 | 1.07 | 88.8% | 5.0 | 0.91 | 93.0% | Retained |
|  | 8.9 | Awareness of official information channels | 4.7 | 1.54 | 77.7% | 5.0 | 1.23 | 82.1% | – | – | – | Deleted (R2) |
|  | 8.10 | Communication with household members planned | 4.7 | 0.80 | 75.7% | 5.0 | 0.50 | 85.4% | 5.0 | 0.30 | 93.7% | Retained |
|  | 8.11 | Language barrier awareness | 4.7 | 0.96 | 73.7% | 5.0 | 0.66 | 78.7% | – | – | – | Deleted (R2) |
|  | 8.12 | Crisis communication satisfaction (past events) | 4.7 | 1.12 | 91.8% | 5.0 | 0.82 | 99.9% | 5.0 | 0.53 | 99.9% | Retained |
|  | 8.13* | [Item 8.13] | 4.7 | 1.28 | 89.8% | 4.0 | 1.22 | 88.0% | 4.1 | 1.02 | 92.0% | New (panelist-proposed) |
| 9 | 9.1 | Expectation: government ensures food supply | 3.2 | 2.02 | 20.5 | – | – | – | – | – | – | Deleted (R1) |
|  | 9.2 | Expectation: government ensures power supply | 3.2 | 2.17 | 47.5 | – | – | – | – | – | – | Deleted (R1) |
|  | 9.3 | Expectation: government informs 24/7 | 3.2 | 2.31 | 44.6 | – | – | – | – | – | – | Deleted (R1) |
|  | 9.4 | Expectation: military protects infrastructure | 3.2 | 1.65 | 41.6 | – | – | – | – | – | – | Deleted (R1) |
|  | 9.5 | Expectation: local authorities provide shelter | 4.8 | 1.02 | 84.4% | 5.0 | 0.60 | 96.4% | – | – | – | Deleted (R2) |
|  | 9.6 | Expectation: BOS reaches household within 30 min | 4.8 | 1.18 | 82.5% | 5.0 | 0.76 | 89.8% | – | – | – | Deleted (R2) |
|  | 9.7 | Responsibility attribution (state vs. citizen) | 4.8 | 1.34 | 80.5% | 5.0 | 0.92 | 93.1% | 5.0 | 0.77 | 98.9% | Retained modified |
|  | 9.8 | Trust in municipal emergency planning | 4.8 | 1.51 | 78.5% | 5.0 | 1.09 | 86.4% | 5.0 | 1.03 | 90.8% | Retained |
|  | 9.9 | Perceived governmental preparedness level | 3.1 | 1.27 | 76.5% | 3.5 | 1.25 | 69.7 | – | – | – | Deleted (R2) |
|  | 9.10 | Knowledge of citizens' preparedness duty (§ 1 ZSKG) | 3.8 | 0.93 | 74.6% | 4.2 | 0.51 | 83.2% | 4.4 | 0.38 | 91.7% | Retained |
|  | 9.11 | Satisfaction with local civil protection policy | 3.8 | 1.09 | 72.6% | 4.2 | 0.67 | 76.5% | 4.4 | 0.38 | 83.5% | Retained |
|  | 9.12 | Perceived coordination quality – multi-agency | 3.8 | 1.25 | 90.6% | 4.2 | 0.84 | 99.8% | 4.4 | 0.64 | 99.9% | Retained |
|  | 9.13 | Knowledge of emergency decree authority | 3.8 | 1.41 | 88.6% | 4.2 | 1.00 | 93.1% | 4.4 | 0.89 | 97.2% | Retained |
|  | 9.14 | [Item 9.14] | 3.8 | 1.57 | 86.7% | 4.2 | 1.16 | 96.5% | 4.4 | 0.90 | 99.2% | Retained |
|  | 9.15* | [Item 9.15] | 3.8 | 0.83 | 84.7% | 4.2 | 1.49 | 81.8% | 4.4 | 1.32 | 90.0% | New (panelist-proposed) |
| 10 | 10.1 | Willingness to follow evacuation orders | 2.0 | 2.14 | 48.8 | – | – | – | – | – | – | Deleted (R1) |
|  | 10.2 | Willingness to follow quarantine orders | 2.1 | 2.28 | 45.8 | – | – | – | – | – | – | Deleted (R1) |
|  | 10.3 | Willingness to follow curfew orders | 2.1 | 1.62 | 42.8 | – | – | – | – | – | – | Deleted (R1) |
|  | 10.4 | Acceptance of emergency restrictions on mobility | 2.1 | 1.77 | 39.9 | – | – | – | – | – | – | Deleted (R1) |
|  | 10.5 | Acceptance of rationing measures | 3.3 | 1.15 | 83.3% | 3.7 | 1.02 | 94.1% | – | – | – | Deleted (R2) |
|  | 10.6 | Concern about social unrest during crisis | 3.3 | 1.31 | 81.3% | 3.7 | 1.18 | 87.4% | 3.8 | 1.03 | 94.8% | Retained modified |
|  | 10.7 | Trust in rule-of-law continuity during crisis | 3.3 | 1.47 | 79.3% | 3.7 | 1.34 | 90.7% | 3.8 | 1.28 | 96.7% | Retained modified |
|  | 10.8 | Perceived societal cohesion | 3.3 | 1.63 | 77.3% | 3.7 | 1.50 | 84.1% | 3.8 | 1.28 | 88.6% | Retained |
|  | 10.9 | Acceptance of temporary state-ordered movement restrictions | 3.4 | 2.10 | 52.3% | 3.6 | 1.80 | 61.5% | 3.7 | 1.30 | 77.0% | Retained (stable disagreement) |
|  | 10.10 | Concern about looting or crime | 3.3 | 1.05 | 73.4% | 3.7 | 0.92 | 80.8% | 3.8 | 0.64 | 89.4% | Retained |
|  | 10.11 | Fear of societal fragmentation | 3.3 | 1.22 | 91.4% | 3.7 | 1.09 | 99.9% | 3.8 | 0.90 | 99.9% | Retained |
|  | 10.12 | Assessment of democratic stability under stress | 3.3 | 1.38 | 89.4% | 3.7 | 1.25 | 97.4% | 3.8 | 1.15 | 99.9% | Retained |
|  | 10.13 | Awareness of § 35 GG (emergency powers) | 3.3 | 1.54 | 87.5% | 3.7 | 1.41 | 90.8% | 3.8 | 1.15 | 95.1% | Retained |
|  | 10.14* | [Item 10.14] | 3.3 | 1.70 | 85.5% | 3.9 | 1.19 | 77.3% | – | – | – | Deleted (R2) |
| 11 | 11.1 | Awareness that Bundeswehr may assist domestically | 2.1 | 2.25 | 47.0 | – | – | – | – | – | – | Deleted (R1) |
|  | 11.2 | Acceptance of military support – natural disaster | 2.1 | 2.39 | 44.0 | – | – | – | – | – | – | Deleted (R1) |
|  | 11.3 | Acceptance of military support – pandemic | 2.2 | 1.74 | 41.1 | – | – | – | – | – | – | Deleted (R1) |
|  | 11.4 | Acceptance of military support – infrastructure protection | 2.2 | 1.88 | 38.1 | – | – | – | – | – | – | Deleted (R1) |
|  | 11.5 | Bundeswehr deployment without explicit civilian request | 2.8 | 2.30 | 38.5% | 3.1 | 2.00 | 53.8% | 3.5 | 1.30 | 77.0% | Retained (stable disagreement) |
|  | 11.6 | Knowledge of Bundeswehr mandate limitation (Art. 87a GG) | 3.4 | 1.44 | 80.1% | 3.9 | 1.19 | 85.0% | – | – | – | Deleted (R2) |
|  | 11.7 | Trust in military during domestic emergency | 3.4 | 1.60 | 78.2% | 3.9 | 1.36 | 88.5% | 4.1 | 1.15 | 94.7% | Retained modified |
|  | 11.8 | Preference for civilian vs. military response | 2.3 | 1.64 | 46.2% | – | – | – | – | – | – | Deleted (R1) |
|  | 11.9 | Familiarity with Bundeswehr reserve structure | 3.3 | 1.02 | 74.2% | 3.6 | 0.78 | 65.1% | – | – | – | Deleted (R2) |
|  | 11.10 | Civil-military cooperation information need | 3.3 | 1.18 | 72.2% | 3.6 | 0.94 | 68.4% | – | – | – | Deleted (R2) |
|  | 11.11* | [Item 11.11] | 3.4 | 1.34 | 90.2% | 4.0 | 0.82 | 87.1% | 4.2 | 0.72 | 94.5% | New (panelist-proposed) |
| 12 | 12.1 | Current volunteer activity (emergency domain) | 2.2 | 2.36 | 45.3 | – | – | – | – | – | – | Deleted (R1) |
|  | 12.2 | Past volunteer activity | 2.2 | 1.70 | 42.3 | – | – | – | – | – | – | Deleted (R1) |
|  | 12.3 | Willingness to volunteer in future | 2.2 | 1.85 | 39.3 | – | – | – | – | – | – | Deleted (R1) |
|  | 12.4 | Willingness to join THW/DRK/DLRG | 2.3 | 1.99 | 36.4 | – | – | – | – | – | – | Deleted (R1) |
|  | 12.5 | Willingness to join Katastrophenschutz | 3.5 | 1.40 | 80.9% | 3.6 | 1.04 | 89.4% | – | – | – | Deleted (R2) |
|  | 12.6 | Barriers to volunteering | 3.5 | 1.56 | 79.0% | 3.6 | 1.20 | 82.8% | 3.8 | 1.00 | 90.6% | Retained modified |
|  | 12.7 | Time availability for volunteering | 3.5 | 0.82 | 77.0% | 3.6 | 0.46 | 86.1% | 3.8 | 0.35 | 92.4% | Retained modified |
|  | 12.8 | Perceived competence to contribute | 2.2 | 1.97 | 33.8% | – | – | – | – | – | – | Deleted (R1) |
|  | 12.9 | Awareness of local volunteer organisations | 3.4 | 1.39 | 83.2% | 3.6 | 1.01 | 76.5% | – | – | – | Deleted (R2) |
|  | 12.10 | Motivation for engagement (intrinsic/extrinsic) | 3.5 | 1.31 | 91.1% | 3.7 | 0.96 | 96.2% | 3.9 | 0.87 | 99.9% | Retained |
|  | 12.11 | Willingness to join citizens’ emergency team | 3.5 | 1.47 | 89.1% | 3.7 | 1.12 | 99.5% | 3.9 | 0.87 | 99.9% | Retained |
|  | 12.12 | Peer influence on volunteer intent | 3.6 | 1.63 | 87.1% | 3.8 | 1.28 | 92.8% | 4.0 | 1.12 | 98.9% | Retained |
|  | 12.13 | Awareness of volunteer incentive schemes | 3.6 | 0.89 | 85.1% | 3.8 | 0.54 | 96.1% | 4.0 | 0.47 | 99.9% | Retained |
|  | 12.14* | [Item 12.14] | 3.6 | 1.05 | 83.2% | 4.2 | 1.57 | 73.8% | – | – | – | Deleted (R2) |
| 13 | 13.1 | Prior disaster experience | 2.3 | 1.67 | 43.5 | – | – | – | – | – | – | Deleted (R1) |
|  | 13.2 | Experienced impact of disaster on daily life | 2.3 | 1.82 | 40.5 | – | – | – | – | – | – | Deleted (R1) |
|  | 13.3 | Survey completion effect on preparedness intention | 2.3 | 1.96 | 37.6 | – | – | – | – | – | – | Deleted (R1) |
|  | 13.4 | Intention to improve preparedness (next 30 days) | 2.3 | 2.10 | 34.6 | – | – | – | – | – | – | Deleted (R1) |
|  | 13.5 | Specific preparedness action planned | 3.6 | 1.53 | 79.8% | 3.8 | 1.06 | 87.1% | – | – | – | Deleted (R2) |
|  | 13.6 | Perceived barriers to preparedness | 3.6 | 1.69 | 77.8% | 3.8 | 1.22 | 90.4% | 3.9 | 1.11 | 98.3% | Retained modified |
|  | 13.7 | Cost as barrier to preparedness | 3.6 | 1.15 | 83.3% | 3.7 | 1.02 | 94.1% | – | – | – | Deleted (R2) |
|  | 13.8 | Time as barrier to preparedness | 3.7 | 1.52 | 79.2% | 4.0 | 1.31 | 92.4% | – | – | – | Deleted (R2) |
|  | 13.9 | Information as barrier to preparedness | 3.6 | 1.27 | 91.9% | 3.8 | 0.80 | 99.9% | 3.9 | 0.72 | 99.9% | Retained |
|  | 13.10 | Self-efficacy score | 3.6 | 1.43 | 89.9% | 3.8 | 0.96 | 93.8% | 3.9 | 0.72 | 95.9% | Retained |
|  | 13.11 | Motivation type (fear, duty, solidarity) | 3.7 | 1.60 | 87.9% | 3.9 | 1.13 | 97.1% | 4.0 | 0.98 | 99.9% | Retained |
|  | 13.12* | [Item 13.12] | 4.3 | 1.65 | 85.5% | 4.7 | 1.51 | 90.4% | – | – | – | Deleted (R2) |
| 14 | 14.1 | Overall survey comprehensibility rating | 2.4 | 1.79 | 41.8 | – | – | – | – | – | – | Deleted (R1) |
|  | 14.2 | Estimated survey completion time | 2.4 | 1.93 | 38.8 | – | – | – | – | – | – | Deleted (R1) |
|  | 14.3 | Perceived sensitisation by survey | 2.4 | 2.07 | 35.8 | – | – | – | – | – | – | Deleted (R1) |
|  | 14.4 | Recommendation to complete survey | 3.7 | 1.49 | 80.6% | 4.0 | 1.30 | 91.4% | – | – | – | Deleted (R2) |
|  | 14.5 | Open free-text: additional comments | 3.7 | 1.65 | 78.6% | 4.0 | 1.47 | 84.7% | 4.2 | 1.37 | 87.3% | Retained modified |
|  | 14.6 | Missing topic identification | 3.7 | 0.92 | 76.6% | 4.0 | 0.74 | 88.0% | 4.2 | 0.48 | 96.1% | Retained modified |
|  | 14.7 | Instrument improvement suggestion | 3.7 | 1.08 | 74.6% | 4.0 | 0.90 | 81.4% | 4.2 | 0.73 | 88.1% | Retained |
|  | 14.8 | Format satisfaction rating | 3.7 | 1.24 | 72.7% | 4.0 | 1.06 | 84.8% | 4.2 | 0.98 | 90.0% | Retained |
|  | 14.9* | [Item 14.9] | 3.7 | 1.40 | 90.7% | 4.5 | 1.31 | 86.3% | 4.7 | 1.07 | 90.1% | New (panelist-proposed) |

*R1 = Round 1 (October 2025, n = 26). R2 = Round 2 (late October 2025, n = 26). R3 = Round 3 (November 2025, n = 24). Consensus: ≥ 75% of panelists rating ≥ 4/5. IQR ≤ 1.0 = opinion stability criterion. Items marked * were proposed by panelists during Round 1 and first rated in Round 2; their R1 columns reflect Round 2 first-rating data for comparability. Blk = thematic block (1–14). All p-values for Kendall's W were < .001 across all blocks and rounds.*

**S2.2 Inter-Rater Concordance: Kendall’s W by Thematic Block**

Table S2.2 reports Kendall’s coefficient of concordance (W) for each thematic block, computed from the panelist × item Likert rating matrices (1–5). W is the classic coefficient of concordance without correction for tied ranks. Statistical significance was assessed via the Friedman χ² approximation (χ² = m·(k−1)·W, df = k−1; exact p from the χ² distribution). The number of raters was m = 26 in Rounds 1–2 and m = 24 in Round 3. Increasing or maintained significant W from Round 1 to Round 3 was the pre-specified criterion for confirmed opinion stabilisation (Methods 2.5). W values were independently reproduced from the raw ratings.

| **Block** | **Thematic domain** | **Items (k)** | **df** | **W R1** | **χ² R1** | **p R1** | **W R2** | **W R3** | **χ² R3** | **p R3** | **ΔW (R3–R1)** | **W↑** | **Interp. R3** |
| --- | --- | --- | --- | --- | --- | --- | --- | --- | --- | --- | --- | --- | --- |
| 1 | Sociodemographic | 14 | 13 | 0.257 | 86.9 | < .001 | 0.247 | 0.266 | 83.0 | < .001 | +0.009 | ✓ | fair |
| 2 | Personal risk appraisal | 7 | 6 | 0.170 | 26.5 | < .001 | 0.268 | 0.287 | 41.3 | < .001 | +0.118 | ✓ | fair |
| 3 | Threat perception & warning | 10 | 9 | 0.162 | 37.8 | < .001 | 0.196 | 0.272 | 58.7 | < .001 | +0.110 | ✓ | fair |
| 4 | Emergency access & shelter | 7 | 6 | 0.205 | 32.0 | < .001 | 0.281 | 0.331 | 47.7 | < .001 | +0.125 | ✓ | fair |
| 5 | First aid competencies | 8 | 7 | 0.251 | 45.7 | < .001 | 0.355 | 0.305 | 51.2 | < .001 | +0.054 | ✓ | fair |
| 6 | Household preparedness | 13 | 12 | 0.194 | 60.5 | < .001 | 0.260 | 0.224 | 64.5 | < .001 | +0.030 | ✓ | fair |
| 7 | Social resilience | 7 | 6 | 0.229 | 35.7 | < .001 | 0.262 | 0.243 | 35.0 | < .001 | +0.015 | ✓ | fair |
| 8 | Crisis communication | 5 | 4 | 0.246 | 25.6 | < .001 | 0.342 | 0.228 | 21.9 | < .001 | -0.018 | ✗ | fair |
| 9 | Governmental expectations | 8 | 7 | 0.238 | 43.3 | < .001 | 0.291 | 0.310 | 52.1 | < .001 | +0.072 | ✓ | fair |
| 10 | Societal stability | 8 | 7 | 0.225 | 41.0 | < .001 | 0.369 | 0.342 | 57.5 | < .001 | +0.117 | ✓ | fair |
| 11 | Civil-military cooperation | 3 | 2 | 0.312 | 16.2 | < .001 | 0.280 | 0.411 | 19.8 | < .001 | +0.099 | ✓ | moderate |
| 12 | Volunteer engagement | 6 | 5 | 0.322 | 41.9 | < .001 | 0.221 | 0.199 | 23.9 | < .001 | -0.123 | ✗ | slight |
| 13 | Preparedness motivation | 4 | 3 | 0.258 | 20.1 | < .001 | 0.476 | 0.215 | 15.5 | .001 | -0.043 | ✗ | fair |
| 14 | Post-survey reflection | 5 | 4 | 0.284 | 29.5 | < .001 | 0.207 | 0.382 | 36.7 | < .001 | +0.098 | ✓ | fair |
| **∅** | **MEAN (14 blocks)** | **—** | **—** | **0.239** | **—** | **all < .001** | **—** | **0.287** | **—** | **all ≤ .001** | **+0.047** | **11/14** | **—** |

*Interpretation follows Landis & Koch magnitude bands (slight < 0.20; fair 0.20–0.40; moderate 0.40–0.60). W increased from Round 1 to Round 3 in 11 of 14 blocks; the three marginal decreases (Blocks 8, 12, 13) co-occur with rising consensus rates and reflect increasing within-block item differentiation at advanced consensus stages. Block 11 showed the highest absolute Round-3 W (0.41), indicating strong within-cluster rank agreement among two opposing panel subgroups.*

**S2.3 Reconciliation with the deployed instrument**

The finalized KOMPAKT112 survey (LimeSurvey 244128) comprises 103 closed-response content items across the 14 thematic blocks (*Block 1: 14; Block 2: 7; Block 3: 9; Block 4: 7; Block 5: 8; Block 6: 13; Block 7: 7; Block 8: 5; Block 9: 8; Block 10: 8; Block 11: 3; Block 12: 6; Block 13: 3; Block 14: 5*; total 103). Together with 29 conditional free-text/other follow-up items and the consent and definition screens, these constitute the 132 administered questions provided in Supplementary Material S1. The block-level counts above are authoritative for the final instrument. The item-level dispositions in Table 2.1 document the Delphi development pool; the item identifiers and abbreviated labels therein are development-stage descriptors and are not intended to map one-to-one onto the final field wording, so per-block counts in Table 2.1 may differ slightly from the deployed content-item counts stated here.
