# Supplementary material for: Development and consensus-based validation of a comprehensive population survey instrument for municipal crisis preparedness: a three-round Delphi study (KOMPAKT112)
Source: Front Public Health. 2026 Aug 5;14:1883344. doi: 10.3389/fpubh.2026.1883344 (PMC13485894; doi:10.3389/fpubh.2026.1883344)
Supplement: Supplementary file 3 [file Supplementary_file_3.docx]

**Supplementary Material S3 – CREDES Compliance Checklist**

*Guidance on Conducting and REporting DElphi Studies (Jünger et al., Palliative Medicine 2017; 31(8):684–706)*

The table below documents compliance of the KOMPAKT112 Delphi study with all 29 reporting criteria of the CREDES guideline. All criteria are addressed (29/29). ✓ = criterion fully addressed; the column 'Location in Manuscript / Supplements' identifies the specific section or supplementary file where the criterion is met.

| **Domain** | **CREDES Criterion (Jünger et al. 2017)** | **Addressed** | **Location in Manuscript / Supplements** |
| --- | --- | --- | --- |
| **Background and Rationale** | 1. Rationale for the use of a Delphi approach for the specific research question provided | **✓** | Methods §2.1 |
|  | 2. Description of specific research question addressed by the Delphi study | **✓** | Introduction §1; Abstract |
|  | 3. Review or description of relevant existing literature or evidence | **✓** | Introduction §1; Methods §2.3 |
| **Study Design** | 4. Number of rounds defined a priori | **✓** | Methods §2.1 |
|  | 5. Justification for modification(s) of the standard Delphi design | **✓** | Methods §2.1 |
|  | 6. Piloting of the questionnaire described (if applicable) | **✓** | Methods §2.6; Results §3.5 |
|  | 7. Information provided on the software used for online survey delivery | **✓** | Methods §2.1 |
|  | 8. Statement on ethical approval or confirmation that none required | **✓** | Methods §2.6 |
| **Participants** | 9. Rationale and procedure for expert selection described | **✓** | Methods §2.2 |
|  | 10. Criteria for inclusion and exclusion of panelists defined | **✓** | Methods §2.2 |
|  | 11. Number of experts invited and participating per round reported | **✓** | Results §3.1; Table 1 |
|  | 12. Characteristics of panelists described | **✓** | Results §3.1; Supplementary S4 |
|  | 13. Dropouts between rounds described (with reasons if available) | **✓** | Results §3.1 |
| **Information Sources and Item Development** | 14. Method used to generate initial item pool described | **✓** | Methods §2.3 |
|  | 15. Items derived from systematic literature search and/or expert consultation | **✓** | Methods §2.3 |
|  | 16. Items added during the study (panelist proposals) described | **✓** | Methods §2.4; Results §3.2 |
| **Questions and Items** | 17. Response scale described and justified | **✓** | Methods §2.4 |
|  | 18. Open-ended questions or free-text options described | **✓** | Methods §2.4 |
|  | 19. Full questionnaire published or available on request | **✓** | Supplementary S1 (instrument) & S2 (item-level data) |
| **Consensus Process** | 20. Definition of consensus stated a priori | **✓** | Methods §2.5 |
|  | 21. Level of consensus defined a priori (threshold ≥ 75%) | **✓** | Methods §2.5 |
|  | 22. Feedback provided to panelists described (content and format) | **✓** | Methods §2.4 |
|  | 23. Items not reaching consensus and their handling described | **✓** | Methods §2.5; Results §3.3; Supplementary S5 |
| **Statistics** | 24. Statistical analyses described (median, IQR, Kendall's W, CVI) | **✓** | Methods §2.5; Results §3.2–3.3 |
|  | 25. Data on stability of ratings between rounds presented | **✓** | Results §3.3; Table 3 |
| **Results** | 26. Results reported per round (consensus rates, IQR, W, CVI) | **✓** | Results §3.2–3.7; Tables 2–3 |
|  | 27. Items reaching and not reaching consensus reported per round | **✓** | Results §3.2; Table 2; Supplementary S2 |
| **Discussion and Conclusions** | 28. Limitations of the Delphi study discussed | **✓** | Discussion §4.5 |
|  | 29. Implications and recommendations discussed | **✓** | Discussion §4.1; §4.4; Conclusion §5 |

*Reference: Jünger S, Payne SA, Brine J, Radbruch L, Brearley SG. Guidance on Conducting and REporting DElphi Studies (CREDES) in palliative care: Recommendations based on a methodological systematic review. Palliative Medicine. 2017;31(8):684–706. doi:10.1177/0269216317690685. CREDES was selected as the primary reporting standard because it provides the most comprehensive and widely adopted framework for Delphi studies in health and related sciences (Schifano & Niederberger 2025). All 29 criteria were addressed a priori; no criteria were waived or partially met.*
