# Supplementary material for: Development and consensus-based validation of a comprehensive population survey instrument for municipal crisis preparedness: a three-round Delphi study (KOMPAKT112)
Source: Front Public Health. 2026 Aug 5;14:1883344. doi: 10.3389/fpubh.2026.1883344 (PMC13485894; doi:10.3389/fpubh.2026.1883344)
Supplement: Supplementary file 4 [file Supplementary_file_4.docx]

**Supplementary Material S4 - Expert Panel Characteristics**

*Anonymised profile of all 26 Delphi panelists across 8 professional domains*

Table S4 provides anonymised characteristics of all 26 expert panelists recruited for the KOMPAKT112 Delphi study. Individual identities are not disclosed in accordance with informed consent agreements and GDPR provisions. Panelists are identified by sequential anonymous ID (P01–P26) within professional domain groupings. Domain ordering follows the sequence used in Table 1 of the main manuscript.

| **ID** | **Professional Domain** | **Experience** | **Gender** | **University Degree** | **Operationally Active** | **R1** | **R2** | **R3** |
| --- | --- | --- | --- | --- | --- | --- | --- | --- |
| P01 | Science / Academic | >15 years | M | Yes | Yes | ✓ | ✓ | ✓ |
| P02 | Science / Academic | >15 years | M | Yes | Yes | ✓ | ✓ | ✓ |
| P03 | Science / Academic | >15 years | M | Yes | Yes | ✓ | ✓ | ✓ |
| P04 | Science / Academic | >15 years | F | Yes | No | ✓ | ✓ | ✓ |
| P05 | Fire Brigade / BOS | 11–15 years | M | Yes | Yes | ✓ | ✓ | ✓ |
| P06 | Fire Brigade / BOS | >15 years | F | Yes | No | ✓ | ✓ | ✓ |
| P07 | Fire Brigade / BOS | 11–15 years | M | No | Yes | ✓ | ✓ | ✓ |
| P08 | Fire Brigade / BOS | >15 years | M | Yes | Yes | ✓ | ✓ | ✓ |
| P09 | Technical Relief / THW | 11–15 years | M | Yes | Yes | ✓ | ✓ | ✓ |
| P10 | Technical Relief / THW | >15 years | F | No | Yes | ✓ | ✓ | ✓ |
| P11 | Aid Organisations | 5–10 years | M | Yes | Yes | ✓ | ✓ | ✓ |
| P12 | Aid Organisations | >15 years | M | Yes | Yes | ✓ | ✓ | ✓ |
| P13 | Aid Organisations | 11–15 years | F | No | Yes | ✓ | ✓ | ✓ |
| P14 | Aid Organisations | >15 years | M | Yes | No | ✓ | ✓ | – |
| P15 | Aid Organisations | 11–15 years | M | Yes | Yes | ✓ | ✓ | ✓ |
| P16 | Bundeswehr | 11–15 years | M | No | Yes | ✓ | ✓ | ✓ |
| P17 | Bundeswehr | >15 years | F | Yes | No | ✓ | ✓ | ✓ |
| P18 | Bundeswehr | >15 years | M | Yes | Yes | ✓ | ✓ | ✓ |
| P19 | Bundeswehr | 11–15 years | M | No | Yes | ✓ | ✓ | ✓ |
| P20 | Church / Civil Society | >15 years | M | Yes | No | ✓ | ✓ | ✓ |
| P21 | Church / Civil Society | 5–10 years | F | No | Yes | ✓ | ✓ | ✓ |
| P22 | Church / Civil Society | 11–15 years | M | Yes | Yes | ✓ | ✓ | ✓ |
| P23 | Municipal Administration / CP | >15 years | M | Yes | Yes | ✓ | ✓ | – |
| P24 | Municipal Administration / CP | <5 years | M | No | Yes | ✓ | ✓ | ✓ |
| P25 | Municipal Administration / CP | >15 years | F | Yes | No | ✓ | ✓ | ✓ |
| P26 | Communications / PR | 11–15 years | M | No | Yes | ✓ | ✓ | ✓ |
| **TOTAL (n = 26)** | |  | 18 M / 7 F / 1 D | 16/26 (61.5%) | 14/26 (53.8%) | 26 | 26 | 24 |

*M = male; F = female; D = diverse. Experience = years of active professional experience in a role relevant to civil protection, emergency management, or related domains. University Degree = bachelor's degree or higher. Operationally Active = currently active as first responder, BOS member, or equivalent. R1/R2/R3 = participation in respective Delphi round (✓ = participated; – = withdrew). Two Aid Organisation panelists (P14, P23) withdrew before Round 3 citing scheduling conflicts; their Round 2 positions were accepted as final per pre-specified protocol. Data collected via online registration form at time of consent, October 2025.*

**Domain distribution and group composition**

| **Domain** | **n** | **% Panel** | **Gender (M/F/D)** | **≥75% with >15 yrs exp.** |
| --- | --- | --- | --- | --- |
| Science / Academic | 4 | 15.4% | 3M/1F | 75% |
| Fire Brigade / BOS | 4 | 15.4% | 3M/1F | 75% |
| Technical Relief / THW | 2 | 7.7% | 2M/0F | 100% |
| Aid Organisations | 5 | 19.2% | 3M/2F | 40% |
| Bundeswehr | 4 | 15.4% | 4M/0F | 75% |
| Church / Civil Society | 3 | 11.5% | 0M/2F/1D | 33% |
| Municipal Administration / CP | 3 | 11.5% | 2M/1F | 67% |
| Communications / PR | 1 | 3.8% | 1M/0F | 100% |
| **TOTAL** | 26 | 100% | 18M / 7F / 1D | 61.5% |
