# Supplementary material for: Development and consensus-based validation of a comprehensive population survey instrument for municipal crisis preparedness: a three-round Delphi study (KOMPAKT112)
Source: Front Public Health. 2026 Aug 5;14:1883344. doi: 10.3389/fpubh.2026.1883344 (PMC13485894; doi:10.3389/fpubh.2026.1883344)
Supplement: Supplementary file 5 [file Supplementary_file_5.docx]

**Supplementary Material S5 – Items with Stable Expert Disagreement**

*Bipolarity analysis and pre-specified decision rules — Blocks 10 and 11*

Two items in the KOMPAKT112 instrument retained IQR > 1.0 in Round 3, indicating that panel consensus on their relevance rating did not fully stabilise. Per the pre-specified decision rules (Methods §2.5), items meeting the following two conditions were retained despite stable disagreement: (1) overall panel median ≥ 3.5 in Round 3; and (2) ≥ 75% internal consensus within at least one domain-specific expert subgroup in Round 3. Both conditions were met for both items. The bipolarity pattern with high relevance ratings from one expert cluster, low ratings from another was confirmed statistically by Kendall's W (all p < .001) and interpreted as substantive, predictable expert disagreement rather than random response variation.

These items are annotated in the KOMPAKT112 instrument (Supplementary S1) with a contextualising note in the block preamble, as recommended by the CREDES standard (Criterion 23; Supplementary S3).

**Block 10 – Item 10.9**

**Item wording (German, original):** *„In einer schwerwiegenden Krise sollte der Staat befugt sein, zeitlich begrenzte Einschränkungen der Bewegungsfreiheit anzuordnen.“ (Block 10: Societal stability)*

**Round-by-round statistics and inter-group pattern**

| **Round** | **Median** | **IQR** | **Cons. % (≥4)** | **Inter-group pattern** |
| --- | --- | --- | --- | --- |
| Round 1 | 3.4 | 2.10 | 52.3% | Strong bipolarity: governmental/municipal subgroup clustered at 5 (median 4.8); church/layperson subgroup clustered at 2 (median 2.1). No single IQR convergence. |
| Round 2 | 3.6 | 1.80 | 61.5% | Marginal shift toward centre; within-cluster distributions tightened but opposing clusters retained. Median rose by 0.2 following structured feedback. |
| Round 3 | 3.7 | 1.30 | 77.0% | Overall consensus of 77.0% (≥75% threshold met); IQR 1.30 exceeds ≤1.0 stability criterion. Bipolarity confirmed by W = 0.41 (all p < .001) with two distinct rank clusters. Retained per pre-specified rule: median ≥ 3.5 ✓ and ≥75% internal consensus in governmental/municipal subgroup ✓. |

**Final decision: RETAINED with annotation**

**Rationale:**

The item captures a legally and ethically contested dimension of societal acceptance of emergency powers — a dimension whose inclusion was specifically advocated by governmental and municipal panelists as operationally relevant for the KOMPAKT112 main survey. Exclusion would introduce systematic blind-spot bias toward lay-population perspectives. The stable disagreement itself constitutes a substantive empirical finding (polarised attitudes toward emergency executive authority) that the main survey instrument should be capable of measuring. Item retained and flagged in Block 10 preamble with explanatory note.

**Block 11 – Item 11.5**

**Item wording (German, original):** *„Im Katastrophenfall sollte die Bundeswehr auch ohne ausdrückliche Anforderung durch zivile Behörden tätig werden dürfen.“ (Block 11: Civil-military cooperation)*

**Round-by-round statistics and inter-group pattern**

| **Round** | **Median** | **IQR** | **Cons. % (≥4)** | **Inter-group pattern** |
| --- | --- | --- | --- | --- |
| Round 1 | 2.8 | 2.30 | 38.5% | Maximum observed bipolarity: Bundeswehr subgroup median 4.9 (all rated 4–5); church/civil society subgroup median 1.5 (all rated 1–2). Lay-population panelists distributed across full scale. |
| Round 2 | 3.1 | 2.00 | 53.8% | Some convergence from moderate panelists toward midpoint; core clusters unchanged. Reformulation of item wording (Bundeswehr mandate clarification added in parentheses) improved clarity ratings but not consensus. |
| Round 3 | 3.5 | 1.30 | 77.0% | IQR 1.30; W = 0.38 (p < .001). Full scale still used by panel. Two clusters clearly identifiable: military-adjacent panelists (≥4) vs. constitutional-constraint-oriented panelists (≤2). Retained per decision rule: median ≥ 3.5 ✓ and ≥75% internal consensus in Bundeswehr subgroup ✓. |

**Final decision: RETAINED with annotation**

**Rationale:**

This item operationalises the key governance tension at the heart of German civil-military cooperation: the constitutional constraint under Art. 87a GG versus the practical need for rapid Bundeswehr engagement in catastrophic scenarios. Stable expert disagreement on this item is a predictable and legitimate reflection of the existing constitutional and operational debate in Germany (Juling 2022; Bollen & Kalkman 2022). Excluding this item would systematically underrepresent the civil-military cooperation dimension that Block 11 was specifically designed to capture. The bipolarity finding is documented in the results (§3.3) and discussed in §4.4 of the main manuscript. Item retained and annotated in Block 11 preamble.

**Expert cluster composition — both items**

The bipolarity pattern across both items reflected the same structural cleavage along the civilian–military/governmental axis:

| **Expert Cluster** | **Domains** | **Typical Rating (R3)** | **Within-cluster W (R3)** |
| --- | --- | --- | --- |
| **Governmental / military cluster** | Bundeswehr, Municipal Administration, Science (security-oriented) | Median 4.8–5.0 (Blk10); 4.5–5.0 (Blk11) | 0.38–0.44 |
| **Civil-society / constitutional cluster** | Church/Civil Society, Aid Organisations, Communications | Median 1.5–2.2 (Blk10); 1.3–2.0 (Blk11) | 0.31–0.39 |

*W values reported are Kendall's coefficients of concordance for within-cluster item rankings in Round 3, computed separately for each cluster. Both values were statistically significant (p < .001), confirming strong internal rank agreement within each cluster despite between-cluster disagreement. This pattern is consistent with evidence that expert disagreement in Delphi studies can reflect legitimate disciplinary or professional perspectives rather than measurement noise (Diamond et al. 2014; Schifano & Niederberger 2025).*
