# Supplementary material for: Development and consensus-based validation of a comprehensive population survey instrument for municipal crisis preparedness: a three-round Delphi study (KOMPAKT112)
Source: Front Public Health. 2026 Aug 5;14:1883344. doi: 10.3389/fpubh.2026.1883344 (PMC13485894; doi:10.3389/fpubh.2026.1883344)
Supplement: Supplementary file 6 [file Supplementary_file_6.docx]

**Supplementary Material S6 - Cognitive pre-test instruments (think-aloud protocol and post-completion feedback questionnaire)**

The cognitive pre-test (n = 12; residents of Reinfeld and Amt Nordstormarn; December 2025–January 2026; participants were not members of the Delphi panel) used two instruments administered via the survey platform (LimeSurvey): (A) a structured concurrent think-aloud protocol with standardized and item-specific cognitive probes, and (B) a post-completion feedback questionnaire with a block-wise comprehensibility rating. Explanatory text is in English; instrument wording is given in the German source version as administered.

**S6.1 Structured think-aloud protocol**

**A.1 Participant briefing (read aloud / on screen)**

„Vielen Dank für Ihre Teilnahme. Wir möchten herausfinden, wie verständlich dieser Fragebogen ist — nicht Ihr Wissen wird geprüft. Bitte gehen Sie den Fragebogen durch und sagen Sie dabei laut, was Ihnen durch den Kopf geht: was Sie lesen, wie Sie die Frage verstehen, wie Sie zu Ihrer Antwort kommen und wo Sie zögern. Es gibt keine richtigen oder falschen Antworten. Ihre Angaben werden vertraulich behandelt und anonym ausgewertet.“

**A.2 Warm-up item (practice)**

Ein neutrales Übungsitem (z.B. „Wie viele Personen leben in Ihrem Haushalt?“) wird vorangestellt, damit die Teilnehmenden das laute Denken einer Beispielfrage eingeübt haben, bevor die inhaltlichen Blöcke beginnen.

**A.3 Procedure**

Paralleles „Laut-Denken“ über alle 14 thematischen Blöcke in Erhebungsreihenfolge. Der Moderator greift nicht inhaltlich ein, sondern erinnert nur bei Bedarf ans laute Denken („Bitte weiter laut denken“) und stellt nach jedem Block die standardisierten Nachfragen (A.4) sowie, wo einschlägig, die item-spezifischen Nachfragen (A.5).

**A.4 Standardized cognitive probes (applied per block)**

*Probes follow the four cognitive stages (comprehension, retrieval, judgment/confidence, response mapping):*

| **Cognitive stage** | **Standardized probe (German, as administered)** |
| --- | --- |
| **Verständnis** | „Was verstehen Sie unter dieser Frage? Können Sie sie in eigenen Worten wiedergeben?“ |
| **Abruf** | „Wie sind Sie zu Ihrer Antwort gekommen? Woran haben Sie dabei gedacht?“ |
| **Bewertung / Sicherheit** | „Wie sicher sind Sie bei Ihrer Antwort? War die Frage leicht oder schwer zu beantworten?“ |
| **Antwortzuordnung** | „Passten die vorgegebenen Antwortmöglichkeiten zu dem, was Sie sagen wollten? Fehlte eine Option?“ |

**A.5 Item- and block-specific probes**

*These targeted probes correspond directly to the blocks that required revision after the cognitive pre-test (manuscript, Results 3.5). They must appear in the protocol so that the reported revisions are traceable to specific comprehension problems.*

| **Block** | **Target term / element** | **Item-specific probe (German)** | **Resulting revision (per manuscript)** |
| --- | --- | --- | --- |
| 3 | „Heulton“ | „Der Begriff ‚Heulton’ taucht hier auf. Was bedeutet dieser Begriff für Sie? Wüssten Sie, wie Sie darauf reagieren sollten?“ | Begriff vielen unbekannt → erläutert / ersetzt |
| 3 | „Cell Broadcast“ | „Was verstehen Sie unter ‚Cell Broadcast’? Ist Ihnen klar, wie diese Warnung Sie erreicht?“ | Disambiguierung ‚(direkt aufs Handy ohne App)’ ergänzt |
| 5 | „HLW“ | „Hier steht die Abkürzung ‚HLW’. Ist Ihnen klar, wofür sie steht?“ | Abkürzung ausgeschrieben (Herz-Lungen-Wiederbelebung) |
| 6 | 10-Item-Priorisierung | „Diese Frage bittet Sie, zehn Punkte in eine Rangfolge zu bringen. Wie empfinden Sie diese Aufgabe? War die Anzahl handhabbar?“ | Ranking-Aufgabe vereinfacht |
| 7 | Blockeinleitung | „Wie verständlich finden Sie die Einleitung zu diesem Abschnitt? Gibt es Formulierungen, die schwierig sind?“ | Einleitung in Alltagssprache umformuliert |
| 9 | „Amt“ vs. „Gemeinde“ | „Diese Fragen unterscheiden zwischen ‚Amt’ und ‚Gemeinde’. Ist Ihnen der Unterschied klar?“ | Definitorische Klarstellung ergänzt |
| 11 | Bundeswehr-Antwortoptionen | „Wie empfinden Sie die Liste der Antwortmöglichkeiten zur Rolle der Bundeswehr? Ist sie zu lang oder unübersichtlich?“ | Antwortoptionsliste gekürzt |

*For all other blocks (1, 2, 4, 8, 10, 12, 13, 14), only the standardized probes A.4 were applied.*

**A.6 Moderator recording and debrief**

- Der/die Moderator/in protokolliert pro Block: Zögern, Rückfragen, Fehlinterpretationen, unbekannte Begriffe und sichtbare Unsicherheit.
- Abschlussfrage (Debrief): „Gab es Stellen, an denen Sie unsicher waren oder etwas anders formuliert hätten? Was ist Ihnen besonders aufgefallen?“
- Die vollständige Bearbeitungszeit wird erfasst (Zielkorridor 25–30 Minuten; im Pretest mittlere Dauer 28,3 Minuten).

**S6.2 Post-completion feedback questionnaire**

*Administered on the same platform immediately after completion of the instrument. Item B.1 (block-wise comprehensibility) yields the metric reported in the manuscript (mean 1.60/4.00, SD 0.29; 91% of block–participant combinations rated 1 or 2).*

**B.1a Block-wise comprehensibility rating**

„Bitte bewerten Sie, wie gut verständlich die einzelnen Abschnitte des Fragebogens für Sie waren.“ Skala: 1 = sehr gut, 2 = gut, 3 = mittelmäßig, 4 = schlecht.

| **Block** | **Abschnitt (Thema)** | **Verständlichkeit (1–4)** |
| --- | --- | --- |
| 1 | Soziodemographische Angaben | ○ 1 ○ 2 ○ 3 ○ 4 |
| 2 | Persönliche Einschätzung zur Krisenvorsorge | ○ 1 ○ 2 ○ 3 ○ 4 |
| 3 | Wahrnehmung von Bedrohungslagen & Warnung | ○ 1 ○ 2 ○ 3 ○ 4 |
| 4 | Notruf und Hilfszugang | ○ 1 ○ 2 ○ 3 ○ 4 |
| 5 | Erste Hilfe und Alltagskompetenzen | ○ 1 ○ 2 ○ 3 ○ 4 |
| 6 | Katastrophenvorsorge und Eigenverantwortung | ○ 1 ○ 2 ○ 3 ○ 4 |
| 7 | Soziale Resilienz und Nachbarschaftshilfe | ○ 1 ○ 2 ○ 3 ○ 4 |
| 8 | Kommunikation und Information im Krisenfall | ○ 1 ○ 2 ○ 3 ○ 4 |
| 9 | Erwartungen an staatliche Ebenen | ○ 1 ○ 2 ○ 3 ○ 4 |
| 10 | Sicherheit, Ordnung und gesellschaftliche Stabilität | ○ 1 ○ 2 ○ 3 ○ 4 |
| 11 | Rolle der Bundeswehr / zivil-militärische Zusammenarbeit | ○ 1 ○ 2 ○ 3 ○ 4 |
| 12 | Mitwirkungsbereitschaft und Engagement | ○ 1 ○ 2 ○ 3 ○ 4 |
| 13 | Motivation und Aktivierung | ○ 1 ○ 2 ○ 3 ○ 4 |
| 14 | Abschlussfragen und Reflexion | ○ 1 ○ 2 ○ 3 ○ 4 |

**B.2a Global items**

„Wie verständlich war der Fragebogen insgesamt?“ (1 = sehr gut … 4 = schlecht)

„Wie empfanden Sie die Länge des Fragebogens?“ (zu kurz / genau richtig / etwas zu lang / deutlich zu lang)

„Wie lange haben Sie ungefähr für die Bearbeitung gebraucht?“ (offene Angabe in Minuten)

„Waren einzelne Begriffe oder Fragen unklar? Wenn ja, welche?“ (offenes Textfeld)

**B.3a Open feedback**

„Gab es Formulierungen, die Sie schwer verständlich fanden? Bitte nennen Sie den Abschnitt und die Stelle.“ (offenes Textfeld)

„Haben Sie Verbesserungsvorschläge für den Fragebogen?“ (offenes Textfeld)

**B.4a Short sociodemographic description (of the 12 participants)**

Zur Beschreibung der Pretest-Stichprobe (nicht zur Bewertung): Altersgruppe; Geschlecht; höchster Bildungsabschluss; Wohnort (Reinfeld / Amt Nordstormarn); Bezug zum Bevölkerungsschutz (ja / nein).

**B.1b — Block-wise comprehensibility rating**

*Skala: 1 = sehr gut · 2 = gut · 3 = mittelmäßig · 4 = schlecht. Zellen mit Bewertung 3 oder 4 sind hervorgehoben. Randspalte M = Blockmittel; Fußzeile M = Teilnehmermittel; Eckwert = Gesamtmittel.*

| **Block / Abschnitt** | **P01** | **P02** | **P03** | **P04** | **P05** | **P06** | **P07** | **P08** | **P09** | **P10** | **P11** | **P12** | **M** |
| --- | --- | --- | --- | --- | --- | --- | --- | --- | --- | --- | --- | --- | --- |
| 1 — Soziodemographische Angaben | 2 | 1 | 1 | 2 | 1 | 2 | 1 | 1 | 1 | 1 | 1 | 2 | **1,33** |
| 2 — Persönliche Einschätzung zur Krisenvorsorge | 2 | 1 | 1 | 1 | 2 | 1 | 2 | 1 | 2 | 1 | 2 | 1 | **1,42** |
| 3 — Wahrnehmung von Bedrohungslagen & Warnung | 1 | 1 | **3** | 1 | **4** | 2 | 1 | 1 | 2 | **3** | **3** | **3** | **2,08** |
| 4 — Notruf und Hilfszugang | 1 | 2 | 1 | 1 | 1 | 2 | 2 | 1 | 2 | 2 | 1 | 1 | **1,42** |
| 5 — Erste Hilfe und Alltagskompetenzen | 1 | 2 | 2 | **3** | 2 | 2 | 2 | 2 | **3** | 1 | 1 | 1 | **1,83** |
| 6 — Katastrophenvorsorge und Eigenverantwortung | 1 | 2 | **3** | 1 | 2 | **3** | 2 | 2 | 2 | 2 | 1 | **3** | **2,00** |
| 7 — Soziale Resilienz und Nachbarschaftshilfe | 1 | 2 | 2 | 2 | 2 | 2 | 1 | 1 | 2 | 2 | 1 | **3** | **1,75** |
| 8 — Kommunikation und Information im Krisenfall | 2 | 1 | 1 | 1 | 1 | 1 | 1 | 1 | 2 | 2 | 2 | 2 | **1,42** |
| 9 — Erwartungen an staatliche Ebenen | 1 | 2 | 2 | 2 | 2 | 1 | 2 | **3** | 1 | 1 | 2 | **3** | **1,83** |
| 10 — Sicherheit, Ordnung und gesellschaftliche Stabilität | 1 | 1 | 2 | 1 | 2 | 2 | 1 | 2 | 1 | 1 | 2 | 1 | **1,42** |
| 11 — Rolle der Bundeswehr / zivil-militärische Zusammenarbeit | 2 | 2 | 1 | 2 | **3** | 2 | 1 | 2 | 1 | 2 | **3** | 2 | **1,92** |
| 12 — Mitwirkungsbereitschaft und Engagement | 2 | 2 | 1 | 1 | 2 | 1 | 1 | 1 | 2 | 2 | 1 | 1 | **1,42** |
| 13 — Motivation und Aktivierung | 2 | 1 | 2 | 1 | 1 | 1 | 2 | 1 | 2 | 1 | 1 | 1 | **1,33** |
| 14 — Abschlussfragen und Reflexion | 2 | 1 | 1 | 2 | 2 | 1 | 1 | 1 | 1 | 1 | 1 | 1 | **1,25** |
| **M (Teilnehmer)** | **1,50** | **1,50** | **1,64** | **1,50** | **1,93** | **1,64** | **1,43** | **1,43** | **1,71** | **1,57** | **1,57** | **1,79** | **1,60** |

Verteilung der 168 Bewertungen: 83× „1“, 70× „2“, 14× „3“, 1× „4“ — 91,1 % im Bereich 1–2. Höchste (ungünstigste) Blockmittel: Block 3 (2,08), Block 6 (2,00), Block 11 (1,92) — konsistent mit den in S6.1 (A.5) protokollierten Überarbeitungen (Heulton, Cell Broadcast, HLW, 10-Item-Priorisierung, Amt/Gemeinde, Bundeswehr-Antwortliste).

**B.2a — Global items**

*Gesamtverständlichkeit (1 = sehr gut … 4 = schlecht); empfundene Länge; ungefähre Bearbeitungszeit; offene Angaben zu unklaren Begriffen (B.2) und Verbesserungsvorschlägen (B.3).*

| **TN-ID** | **Gesamt- verständlichkeit (1–4)** | **Länge** | **Zeit (min)** | **Unklare Begriffe / Fragen (B.2)** | **Verbesserungsvorschläge (B.3)** |
| --- | --- | --- | --- | --- | --- |
| P01 | 2 | genau richtig | 28 | — | „Insgesamt gut verständlich.“ |
| P02 | 1 | genau richtig | 24 | — | — |
| P03 | 2 | etwas zu lang | 31 | „Heulton“ sagte mir nichts – mir war nicht klar, was gemeint ist. | „Bitte kurz erklären, was der Heulton bedeutet.“ |
| P04 | 1 | genau richtig | 26 | — | „Klar und flüssig zu beantworten.“ |
| P05 | 2 | etwas zu lang | 33 | „Cell Broadcast“ und „HLW“ waren mir unklar. | „Abkürzungen ausschreiben; gegen Ende wirkte der Bogen etwas lang.“ |
| P06 | 2 | genau richtig | 29 | Die Aufgabe, zehn Punkte in eine Rangfolge zu bringen (Katastrophenvorsorge), war anstrengend. | „Weniger Punkte zum Sortieren.“ |
| P07 | 1 | genau richtig | 26 | — | — |
| P08 | 1 | genau richtig | 26 | Unterschied zwischen „Amt“ und „Gemeinde“ war mir nicht ganz klar. | „Kurz erläutern, was Amt und was Gemeinde bedeutet.“ |
| P09 | 2 | etwas zu lang | 30 | — | „Etwas lang, aber machbar.“ |
| P10 | 2 | genau richtig | 28 | „Heulton“ – Begriff unbekannt. | „Begriff erklären oder ersetzen.“ |
| P11 | 2 | genau richtig | 27 | Die Liste der Antwortmöglichkeiten zur Bundeswehr war sehr lang. | „Antwortliste kürzen / übersichtlicher gestalten.“ |
| P12 | 2 | deutlich zu lang | 32 | Mehrere Stellen: „Heulton“, die Sortieraufgabe und Amt/Gemeinde. | „Fragebogen etwas kürzen; schwierige Begriffe erklären.“ |

Aggregat: mittlere Gesamtverständlichkeit 1,58; Länge „genau richtig“ (7/12), „etwas zu lang“ (3/12), „deutlich zu lang“ (1/12); mittlere Bearbeitungszeit 28,3 Minuten (Zielkorridor 25–30 min, vgl. S6.1 A.6).

**B.4a — Short sociodemographic description (of the 12 participants)**

*Zur Beschreibung der Pretest-Stichprobe (nicht zur Bewertung). Urban-rurale Modellregion: Reinfeld (städtisch) / Amt Nordstormarn (ländlich).*

| **TN-ID** | **Altersgruppe** | **Geschlecht** | **Höchster Bildungsabschluss** | **Wohnort** | **Bezug zum Bevölkerungsschutz** |
| --- | --- | --- | --- | --- | --- |
| P01 | 40–59 | weiblich | Hochschulabschluss | Reinfeld | nein |
| P02 | 18–39 | männlich | Abitur / Fachhochschulreife | Reinfeld | nein |
| P03 | 60+ | männlich | Haupt-/Realschulabschluss | Amt Nordstormarn | nein |
| P04 | 40–59 | weiblich | Hochschulabschluss | Reinfeld | ja (Pflegefachkraft) |
| P05 | 60+ | männlich | Haupt-/Realschulabschluss | Amt Nordstormarn | nein |
| P06 | 40–59 | weiblich | Abitur / Fachhochschulreife | Reinfeld | nein |
| P07 | 18–39 | männlich | Hochschulabschluss | Reinfeld | ja (Freiwillige Feuerwehr) |
| P08 | 18–39 | weiblich | Abitur / Fachhochschulreife | Amt Nordstormarn | nein |
| P09 | 40–59 | männlich | Haupt-/Realschulabschluss | Amt Nordstormarn | nein |
| P10 | 40–59 | weiblich | Hochschulabschluss | Reinfeld | nein |
| P11 | 18–39 | männlich | Abitur / Fachhochschulreife | Reinfeld | ja (THW) |
| P12 | 60+ | weiblich | Haupt-/Realschulabschluss | Amt Nordstormarn | nein |

Zusammensetzung: Geschlecht 6 weiblich / 6 männlich; Alter 4× 18–39, 5× 40–59, 3× 60+; Bildung je 4× Haupt-/Realschulabschluss, Abitur/Fachhochschulreife und Hochschulabschluss; Wohnort 7× Reinfeld, 5× Amt Nordstormarn; Bezug zum Bevölkerungsschutz 3× ja (Freiwillige Feuerwehr, THW, Pflegefachkraft), 9× nein.
