# Supplementary material for: Development and consensus-based validation of a comprehensive population survey instrument for municipal crisis preparedness: a three-round Delphi study (KOMPAKT112)
Source: Front Public Health. 2026 Aug 5;14:1883344. doi: 10.3389/fpubh.2026.1883344 (PMC13485894; doi:10.3389/fpubh.2026.1883344)
Supplement: Supplementary file 7 [file Supplementary_file_7.docx]

**Supplementary Material S7 – Population Pre-Test Data Summary**

This supplement reports the full pre-test data collected during the population-based pre-test of the KOMPAKT112 instrument (January–March 2026; n = 50 adult residents of Reinfeld and the Amt Nordstormarn). Data are presented in anonymized, aggregated form consistent with the ethics approval (Ethikkommission der Universität zu Lübeck, AZ 2026-127) and GDPR provisions. No individual-level data are disclosed. The supplement is organized as follows: (1) Sample characteristics; (2) Score distributions (three composite scores and percentile statistics); (3) Score frequency distributions; (4) Subgroup analyses by age, education level, and residence type; (5) Key item frequencies and national benchmark comparisons across all 14 thematic blocks; (6) Sensitization effect and post-survey feedback.

All statistics are descriptive. No inferential tests are reported for this pre-test sample; the sample size (n = 50) was powered for feasibility assessment and comprehensibility evaluation only, not for confirmatory or exploratory factor analyses (see main manuscript §4.5 and Future Research). Full psychometric characterization will be reported using the main survey dataset (target n ≥ 800).

**1. Sample Characteristics**

Table S7.1 presents the anonymized demographic profile of all 50 pre-test participants. Recruitment proceeded via QR-code posters and postcards (placed in public locations in Reinfeld and Nordstormarn), municipal and BOS partner networks, and the KOMPAKT112 social media channels. The sample is not probability-based and is not intended to be representative of the wider population; rather, it reflects the range of population groups present in the project's target communities and provides a feasibility-level assessment of instrument performance across demographic subgroups.

**Table S7.1. Sample characteristics (n = 50; anonymized aggregates).**

| **Characteristic** | **Category** | **n** | **%** | **Note** |
| --- | --- | --- | --- | --- |
| **Gender** | Female | 24 | 48.0% |  |
|  | Male | 24 | 48.0% |  |
|  | Diverse | 2 | 4.0% |  |
| **Age group** | 18–29 years | 8 | 16.0% |  |
|  | 30–44 years | 14 | 28.0% |  |
|  | 45–59 years | 15 | 30.0% |  |
|  | 60–74 years | 10 | 20.0% |  |
|  | ≥75 years | 3 | 6.0% |  |
| **Highest education** | Hauptschulabschluss | 4 | 8.0% |  |
|  | Realschulabschluss | 11 | 22.0% |  |
|  | Abitur / Fachabitur | 15 | 30.0% |  |
|  | University degree | 20 | 40.0% |  |
| **Household size** | 1 person | 10 | 20.0% |  |
|  | 2 persons | 18 | 36.0% |  |
|  | 3 persons | 10 | 20.0% |  |
|  | ≥4 persons | 12 | 24.0% |  |
| **Employment** | Employed (full- or part-time) | 26 | 52.0% |  |
|  | Retired | 9 | 18.0% |  |
|  | Student | 7 | 14.0% |  |
|  | Other / not employed | 8 | 16.0% |  |
| **Residence** | Reinfeld (urban) | 28 | 56.0% | City of Reinfeld |
|  | Nordstormarn (rural) | 22 | 44.0% | Amt Nordstormarn |
| **Recruitment channel** | QR code (posters / postcards) | 18 | 36.0% |  |
|  | Municipal / BOS network | 17 | 34.0% |  |
|  | Social media (project channels) | 9 | 18.0% |  |
|  | Personal referral | 6 | 12.0% |  |
| **Total** | | **50** | **100%** | 100% completion rate; 0 missing values |

*Δ = direction vs. national benchmark; ▲ = above benchmark; ▼ = below benchmark; ~ = within 2 pp; n/a = no comparable national figure available. BBK Forsa = Bundesamt für Bevölkerungsschutz und Katastrophenhilfe, Repräsentative Bevölkerungsbefragung (Forsa) Mai 2022–November 2023. Eurobarometer 2024 = Special Eurobarometer 547 (Germany country fact sheet).*

**2. Score Distributions**

Three composite scores were computed for each participant following the a priori scoring rules defined in the KOMPAKT112 instrument documentation (Supplementary S1): the Vorsorgewissen-Score (Crisis Preparedness Knowledge Score; proportion of knowledge items answered correctly), the Vorsorgeverhalten-Score (Crisis Preparedness Behaviour Score; proportion of enacted preparedness behaviours), and the Gesamt-Vorsorgescore (Composite Preparedness Score; arithmetic mean of both subscales). Score distributions are reported in Table S6.2 with extended percentile statistics not included in Table 4 of the main manuscript.

**Table S7.2. Score statistics for three composite preparedness measures (n = 50).**

| **Score Dimension** | **Mean** | **SD** | **Median** | **P10** | **P25** | **P75** | **P90** | **Min** | **Max** |
| --- | --- | --- | --- | --- | --- | --- | --- | --- | --- |
| **Vorsorgewissen-Score (Crisis Preparedness Knowledge)** | 76.2% | 11.3% | 77.0% | 61.0% | 68.5% | 84.2% | 90.3% | 49.8% | 96.4% |
| **Vorsorgeverhalten-Score (Crisis Preparedness Behaviour)** | 68.9% | 13.7% | 69.5% | 51.2% | 59.8% | 78.3% | 85.6% | 38.5% | 91.2% |
| **Gesamt-Vorsorgescore (Composite Preparedness Score)** | 72.6% | 10.8% | 73.0% | 57.8% | 65.4% | 80.1% | 86.8% | 44.2% | 93.8% |
| **Knowledge–Behaviour Gap (Δ)** | **7.3 percentage points (Vorsorgewissen 76.2% − Vorsorgeverhalten 68.9%); consistent with international evidence base (Amberson et al. 2024; Ni et al. 2025)** | | | | | | | | |

*P10/P25/P75/P90 = 10th, 25th, 75th, and 90th percentiles respectively. SD = standard deviation. Min/Max = observed range. The knowledge–behaviour gap of 7.3 percentage points replicates the pattern documented in large national surveys and is discussed in §4.3 of the main manuscript. No participant scored below 40% on any subscale, confirming the absence of floor effects. No participant scored above 96.4% on any subscale, confirming the absence of ceiling effects.*

**3. Score Frequency Distributions**

Table S7.3 presents the number and proportion of participants falling within each 10-percentage-point score band for all three composite scores. Bar charts (ASCII representation) are included for the knowledge score for visual reference. The distributions confirm adequate score spread across the full range without clustering at extremes.

**Table S7.3. Frequency distribution of composite scores by 10-percentage-point band (n = 50).**

| **Score band** | **Knowledge n** | **Knowledge %** | **Knowledge bar** | **Behaviour n** | **Behaviour %** | **Composite n** | **Composite %** |
| --- | --- | --- | --- | --- | --- | --- | --- |
| **< 50%** | 0 | 0% | □□□□□□□□□□□□□□□□□□□□ 0% | 4% | 0 | 0% | undefined |
| **50–60%** | 2 | 4% | ■□□□□□□□□□□□□□□□□□□□ 4% | 12% | 2 | 4% | undefined |
| **60–70%** | 12 | 24% | ■■■■■□□□□□□□□□□□□□□□ 24% | 28% | 8 | 16% | undefined |
| **70–80%** | 18 | 36% | ■■■■■■■□□□□□□□□□□□□□ 36% | 32% | 20 | 40% | undefined |
| **80–90%** | 14 | 28% | ■■■■■■□□□□□□□□□□□□□□ 28% | 18% | 16 | 32% | undefined |
| **≥90%** | 4 | 8% | ■■□□□□□□□□□□□□□□□□□□ 8% | 6% | 4 | 8% | undefined |

*Bar column: each ■ block represents 5 percentage points of sample coverage (scale 0–100%). Knowledge = Vorsorgewissen-Score; Behaviour = Vorsorgeverhalten-Score; Composite = Gesamt-Vorsorgescore. Participants are counted once per band; no overlaps.*

**4. Subgroup Analyses**

Tables S7.4a–c present mean scores and standard deviations for the three composite score dimensions by age group, educational level, and municipality of residence. These analyses are descriptive only; statistical significance testing was not conducted given the pre-test sample size. Patterns are reported for instrument characterization purposes and to inform hypotheses for the main survey analysis (H3: urban–rural comparison; H4: sociodemographic moderation).

**4a. By age group**

**Table S7.4a. Mean scores by age group (n = 50). Mean (SD).**

| **Subgroup** | **Knowledge Mean (SD)** | **Behaviour Mean (SD)** | **Composite Mean (SD)** | **K-B Gap** |
| --- | --- | --- | --- | --- |
| 18–29 (n=8) | 74.8% (12.1%) | 62.4% (14.8%) | 68.6% (11.6%) | 12.4 pp |
| 30–44 (n=14) | 77.2% (10.8%) | 68.1% (13.2%) | 72.7% (9.9%) | 9.1 pp |
| 45–59 (n=15) | 76.9% (11.4%) | 70.4% (12.9%) | 73.7% (10.3%) | 6.5 pp |
| 60–74 (n=10) | 75.8% (12.3%) | 71.2% (14.1%) | 73.5% (11.4%) | 4.6 pp |
| ≥75 (n=3) | 72.1% (13.6%) | 69.8% (15.3%) | 71.0% (12.8%) | 2.3 pp |
| **Overall (n=50)** | **76.2% (11.3%)** | **68.9% (13.7%)** | **72.6% (10.8%)** | **7.3 pp** |

*K-B Gap = knowledge–behaviour gap (Vorsorgewissen minus Vorsorgeverhalten). Largest gap observed in youngest age group (18–29: 12.4 pp), smallest in oldest (75+: 2.3 pp). This age-gradient is consistent with the life-course hypothesis that older adults’ behaviour is more closely aligned with their knowledge, potentially through accumulated household experience.*

**4b. By educational level**

**Table S7.4b. Mean scores by highest educational qualification (n = 50). Mean (SD).**

| **Subgroup** | **Knowledge Mean (SD)** | **Behaviour Mean (SD)** | **Composite Mean (SD)** | **K-B Gap** |
| --- | --- | --- | --- | --- |
| University degree (n=20) | 79.4% (10.2%) | 72.1% (12.8%) | 75.8% (9.4%) | 7.3 pp |
| Abitur / Fachabitur (n=15) | 76.8% (11.1%) | 68.2% (13.6%) | 72.5% (10.2%) | 8.6 pp |
| Realschulabschluss (n=11) | 74.1% (12.4%) | 66.4% (14.3%) | 70.3% (11.6%) | 7.7 pp |
| **Hauptschulabschluss (n=4)** | **70.2% (13.8%)** | **63.8% (15.2%)** | **67.0% (13.1%)** | **6.4 pp** |

*Monotonic positive gradient observed for both knowledge and behaviour scores with increasing educational attainment. The knowledge–behaviour gap does not follow the same monotonic pattern, suggesting that education narrows the gap only partially and that structural or motivational barriers operate independently of educational level.*

**4c. By municipality of residence (urban vs. rural)**

**Table S7.4c. Mean scores by residence type (n = 50). Mean (SD).**

| **Subgroup** | **Knowledge Mean (SD)** | **Behaviour Mean (SD)** | **Composite Mean (SD)** | **K-B Gap** |
| --- | --- | --- | --- | --- |
| Reinfeld – urban (n=28) | 76.8% (10.9%) | 70.2% (13.1%) | 73.5% (10.2%) | 6.6 pp |
| **Nordstormarn – rural (n=22)** | **75.4% (11.8%)** | **67.2% (14.5%)** | **71.3% (11.4%)** | **8.2 pp** |

*Reinfeld (urban, n = 28) shows marginally higher behaviour scores and a smaller knowledge–behaviour gap (6.6 pp) compared with Nordstormarn (rural, n = 22; 8.2 pp). This directional difference is consistent with the urban–rural comparison hypothesis (H3) embedded in the main survey design. Caution: differences at pre-test level are illustrative only and must not be interpreted as confirmatory evidence; adequate statistical power for H3 requires the main survey sample (target n ≥ 800 with pre-specified urban–rural stratification).*

**5. Key Item Frequencies and National Benchmark Comparisons**

Tables S7.5.1–S7.5.12 present response frequencies for selected indicator items from Blocks 3–14 of the KOMPAKT112 instrument. Items were selected to represent: (a) primary behavioural preparedness indicators for which national benchmark data are available (enabling external validity assessment); (b) items addressing novel dimensions unique to KOMPAKT112 (civil-military cooperation, Block 11; volunteer engagement, Block 12); and (c) items with high variance in the pre-test sample (informative for main survey power calculations). Block 1 (sociodemographic) and Block 2 (personal risk appraisal) data are reported in Tables S6.1 and the subgroup analyses (Section 4) respectively.

Where national benchmark data are available, the direction of deviation (▲ above / ▼ below / ∼ within 2 pp) is indicated. Benchmarks are drawn from the BBK Forsa Bevölkerungsbefragung (May 2022 – November 2023) and the Special Eurobarometer 547 Germany country fact sheet (2024). Full item wording is provided in Supplementary S1; item-level consensus statistics from the Delphi process are provided in Supplementary S2.

**Table S7.5.1. Block 3 – Threat Perception & Warning.**

| **Item (abbreviated)** | **n (Yes / applies)** | **% Sample** | **National benchmark** | **Source** | **Δ** |
| --- | --- | --- | --- | --- | --- |
| NINA warning app installed | 22 | **44%** | ~38% | Eurobarometer 2024 DE | **▲** |
| Bundesweiter Warntag known (aware of event) | 36 | **72%** | ~68% | BBK Forsa 2023 | **▲** |
| All siren signals correctly identified | 9 | **18%** | ~20% | BBK Forsa 2023 | **▼** |
| Cell Broadcast known (without prompting) | 29 | **58%** | n/a | — | **—** |
| Trust in official warning systems (high/v. high) | 31 | **62%** | ~60% | Eurobarometer 2024 | **▲** |

**Table S7.5.2. Block 4 – Emergency Access & Shelter.**

| **Item (abbreviated)** | **n (Yes / applies)** | **% Sample** | **National benchmark** | **Source** | **Δ** |
| --- | --- | --- | --- | --- | --- |
| Knows nearest municipal assembly point | 12 | **24%** | n/a | — | **—** |
| Has written down emergency contacts | 27 | **54%** | ~45% | BBK Forsa 2023 | **▲** |
| Knows shelter-in-place procedure | 18 | **36%** | n/a | — | **—** |
| Has evacuation route planned | 21 | **42%** | ~35% | BBK Forsa 2023 | **▲** |

**Table S7.5.3. Block 5 – First Aid Competencies.**

| **Item (abbreviated)** | **n (Yes / applies)** | **% Sample** | **National benchmark** | **Source** | **Δ** |
| --- | --- | --- | --- | --- | --- |
| First aid course completed within 5 years | 20 | **40%** | ~48% | BBK Forsa 2023 | **▼** |
| Confident performing CPR on adults | 26 | **52%** | n/a | — | **—** |
| Knows emergency number 112 | 49 | **98%** | ~97% | Eurobarometer 2024 | **~** |
| Knows location of nearest AED | 14 | **28%** | n/a | — | **—** |

**Table S7.5.4. Block 6 – Household Preparedness.**

| **Item (abbreviated)** | **n (Yes / applies)** | **% Sample** | **National benchmark** | **Source** | **Δ** |
| --- | --- | --- | --- | --- | --- |
| Food stockpile ≥7 days | 17 | **34%** | ~29% | BBK Forsa 2023 | **▲** |
| Drinking water stockpile ≥3 days | 23 | **46%** | ~38% | BBK Forsa 2023 | **▲** |
| Battery-powered radio available | 19 | **38%** | ~34% | BBK Forsa 2023 | **▲** |
| Flashlight / emergency lighting | 32 | **64%** | ~58% | BBK Forsa 2023 | **▲** |
| Emergency cash reserve (non-card) | 26 | **52%** | ~47% | BBK 2023 | **▲** |
| Medication stockpile (≥14 days, if applicable) | 16 | **32%** | ~28% | BBK Forsa 2023 | **▲** |
| Emergency bag ('Go Bag') assembled | 4 | **8%** | n/a | — | **—** |
| Balcony solar / portable power bank | 11 | **22%** | n/a | — | **—** |

**Table S7.5.5. Block 7 – Social Resilience.**

| **Item (abbreviated)** | **n (Yes / applies)** | **% Sample** | **National benchmark** | **Source** | **Δ** |
| --- | --- | --- | --- | --- | --- |
| Would help elderly neighbours in a crisis | 43 | **86%** | n/a | — | **—** |
| Would share emergency supplies with neighbours | 36 | **72%** | n/a | — | **—** |
| Trust in local community (high/very high) | 29 | **58%** | ~55% | Eurobarometer 2024 | **▲** |
| Active in community / religious organisation | 17 | **34%** | n/a | — | **—** |

**Table S7.5.6. Block 8 – Crisis Communication.**

| **Item (abbreviated)** | **n (Yes / applies)** | **% Sample** | **National benchmark** | **Source** | **Δ** |
| --- | --- | --- | --- | --- | --- |
| Trust in government emergency communication (h/vh) | 27 | **54%** | ~52% | Eurobarometer 2024 | **▲** |
| Uses social media as primary crisis info channel | 22 | **44%** | n/a | — | **—** |
| Concerned about disinformation in crises | 34 | **68%** | n/a | — | **—** |

**Table S7.5.7. Block 9 – Governmental Expectations.**

| **Item (abbreviated)** | **n (Yes / applies)** | **% Sample** | **National benchmark** | **Source** | **Δ** |
| --- | --- | --- | --- | --- | --- |
| Expects government to ensure food supply (crisis) | 41 | **82%** | n/a | — | **—** |
| Expects BOS to arrive within 30 min | 32 | **64%** | n/a | — | **—** |
| Acknowledges own preparedness responsibility | 28 | **56%** | n/a | — | **—** |

**Table S7.5.8. Block 10 – Societal Stability.**

| **Item (abbreviated)** | **n (Yes / applies)** | **% Sample** | **National benchmark** | **Source** | **Δ** |
| --- | --- | --- | --- | --- | --- |
| Would comply with official evacuation order | 44 | **88%** | n/a | — | **—** |
| Would comply with quarantine order | 38 | **76%** | n/a | — | **—** |
| Concerned about social unrest in major crisis | 27 | **54%** | n/a | — | **—** |

**Table S7.5.9. Block 11 – Civil-Military Cooperation.**

| **Item (abbreviated)** | **n (Yes / applies)** | **% Sample** | **National benchmark** | **Source** | **Δ** |
| --- | --- | --- | --- | --- | --- |
| Aware Bundeswehr may operate domestically | 31 | **62%** | n/a | — | **—** |
| Accepts military support in natural disaster | 39 | **78%** | n/a | — | **—** |
| Accepts military support during pandemic | 32 | **64%** | n/a | — | **—** |
| Knows Art. 87a GG limits domestic military use | 9 | **18%** | n/a | — | **—** |

**Table S7.5.10. Block 12 – Volunteer Engagement.**

| **Item (abbreviated)** | **n (Yes / applies)** | **% Sample** | **National benchmark** | **Source** | **Δ** |
| --- | --- | --- | --- | --- | --- |
| Currently active volunteer (emergency domain) | 11 | **22%** | n/a | — | **—** |
| Willing to join local emergency volunteer team | 22 | **44%** | n/a | — | **—** |
| Main barrier: time constraints | 27 | **54%** | n/a | — | **—** |
| Main barrier: lack of confidence / skills | 16 | **32%** | n/a | — | **—** |

**Table S7.5.11. Block 13 – Preparedness Motivation.**

| **Item (abbreviated)** | **n (Yes / applies)** | **% Sample** | **National benchmark** | **Source** | **Δ** |
| --- | --- | --- | --- | --- | --- |
| Has experienced disaster event (minor/major) | 24 | **48%** | n/a | — | **—** |
| Plans to improve preparedness in next 30 days | 33 | **66%** | n/a | post-survey intention | **—** |
| Main barrier: financial cost | 21 | **42%** | n/a | — | **—** |
| Main barrier: lack of time | 19 | **38%** | n/a | — | **—** |

**Table S7.5.12. Block 14 – Post-Survey Reflection.**

| **Item (abbreviated)** | **n (Yes / applies)** | **% Sample** | **National benchmark** | **Source** | **Δ** |
| --- | --- | --- | --- | --- | --- |
| Comprehensibility rated 'very good' | 26 | **52%** | n/a | — | **—** |
| Increased crisis preparedness awareness after survey | 35 | **70%** | n/a | sensitisation effect | **—** |
| Would recommend survey to others | 42 | **84%** | n/a | — | **—** |
| Estimated completion time: ≤30 min | 44 | **88%** | target: 25–30 min | protocol target | **✓** |

*▲ = pre-test frequency above national benchmark; ▼ = below benchmark; ~ = within 2 pp; ✓ = meets protocol target; — = no comparable national benchmark available. National benchmarks: BBK Forsa = BBK Repräsentative Bevölkerungsbefragung 2022–2023 (Bonn: BBK, 2023); Eurobarometer 2024 DE = Special Eurobarometer 547, Germany country fact sheet (European Commission / PreventionWeb, 2024). All pre-test frequencies are unadjusted; no weighting applied. Pre-test sample (n = 50) is not population-representative; comparisons with national benchmarks are for external validity illustration only.*

**6. Sensitization Effect and Post-Survey Feedback (Block 14)**

Block 14 of the KOMPAKT112 instrument functions as a post-completion reflection module designed to capture the extent to which survey participation itself influenced participants’ crisis preparedness awareness — the sensitization effect documented across community preparedness survey research (Inal & Dogan 2025; Gouda & Yang 2023; Hargono et al. 2023). Table S7.6 presents key Block 14 indicators from the pre-test sample.

**Table S7.6. Post-survey reflection indicators — Block 14 summary (n = 50).**

| **Indicator** | **n** | **%** | **Key finding** |
| --- | --- | --- | --- |
| Comprehensibility — rated 'very good' (1/4) | 26 | **52.0%** | No participant rated any block below 2/4 (satisfactory) |
| Comprehensibility — rated 'good' (2/4) | 17 | **34.0%** |  |
| Comprehensibility — rated 'satisfactory' (3/4) | 6 | **12.0%** | Blocks 9 and 11 drove lower ratings (legal terminology) |
| Comprehensibility — rated 'poor' (4/4) | 1 | **2.0%** | Single case; Block 11 civil-military item. Item revised post-pre-test. |
| **Reported increased preparedness awareness after survey** | 35 | **70.0%** | ✓ Sensitization effect confirmed; primary secondary endpoint |
| **Plans concrete preparedness action in next 30 days** | 33 | **66.0%** | Top planned actions: stockpile extension (n=21), NINA app install (n=14) |
| Would recommend survey to friends / family | 42 | **84.0%** | High social acceptability of instrument |
| Estimated completion time ≤30 minutes | 44 | **88.0%** | Mean: 26.4 min (SD 4.8); target range: 25–30 min ✓ |
| Identified a topic as missing from the instrument | 8 | **16.0%** | Most common: cybersecurity/digital resilience (n=4), pet-specific items (n=2) |
| **Completed all items without skipping** | 50 | **100.0%** | No missing data; 0 skip rate across 132 items |

*Sensitization effect operationalized as: self-report of increased crisis preparedness awareness on a binary yes/no item in Block 14 (post-completion). This is a subjective measure and does not capture actual behaviour change; the mechanism (structured attention to preparedness dimensions prompting awareness) is discussed in §4.3 of the main manuscript. The 66% post-survey action intention rate is reported as an observational finding; no follow-up measurement was conducted within the pre-test design.*

**Data availability and ethics statement**

All data presented in this supplement are anonymized aggregated statistics. No individual-level records are published. Raw pre-test data are available from the corresponding author (K. F. Franzen; klaas.franzen@uni-luebeck.de) upon reasonable written request, subject to GDPR provisions and the conditions of the ethics approval (Ethikkommission der Universität zu Lübeck, AZ 2026-127). All pre-test participants provided written informed consent prior to participation, including consent to the use of anonymized aggregate data in scientific publications.
