# Supplementary material for: Development and consensus-based validation of a comprehensive population survey instrument for municipal crisis preparedness: a three-round Delphi study (KOMPAKT112)
Source: Front Public Health. 2026 Aug 5;14:1883344. doi: 10.3389/fpubh.2026.1883344 (PMC13485894; doi:10.3389/fpubh.2026.1883344)
Supplement: Supplementary file 8 [file Supplementary_file_8.docx]

**Supplementary Material S8 — Preliminary internal-consistency results (reflective subscales) and composite scoring key**

This supplement consolidates two items requested during revision: (S8.1) preliminary block-level internal consistency estimated from the population pre-test (n = 50), and (S8.2) the full construction and scoring key of the Vorsorgewissen (knowledge), Vorsorgeverhalten (behavior), and composite Gesamt-Vorsorge scores. The item wording is retained in the German source version, and the explanatory text is in English.

**S8.1 Preliminary internal consistency (reflective subscales)**

Although comprehensive psychometric characterization (confirmatory factor analysis, measurement invariance, test–retest reliability) was reserved for the main survey (n ≥ 800), preliminary block-level internal consistency was estimated from the population pre-test (n = 50) for blocks that constitute reflective Likert batteries. Standardized Cronbach’s α was reported with 2,000-sample bootstrap 95% confidence intervals. Blocks comprising heterogeneous single-indicator or multi-select items are formative rather than reflective, and internal consistency coefficients are not appropriate for them. Test–retest reliability (ICC) could not be estimated because the pre-test used a single administration only.

**Table S8.1a Standardized Cronbach’s α for reflective subscales (pre-test, n = 50)**

| **Block** | **Scale / subscale** | **k items** | **n** | **α (std.)** | **95% CI (bootstrap)** | **Interpretation** |
| --- | --- | --- | --- | --- | --- | --- |
| 5 | Erste-Hilfe-Kompetenz (Selbsteinschätzung, 4-stufig) | 7 | 47 | 0.922 | [0.87; 0.95] | excellent |
| 6 | Haushaltsvorrat-Inventar (3-stufig) | 10 | 46 | 0.856 | [0.78; 0.90] | good |
| 9 | Vertrauen/Erwartungen staatl. Ebenen (5-stufig) | 6 | 48 | 0.825 | [0.68; 0.89] | good |
| 12a | Engagement-Motivatoren (5-stufig) | 10 | 49 | 0.760 | [0.56; 0.85] | acceptable |
| 12b | Engagement-Barrieren (5-stufig) | 10 | 49 | 0.773 | [0.63; 0.85] | acceptable |

*Blocks 5 and 6 were the two primary endpoints of the main survey (first-aid competence and household preparedness). Block 12 was analyzed as two separate reflective grids: 12a) engagement motivators and 12b) engagement barriers.*

**Table S8.1b Measurement model by block**

| **Block** | **Domain** | **Reflective Likert items** | **Measurement model / reliability approach** |
| --- | --- | --- | --- |
| 1 | Sociodemographic | 0 | Descriptive (sociodemographic) — no scale score |
| 2 | Personal risk appraisal | 1 | Heterogeneous / formative (single indicators, multi-select) — α not appropriate |
| 3 | Threat perception & warning | 2 | Heterogeneous / formative (single indicators, multi-select) — α not appropriate |
| 4 | Emergency access & shelter | 0 | Heterogeneous / formative (single indicators, multi-select) — α not appropriate |
| 5 | First-aid competencies | 7 | Reflective Likert battery → α reported |
| 6 | Household preparedness | 10 | Reflective Likert battery → α reported |
| 7 | Social resilience | 0 | Heterogeneous / formative (single indicators, multi-select) — α not appropriate |
| 8 | Crisis communication | 0 | Heterogeneous / formative (single indicators, multi-select) — α not appropriate |
| 9 | Governmental expectations | 6 | Reflective Likert battery → α reported |
| 10 | Societal stability | 2 | Heterogeneous / formative (single indicators, multi-select) — α not appropriate |
| 11 | Civil-military cooperation | 0 | Only 2 reflective items → inter-item correlation only |
| 12 | Volunteer engagement | 20 | Two reflective grids (motivators / barriers) → α reported separately (12a, 12b) |
| 13 | Preparedness motivation | 0 | Heterogeneous / formative (single indicators, multi-select) — α not appropriate |
| 14 | Post-survey reflection | 0 | Heterogeneous / formative (single indicators, multi-select) — α not appropriate |

**S8.2 Composite scoring key**

**Construction principle**

The two subscales are block-crossing indices, not block-wise indices. The 14 thematic blocks structure the content of the instrument, and the scores are formed by pooling the content-relevant knowledge- and behavior-type items from several blocks. Each item is first mapped onto a uniform 0–1 metric (item-specific coding following the gradation of the respective response options; see tables below), which renders heterogeneous response formats (dichotomous, 3-/4-/5-point, multi-select) comparable. Within each subscale, the item scores were averaged and multiplied by 100, yielding a 0–100% value.

The Vorsorgewissen (knowledge) score = mean of knowledge-item scores × 100. Vorsorgeverhalten (behavior) score = mean of behavior-item scores × 100. Gesamtvorsorge (composite) score = unweighted arithmetic mean of the two subscale percentages (consistent with the footnote to Table 4). Missing or not-applicable responses (e.g., “no vehicle” for the in-car first-aid kit, “don’t know”) were excluded before averaging; thus, the score was based on the answered items.

**Table S8.2a Scoring key — Vorsorgewissen (knowledge) subscale**

| **Item (question, German source)** | **Block** | **Coding (response → score 0–1)** |
| --- | --- | --- |
| Bedeutung von Sirenensignalen | 3 | all signals=1.0; some=0.5; “know they differ”=0.25; none=0 |
| Bedeutung „Heulton“ | 3 | know exactly=1.0; rough idea=0.5; don’t know=0 |
| Kenntnis Cell Broadcast | 3 | know how it works=1.0; heard of it=0.5; unknown=0 |
| Bekannte Notrufnummern (Mehrfachauswahl) | 4 | share of valid numbers correctly identified (for example, 112, 110) |
| Korrekte Verwendung der 112 (Mehrfachauswahl) | 4 | share of emergency scenarios correctly assigned |
| Alternative Kontaktmöglichkeiten (nicht lebensbedrohlich) | 4 | several=1.0; one=0.5; “would search online”=0.25; “always 112”=0 |
| Anlaufpunkte im Krisenfall – wissen wo | 4 | “know exactly”=1.0; guess=0.5; don’t know=0 |
| Erste-Hilfe-Kenntnisse (7-Item-Array, Mittelwert) | 5 | very good=1.0; good=0.67; moderate=0.33; poor/none=0 |
| Kenntnis der BBK-Empfehlungen | 6 | intensive=1.0; superficial=0.67; heard of=0.33; unknown=0 |
| Kenntnis Trinkwasser-Empfehlung (2 l/Person/Tag) | 6 | know + adhere=1.0; know, not adhere=0.5; didn’t know=0 |
| Trinkwasserbeschaffung im Notfall – wissen wie | 6 | several ways=1.0; one idea=0.5; “rely on stores”=0.25; don’t know=0 |

*Knowledge was operationalized as self-reported familiarity/recognition (not objectively tested factual knowledge); two items (emergency numbers, use of 112) captured a more objective recognition share via the hit rate on multi-select options.*

**Table S8.2b Scoring key — Vorsorgeverhalten (behavior) subscale**

| **Item (question, German source)** | **Block** | **Coding (response → score 0–1)** |
| --- | --- | --- |
| Sich über Warnsysteme informiert | 3 | in detail=1.0; partly=0.5; “interested but no”=0; “no need”=0 |
| Erste-Hilfe-Kurs absolviert | 5 | last 2 yr=1.0; 3–5 yr=0.67; 6–10 yr=0.33; >10 yr=0.17; none=0 |
| Erste-Hilfe-Kasten zu Hause | 5 | complete/current=1.0; present, unsure=0.5; none=0 |
| Erste-Hilfe-Kasten im Fahrzeug | 5 | checked regularly=1.0; not checked long=0.5; no vehicle=missing (excluded) |
| Lebensmittelvorräte (Reichweite in Tagen) | 6 | >10 days=1.0; 7–10=0.75; 4–6=0.5; 1–3=0.25; <1 day=0 |
| Vorrats-Inventar (10 Items, Mittelwert) | 6 | “sufficient”=1.0; “some”=0.5; “no”=0 |
| Wichtige Dokumente griffbereit | 6 | emergency folder=1.0; partly=0.5; “know where they are”=0.25; not considered=0 |
| Bargeldreserven für den Notfall | 6 | sufficient (several days)=1.0; small reserve=0.5; no=0 |
| Alternative Energiequellen vorhanden | 6 | yes=1.0; no=0 |
| Warn-App(s) installiert | 8 | yes=1.0; no=0 |
| Eigenschutz-Maßnahmen getroffen | 10 | concrete measures taken=1.0; considered/not implemented=0.5; no=0 |

*Behavior is operationalized as self-reported enactment with graded recency/completeness (e.g., recency of the first-aid course, days of supply coverage).*

**Note on cross-instrument comparability**

The operationalization of “knowledge” and “behavior” is instrument specific. Benchmark instruments (HEPI, EPC, FEMA National Household Survey) differ in terms of item selection, response formats, scoring rules, and the knowledge–behavior boundary. The absolute magnitude of the observed 7.3-percentage-point knowledge–behavior gap is therefore not directly comparable across instruments; only the qualitative pattern of reported knowledge exceeding enacted behavior is transferable to the current study. Given the non-representative pre-test sample (n = 50), the gap is an internal, exploratory quantity, not a population-level or cross-instrument benchmark.
